# Tetrahedral *M* ^II^_4_L_6_ (*M* = Fe, Zn) Cages Equipped with Walls Featuring Antiaromatic Pentalene Motifs

**DOI:** 10.1021/jacsau.6c00720

**Published:** 2026-07-15

**Authors:** Wojciech Stawski, Birgit Esser

**Affiliations:** † Institute of Organic Chemistry II and Advanced Materials, 9189 Ulm University, Albert-Einstein-Allee 11, 89081 Ulm, Germany; ‡ CELEST Green Energy Lab Ulm, Ulm University, Lise-Meitner Str. 16, 89081 Ulm, Germany

**Keywords:** antiaromaticity, cage, pentalene, supramolecular chemistry, benzannulation

## Abstract

Antiaromatic compounds are often difficult to work with due to their relative instability in comparison with aromatic analogues. As a consequence, their application in functional materials remains in its infancy. Here, we report the synthesis of porous *M* ^II^_4_L_6_ (*M* = Fe, Zn) coordination cages equipped with dibenzo­[*a*,*e*]­pentalene panels involving the antiaromatic motif of pentalene stabilized by benzannulation. Both cages encapsulate fullerene C_60_, with the more adaptable zinc­(II) cage also binding C_70_, undergoing pronounced structural reconfiguration upon C_60_ inclusion, and displaying broader affinity for polycyclic aromatic hydrocarbons. The cages selectively bind anthraquinone over its reduced form and interact with steroids such as testosterone and cholesterol. Remarkably, the chemical shifts of the guests are affected by the aromatic Clar sextets rather than the antiaromatic pentalene motif. These assemblies represent only the second reported example of a supramolecular cage with a cavity fully enclosed by antiaromatic walls and demonstrate that benzannulated antiaromatic motifs can be incorporated into cages without interrupting conventional host–guest behavior, while simultaneously allowing for a systematic tuning of magnetic shielding effects.

## Graphical abstract

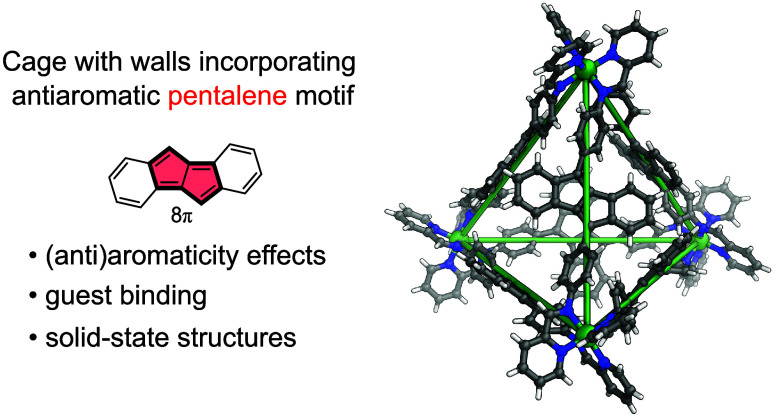

## 1 Introduction

Antiaromatic molecules are fascinating, yet, frequently, elusive π-conjugated species.^1–3^ A common feature of antiaromatic molecules is the presence of a paratropic ring current observed in NMR experiments, which flows in the opposite direction to the diatropic ring current induced in aromatic molecules.^4^ Antiaromatic compounds typically have low LUMO and/or high HOMO energies, which is useful for the design of molecular wires;^5–7^ another common feature is their propensity to undergo two-electron redox reactions, which stabilizes the π-system by switching from antiaromatic to aromatic character.^8–15^ Certain antiaromatic compounds exhibit significant open-shell character and many are rather unstable;^16,17^ several design principles have been developed to stabilize them. For instance, the pristine pentalene (Figure 1a, top) was isolated only in an argon matrix at 20 K.^18^ Later, it was found that substitution with bulky *tert*-butyl groups drastically increases its stability.^19^ Another commonly used strategy for stabilizing antiaromatic species is fusion with aromatic rings.^20–23^ Fusion of pentalene with two benzene rings leads to dibenzo­[*a*,*e*]­pentalene (DBP; Figure 1a, bottom).^24^ The electrons formally belonging to the pentalene are now torn between participating in the local antiaromatic 8π circuit or stabilizing two aromatic Clar sextets. This leads to a decrease of antiaromaticity in the pentalene core, manifested by a reduced magnetic shielding, and an overall stabilization.^25,26^ The DBP motif has been successfully imprinted into cycloparaphenylenes, forming conjugated nanohoops with antiaromatic panels.^27–32^

**Figure 1:**
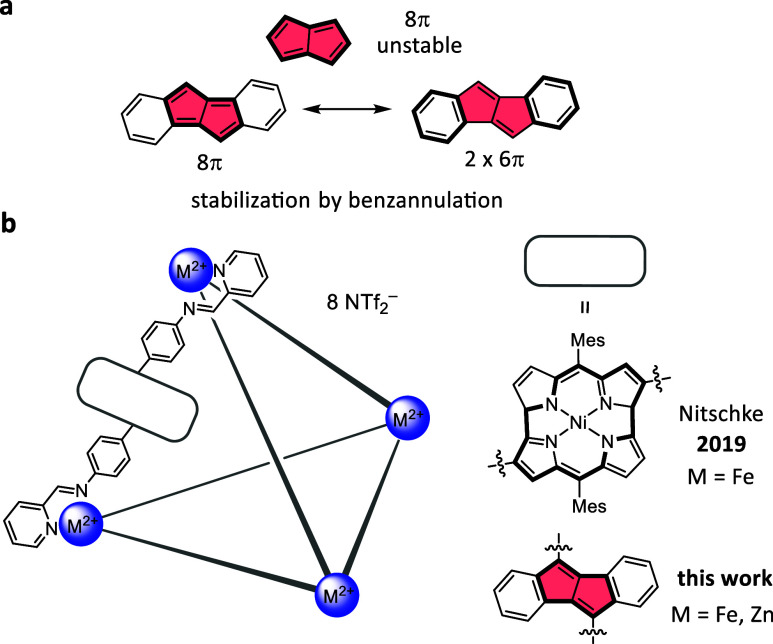
(a) Schematic representation of pentalene (top) and resonance structures of dibenzo­[a,e]­pentalene with delocalization pathways indicated in bold and the pentalene motif in red. (b) Schematic representation of tetrahedral M^II^_4_L_6_ cages reported in the literature^38^ and in this work. NTf_2_^–^ = bis­(trifluoromethanesulfonyl)­imide.

Recently, Dumele, Esser, and co-workers reported porous 2D covalent-organic frameworks (COFs) featuring DBP linkers with ambipolar redox behavior and used them as positive electrodes in Li-based batteries.^33^

Metal-organic coordination cages are fascinating nanosized vessels capable of stabilizing reactive guests, catalyzing reactions through nanoconfinement, and enabling stereoselective transformations.^34–37^ Despite remarkable progress in the field, the vast majority of reported cages rely on aromatic panels. Only one notable exception exists: a supramolecular coordination cage incorporating antiaromatic norcorrole panels, reported by Nitschke and co-workers.^38^ This pioneering system demonstrated that encapsulated guests experience a dramatic deshielding (up to 26 ppm) due to the paratropic ring current of the norcorrole walls, along with altered *T*_1_ relaxation times, allowing the cage to function as a powerful chemical-shift reagent.

Apart from modulating chemical shifts of the encapsulated guests, embedding antiaromatic units into a cage structure is potentially interesting for other reasons: a) antiaromatic systems are intrinsically easier to oxidize and reduce than their aromatic counterparts and act as redox amphoters; this redox accessibility introduces a potential external stimulus to modulate host-guest affinity and release^33^ and b) antiaromatic systems often have perturbed HOMO/LUMO energy levels when compared with aromatic analogues; cages incorporating them could, therefore, function as porous materials with interesting photophysical properties, for instance, for improved charge transfer.^39^

Here, we report on the synthesis of coordination cages equipped with DBP walls (Figure 1b). Notably, these cages constitute only a second example of a supramolecular coordination cage with antiaromatic panels, following the pioneering norcorrole-based cage reported by Nitschke and co-workers.^38^ Qualitative binding tests with a variety of guests show that incorporation of formally antiaromatic motifs into the cage walls does not inevitably lead to magnetic deshielding within the cage cavity; this can be avoided, provided that the antiaromatic fragment is appropriately modified—here, through double benzannulation of pentalene.

## 2 Results and Discussion

### 2.1 Synthesis of the Building Block

To assemble the cage, we synthesized the bis-aniline building block **3** by hydrolyzing the literature-known DBP derivative **1** with HCl (Figure 2a).^33^ The resulting hydrochloride salt **2** was then neutralized with NaOH, providing bis-aniline **3**, whose structure was unambiguously confirmed by single-crystal X-ray diffraction analysis (Figure 2a).

**Figure 2:**
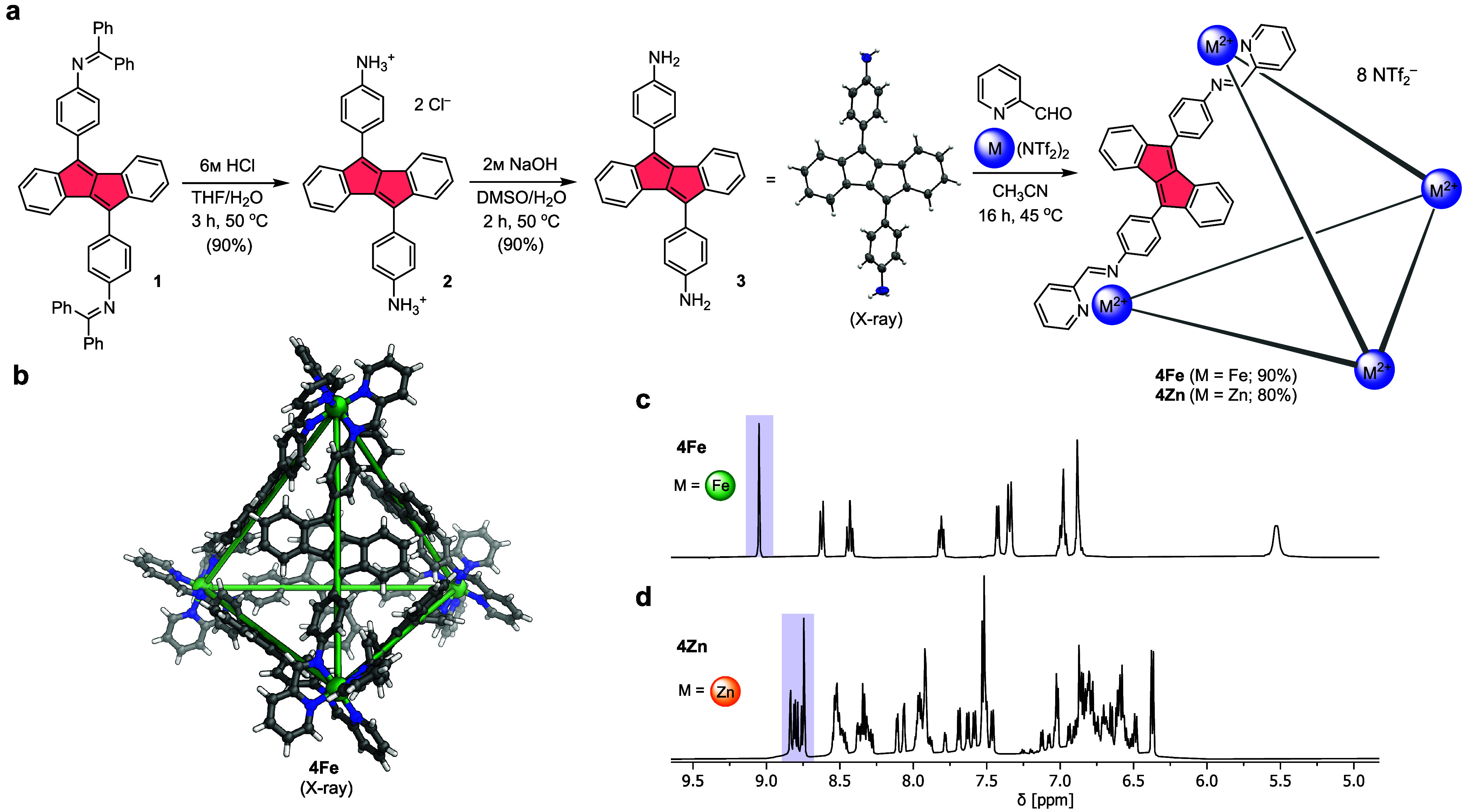
Synthesis of building blocks and their self-assembly to form metal-organic cages. (a) Synthetic route to the cages **4Fe** and **4Zn**; (b) crystal structure of the cationic part of **4Fe** as a carborane salt, depicting the all-Δ cage isomer; and ^1^H NMR spectra of cages (c) **4Fe** and (d) **4Zn** (CD_3_CN, 600 MHz, 25 °C) with imine peaks highlighted in blue. For signals’ assignment, see Supporting Information. Color code: carbon-gray, nitrogen-blue, iron-green, and hydrogen-white. In the crystal structure of **3**, displacement ellipsoids are displayed at 50% level and the minor disorder component was omitted for clarity.

### 2.2 Subcomponent Self-Assembly

Bis-aniline **3** reacted with 2-formylpyridine in the presence of iron­(II) or zinc­(II) triflimides,^40^ forming intensively colored solutions of cages **4Fe** (red-brown) and **4Zn** (orange). ^1^H NMR spectra indicated only one imine peak for **4Fe** (δ_H_ = 9.05 ppm), whereas **4Zn** showed eight imine peaks in the range of δ_H_ = 8.83–8.74 ppm. This indicates that the iron­(II) cage is formed as a fully symmetrical, tetrahedral (*T*) isomer (with all-Λ and all-Δ metal corners), whereas self-assembly with zinc­(II) leads to an equilibrium mixture of all three possible cage isomer sets, depending on the configuration (Λ/Δ) around the metal centers: *T* (one imine peak), *C*_3_ (four imine peaks), and *S*_4_ (three imine peaks).^41^ Deconvolution of the imine region (Figure S18) allowed estimating the amount of the *T* isomer in the overall mixture to be 26%, which is higher than the 12.5% expected from a statistical mixture of all isomers. This suggests some degree of thermodynamic preference for this form. ^19^F NMR spectroscopy of both **4Fe** and **4Zn** showed one signal at ca. δ_F_ = −80.1 ppm, indicating that the triflimide counterion is not encapsulated (Figures S7 and S19).

Mass spectrometry with electrospray ionization (ESI MS) performed for **4Fe** showed a set of signals corresponding to octa-, hepta-, tetra- and tri-cations, matching the Fe_4_L_6_ stoichiometry (see Figure S14). The zinc­(II) cage **4Zn** could not be detected by ESI MS, most likely due to its more labile character based on the Zn ions.^42–44^

Formation of the Fe_4_L_6_ cage was unambiguously confirmed by single-crystal X-ray diffraction analysis of a specimen grown by vapor diffusion of benzene into a solution of **4Fe** in acetonitrile containing an excess of cesium carborane (Figure 2b), which was added to facilitate crystallization. The cage crystallized in a centrosymmetric *C*2/*c* space group with the entire molecule in the asymmetric unit. All four corners have the same handedness (all- Λ and all- Δ), consistent with the high symmetry of the ^1^H NMR spectrum in solution. The Fe­(II) vertices are separated by 17.38–17.59 Å, and the void volume inside the cage is 565 Å^3^ (Figure S108; calculated with a probe radius of 2.5 Å using MoloVol software).^45^ **4Zn** only formed films or amorphous precipitates, however, we were able to crystallize it as a supramolecular complex with C_60_ fullerene (see the next section: Fullerene binding).

### 2.3 Fullerene binding

The presence of large voids in the structure predefines the cages as supramolecular hosts for fullerenes.^46–48^ We monitored the reaction between **4Fe** and **4Zn** with C_60_ and C_70_ in CD_3_CN by ^1^H NMR spectroscopy. Binding was inferred from shifts of the host’s signals and/or its symmetry. In the case of **4Zn**, the complexation with C_60_ was complete already after 1 h of slowly rotating the NMR tube at 20 °C (Figure S33), whereas **4Fe** required vigorous stirring overnight at 50 °C (Figure S28). The longer reaction time and higher temperature required for **4Fe** is consistent with the fact that iron­(II) forms stronger complexes than zinc­(II), making them less labile and, therefore, less prone to structural changes in order to accommodate guest molecules.^42–44^

The ^1^H NMR spectrum of **4Fe·C**_**60**_ shows several imine peaks (Figure 3a), with the most intense one corresponding to the *T* isomer, indicating that most of the cage retained its initial symmetry and some of material likely underwent reconfiguration. On the other hand, complexation of C_60_ by **4Zn** leads to almost full reconfiguration of the cage from a mixture of isomers to the *T* isomer in **4Zn·C**_**60**_ (Figure 3c).

**Figure 3:**
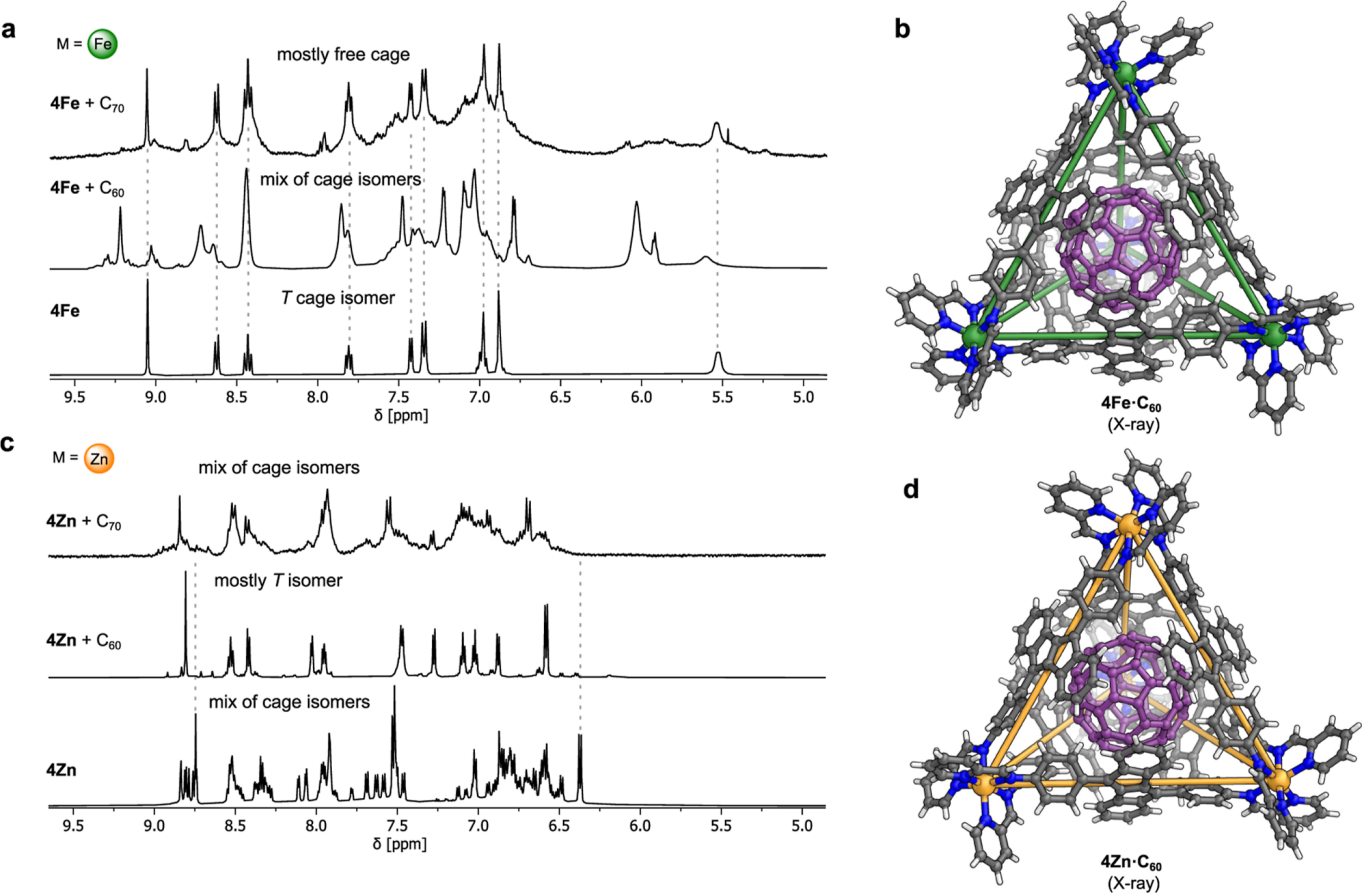
Fullerene binding experiments. (a) Comparison of ^1^H NMR spectra of empty cage **4Fe** and mixtures after stirring the cage with fullerenes C_60_ and C_70_ overnight at 50 °C (CD_3_CN, 400 MHz, 25 °C); (b) crystal structure of the cationic part of **4Fe·C**_**60**_ depicting the all-Λ cage isomer; (c) comparison of the ^1^H NMR spectra of empty cage **4Zn** and mixtures after stirring the cage with fullerenes C_60_ and C_70_ overnight at 50 °C (CD_3_CN, 400 MHz, 25 °C); and (d) crystal structure of the cationic part of **4Zn·C**_**60**_ depicting the all-Δ cage isomer. Minor disorder components in the crystal structures were omitted for clarity. Color code: carbon-gray, nitrogen-blue, iron-green, zinc-orange, and hydrogen-white. Encapsulated C_60_ molecules are shown in violet.

Both products **4Fe·C**_**60**_ and **4Zn·C**_**60**_ were detected in ESI MS measurements (Figures S30 and S42). It is worth noting that the empty cage **4Zn** was not observed in the MS experiments, whereas its C_60_ complex was detected—this suggests that encapsulation of C_60_ stabilizes the cage architecture in the gas phase. ^13^C NMR analysis showed peaks at ca. δ_C_ = 143.0–142.6 ppm (**4Fe·C**_**60**_, Figure S29) and δ_C_ = 142.8–142.7 ppm (**4Zn·C**_**60**_, Figure S35) indicating that the cages contain C_60_, which otherwise is completely insoluble in acetonitrile.^49^ These chemical shifts are rather typical, and the fullerene signal does not exhibit strong deshielding by the antiaromatic pentalene motif.^50^

Fullerene C_70_, with its oval shape, has a worse fit to the cage cavity—the ^1^H NMR spectra indicate that most of the cage **4Fe** remained intact even after a week of stirring at 50 °C. Addition of 1,2-dichlorobenzene (a good solvent for C_70_) did not improve encapsulation (Figures 3a and S46). In contrast, **4Zn** formed the inclusion complex **4Zn·C**_**70**_ (Figure 3c), yet the conversion was slower than with C_60_ (Figure S47), requiring overnight stirring at 50 °C instead of simple shaking for 1 h at room temperature. The obtained ^1^H NMR spectrum suggests prevalence of the *T* isomer yet displays more imine signals than expected for a single species. This is indicative of the presence of other isomers and possibly symmetry breaking in these isomers by the low symmetry of C_70_ and its slow tumbling on the NMR time scale. Guest displacement experiments performed for **4Zn** demonstrated that encapsulated C_70_ can be replaced by C_60_, indicating that C_60_ binds more strongly than C_70_ (Figures S49 and S50). The thermodynamic vs kinetic favorability for the C_60_ complex was additionally confirmed by monitoring complexation using a 1:1 molar mixture of both fullerenes, during which only the **4Zn·C**_**60**_ complex was observed, even after reacting in a microwave reactor at 140 °C for 1 h (Figure S51).

Single crystals of **4Fe·C**_**60**_ and **4Zn·C**_**60**_ suitable for synchrotron X-ray diffraction were grown by vapor diffusion of benzene into solutions of the fullerene complexes in acetonitrile containing an excess of cesium carborane (Figure 3b,d). Both inclusion complexes crystallized as *T* isomers in centrosymmetric space groups, meaning that the crystals contained both enantiomers (all-Λ and all-Δ). In both cases, the DBP units are slightly curved, which improves stacking interactions with C_60_ molecules. The closest interatomic fullerene-DBP distances are 3.19 Å (**4Fe·C**_**60**_) and 3.14 Å (**4Zn·C**_**60**_).

### 2.4 Small-Molecule Binding

We performed a qualitative guest-binding screening on both cages **4Fe** and **4Zn**, using a variety of guests. Both cages bind certain polycyclic aromatic hydrocarbons (PAHs) and examples of naturally occurring steroids (testosterone, cholesterol), as depicted in Figure 4a. We chose these guests to screen different types of molecules with varying size, planarity, and solubility, and these guests are commonly used for similar systems.^50,51^ Binding studies were performed following a standard protocol established for similar cages.^50,51^ We added an excess of guest (typically 4–8 equiv) to the solution of the cage (ca. 1 mM) in CD_3_CN, and the mixture was stirred overnight at 20 °C. ^1^H NMR spectra were then recorded. Typically, we used an excess of guest rather than gradually following the binding progress because especially larger PAHs and cholesterol have a low solubility in CD_3_CN. Then, we assigned the guests as binding/not binding to the cages depending on whether the spectra fulfilled at least one of the following conditions, as reported in the literature for related systems: (a) shifts of guest signals of δ_H_ > 0.10 ppm were observed, (b) a new set of signals for the encapsulated guest appeared, or (c) the symmetry of the cage changed.

**Figure 4:**
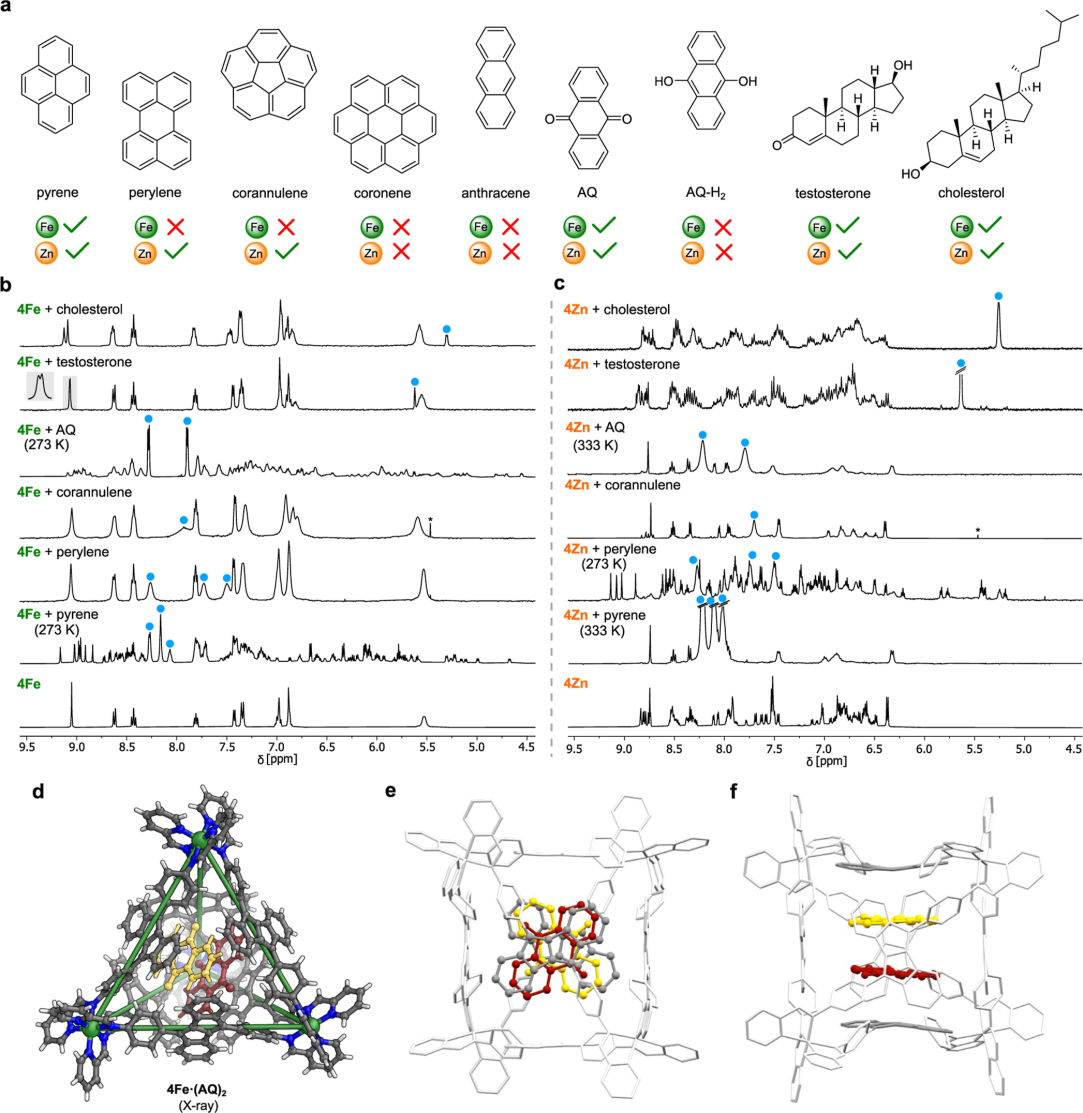
Small-molecule binding. (a) Overview of tested compounds and their binding ability within the cages. (b,c) Comparison of ^1^H NMR spectra of the empty cage and after addition of selected guests of **4Fe** (b) and **4Zn** (c) (600 MHz, 25 °C, CD_3_CN, if not stated otherwise. The blue dot indicates the signal of the excess of the guest. The amount of the added guests varied between 4–8 equiv with the exact amount difficult to determine due to the low solubility of most of the guest molecules in acetonitrile. Some spectra were measured at temperatures different than 25 °C due to signal broadening observed at 25 °C (for details, see Supporting Information). An asterisk denotes a residual CH_2_Cl_2_ impurity coming from the corannulene sample. The gray-highlighted inset in the sample of **4Fe** containing testosterone is a zoom-in on the imine signal). (d) View on the cationic part of the crystal structure of **4Fe·(AQ)**_**2**_ with encapsulated **AQ** molecules indicated in yellow and red, showing the all-Δ isomer. (e,f) Two orthogonal views on the crystal structure of **4Fe·(AQ)**_**2**_ highlighting stacking of the **AQ** molecules (red and yellow balls and sticks) and nonplanar DBP units (dark gray balls and sticks). The other two DBP units are planar.

Addition of pyrene to **4Fe** caused desymmetrization of the host, with the sharpest peaks observed at *T* = 273 K (Figure S53); eight imine peaks can be distinguished. Rotating Frame Overhauser Effect Spectroscopy (ROESY) indicated exchange correlations between the excess of free and the encapsulated guest at δ_H_ = 5.75–6.67 ppm (Figure S56), consistent with a guest exchange slow on the NMR time scale.^52^ In contrast, **4Zn** gives rather broad spectra that become sharper upon increasing temperature: at *T* = 333 K one sharp imine peak is observed, indicating prevalence of the *T* isomer as opposed to the free cage (Figure S59). The observed temperature dependence is consistent with an intermediate rate of guest exchange at lower temperatures and a fast exchange at higher temperatures. The low symmetry of the assemblies at low temperatures might also be influenced by slow tumbling of the guest molecules on the NMR time scale.

Perylene shows small chemical shifts (up to 0.05 ppm, at 298 K) and some signal broadening upon addition to **4Fe**, pointing toward only weak π-stacking interactions with the host. On the other hand, addition of perylene to **4Zn** leads to reconfiguration of the host, forming mostly the *T* isomer at 298 K (Figure S61). This is consistent with a general trend that Zn­(II) complexes are more labile than Fe­(II) analogues and, therefore, they are better able to adapt their geometry to the guest.^42–44^ Lowering temperature causes desymmetrization of the host-guest complex with formation of four imine peaks at *T* = 273 K. ROESY confirmed slow guest exchange by the presence of the exchange correlations to the free guest’s signals (Figure S66).

The more π-extended, bowl-shaped corannulene does not show binding to **4Fe**, as the guest signals only get broader with added guest (Figure S67) with negligible shifts. At the same time, **4Zn** undergoes reconfiguration to a mix of isomers where the T isomer dominates, followed by a broadening and an upfield-shift of the guest signal by 0.25 ppm. The corannulene peak does not show exchange correlations, and the observed changes are consistent with fast guest exchange (Figure S72).

Larger and planar coronene does not show binding to any of the cages (Figures S73 and S74). Smaller anthracene does not bind either (Figures S75 and S76). However, anthraquinone (**AQ**) shows binding to both **4Fe** and **4Zn** (Figure 4b,c). Binding causes desymmetrization of **4Fe** with clear EXSY peaks from the excess of guest, pointing toward slow guest exchange (Figure S79). On the other hand, spectra of **4Zn** become broad upon addition of **AQ**, pointing toward an intermediate rate of exchange. Due to the low solubility of **AQ** in CH_3_CN, its signals do not get sharper even after addition of a large excess (50 equiv) of guest to **4Zn**. However, sharpening of all signals is observed at higher temperatures, consistent with a fast-guest exchange regime (Figure S82). The differences in the binding behavior and stability of the host-guest complexes are also visible when precipitating the cages with diethyl ether from their solutions in acetonitrile: **4Fe**, which binds **AQ** in a slow exchange regime, holds the **AQ** encapsulated, whereas in the case of **4Zn** that binds **AQ** in an intermediate/fast exchange, the empty cage is recovered (Figures S80 and S83), suggesting that **AQ** is kinetically bound in **4Fe** but not in **4Zn**.

Diffraction experiments on crystals grown by vapor diffusion of benzene into a solution of **4Fe** containing an excess of anthraquinone and cesium carborane revealed that **4Fe** crystallizes with all-Δ *a*nd all-Λ configurations (centrosymmetric space group *P*2_1_/*c*) and encapsulates two anthraquinone molecules (Figure 4d) with an additional one found in the lattice. Encapsulated **AQ**s stack on top of each other with a twist angle of 80° and a centroid-to-centroid distance of 3.63 Å (Figure 4e). **AQ**s also stack to the DBP walls (centroid-to-centroid distances of 3.63 and 3.67 Å), in which the pentalene motif is slightly tilted toward the inside (Figure 4f). The other DBP units, not participating in the stacking interactions with the guests, remain planar.

Interestingly, the cages do not bind the reduced form of **AQ**, 9,10-dihydroxyanthracene (**AQ-H**_**2**_; see Figures S84 and S85). This is likely because it is more electron-rich than **AQ** and, therefore, its stacking interactions with the DBP walls are less favorable. Notably, **AQ-H**_**2**_ is air-sensitive; simply bubbling air through its mixture with the cages for 2 min leads to oxidation and formation of **AQ**, which is readily encapsulated by both cages (see Figures S85 and S87).

Additionally, both cages show an affinity to testosterone and cholesterol and undergo desymmetrization, which is followed by splitting of the imine peak in **4Fe** and increasing the number of imine peaks in **4Zn** (Figure 4b,c), in analogy to similar examples in the literature.^50,51,53^

Finally, we tested 5,10-dimethyldibenzo­[*a*,*e*]­pentalene as a potential antiaromatic guest, but we found no evidence of binding (Figures S100 and S101).^29^

The cages, therefore, show a wide spectrum of binding behavior, depending on the added guest and the coordinated metal. Comparing their host-guest binding properties with related cages bearing anthracene units shows an impact of the offset of the anthracene substitution with the phenylene linkers (Figure S102).^50^ This offset in the DBP cages is somewhere in between that in the 1,5- and 9,10- anthracene analogues, and, indeed, we observe the impact of the differences in this offset on the binding abilities: the 1,5-anthracene Fe­(II) cage binds both C_60_ and C_70_, whereas the 9,10-anthracene cage binds none, while **4Fe** binds only C_60_. A similar impact of the substituents’ offset can be seen for cholesterol (**4Fe** binds it, similar to the 1,5-anthracene cage, and unlike the 9,10-anthracene cage) and pyrene (**4Fe** binds it in slow exchange similar to the 1,5-anthracene cage and unlike the 9,10-anthracene cage, which binds it in fast exchange). Therefore, one can conclude that the cages with antiaromatic walls can be as good as those with typical aromatic walls when it comes to qualitative guest binding.

Curiously, when comparing the chemical shift of the guests’ signals in the presence and absence of the cage, these are shifted upfield, which is opposite to what is expected from interacting with walls incorporating the antiaromatic pentalene motif. To compare, in the case of a norcorrole-based cage, the guests exhibited large paramagnetic shifts up to 24 ppm.^33^ These differences are likely caused by the following: (a) DBP is less antiaromatic than norcorrole (see the calculated magnetic shielding values, Figures 5 and S114); (b) the magnetic shielding effects rapidly diminish with increasing distance from the molecule plane;^25,26,54^ and (c) the pentalene unit is in the central part of the DBP core, whereas the guests’ protons are closer to the Clar sextets of the DBP and, therefore, they mostly experience a diamagnetic ring current, which causes relatively higher absolute NICS_(iso)_ values for points in the direction perpendicular to the ring (Figure 5) at distances larger than ca. 1.5 Å. The proximity of the guest’s protons to the phenylene rings of the DBP units is clearly visible in the crystal structure of **4Fe·(AQ)**_**2**_, as depicted in Figure 4e,f.

**Figure 5:**
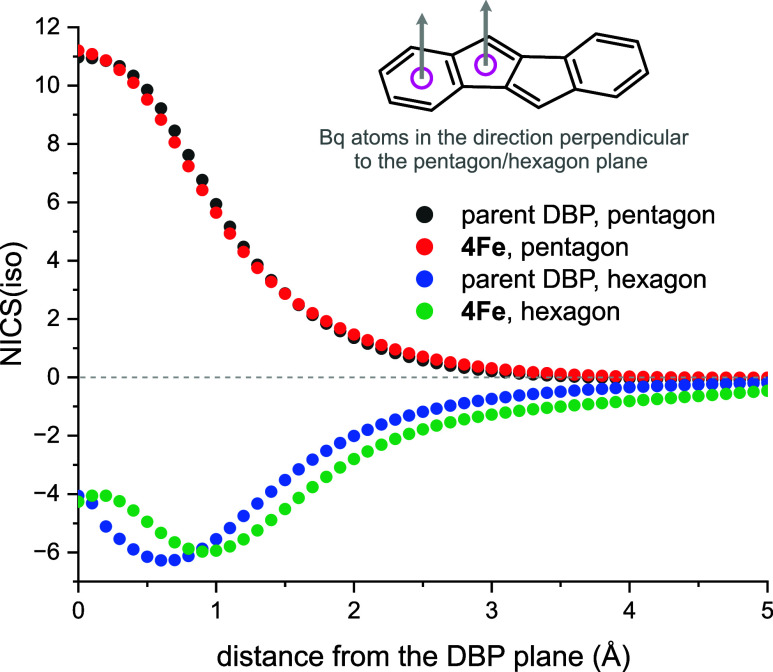
Comparison of the NICS­(iso) values obtained from DFT calculations, calculated in the direction perpendicular to the pentagon or hexagon of the DBP motif in both parent DBP and cage **4Fe**. The level of theory employed in the calculations is the same as for the previously reported norcorrole cage (B3LYP/SDD for Fe and 6-31G­(d) for C, H, and N).^38^

## 3 Conclusions

The coordination cages **4Fe** and **4Zn**, which incorporate benzannulation-stabilized antiaromatic pentalene motifs into their walls, constitute only the second example of coordination cages with antiaromatic panels. This approach successfully overcomes the inherent challenges in constructing antiaromatic supramolecular architectures by leveraging the stabilizing effect of benzannulation on the pentalene core. Both cages encapsulate fullerenes, with **4Zn** exhibiting superior adaptability over the less labile **4Fe**: it accommodates C_70_ in addition to C_60_ and undergoes pronounced structural reconfiguration upon C_60_ inclusion, while also showing broader affinity for PAH derivatives. Additionally, the cages selectively bind anthraquinone over its reduced form, 9,10-dihydroxyanthracene, and they bind selected steroids. While both cages bind smaller aromatic guests such as pyrene or anthraquinone, **4Fe** consistently forms complexes in the slow-exchange regime on the NMR time scale. A few of these inclusion complexes were unambiguously characterized by single-crystal X-ray diffraction. Despite the formally antiaromatic character of the pentalene motif embedded in the cage walls, the guests do not experience paratropic ring-current effect; instead, they are predominantly affected by the diatropic ring current of the Clar sextets of the benzannulated framework. This behavior demonstrates that the incorporation of antiaromatic motifs does not inevitably lead to paramagnetic deshielding of bound guests, provided the antiaromatic fragment is appropriately modified—here through benzannulation. Benzannulated antiaromatic motifs can be, therefore, incorporated into cages without interrupting conventional host–guest behavior, while simultaneously allowing systematic tuning of magnetic shielding effects. Overall, this work expands the still-limited collection of supramolecular assemblies featuring antiaromatic building blocks and is expected to stimulate greater interest in involving antiaromatic molecules in the design of functional materials.

## Supplementary Material



## Notes

NMR and MS spectra, computational results, additional figures, and single-crystal X-ray data collection and refinement details (PDF)

## Notes

We dedicate this work in memory of the late Prof. Erich Kleinpeter (1946–2025), a physical organic chemist widely known for his investigations into (anti)­aromaticity effects in molecules, including dibenzo­[*a*,*e*]­pentalenes.^25,26^

## Abbreviations

DBP: dibenzo­[*a*,*e*]­pentalene
NMR: nuclear magnetic resonance
MS: mass spectrometry
ESI: electrospray ionization
NOESY: Nuclear Overhauser Effect Spectroscopy
ROESY: Rotating Frame Overhauser Effect Spectroscopy
PAH: polycyclic aromatic hydrocarbon
