# Supplementary material for: Tetrahedral M II 4L6 (M = Fe, Zn) Cages Equipped with Walls Featuring Antiaromatic Pentalene Motifs
Source: JACS Au. 2026 Jul 15;6(7):4221–9. doi: 10.1021/jacsau.6c00720 (PMC13417229; doi:10.1021/jacsau.6c00720)
Supplement: Supplementary file 1 [file au6c00720_si_001.pdf]

Supporting Information

Tetrahedral $M^{II}_4L_6$ (M = Fe, Zn) cages equipped with walls featuring antiaromatic pentalene motif

Wojciech Stawski*[ab] and Birgit Esser*[ab]

a. Institute of Organic Chemistry II and Advanced Materials, Ulm University, Albert-Einstein-Allee 11, 89081 Ulm, Germany. Email: birgit.esser@uni-ulm.de; w.stawski97@gmail.com

b. CELEST Green Energy Lab Ulm, Ulm University, Lise-Meitner-Str. 16, 89081 Ulm, Germany.

Table of Contents

1. General methods S2

2. Synthesis and cage self-assembly S2

3. NMR and MS spectra S6

4. Guest binding S18

5. X-ray crystallography S67

6. UV-Vis spectroscopy S76

8. Theoretical calculations S77

9. References S88

1. General Methods

Solvents were purchased from Fisher Scientific and used without further purification. Iron(II) triflimide was synthesized as described in literature.[1] Zinc(II) triflimide was purchased from TCI. Cesium carborane was purchased from abcr. Other reagents and guests were purchased from Sigma Aldrich. NMR spectra were recorded on Bruker Avance Neo 400 or Bruker Avance Neo 600 spectrometers. NMR spectra were referenced against the residual solvent peak (CD_3CN: δ_H = 1.96 ppm, δ_C = 1.79 ppm; DMSO-d_6: δ_H = 2.50 ppm, δ_C = 39.51 ppm). Mass spectrometry measurements were performed using ESI ionization on an Agilent qTOF instrument in acetonitrile as solvent, gas temperature was 200 °C. UV-Vis absorption spectra were measured on a SHIMADZU UV-3600i Plus UV-VIS-NIR spectrophotometer using quartz cuvettes (1 cm path length). Signals in the NMR spectra were assigned based on correlations observed in COSY (COrrelation SpectroscopY), NOESY (Nuclear Overhauser Effect SpectroscopY), ROESY (Rotating-frame Overhauser Effect Spectroscopy) and HMBC (Heteronuclear Multiple Bond Correlation) experiments.

2. Synthesis and cage assembly

Imine **1** was synthesized as described in the literature.[2]

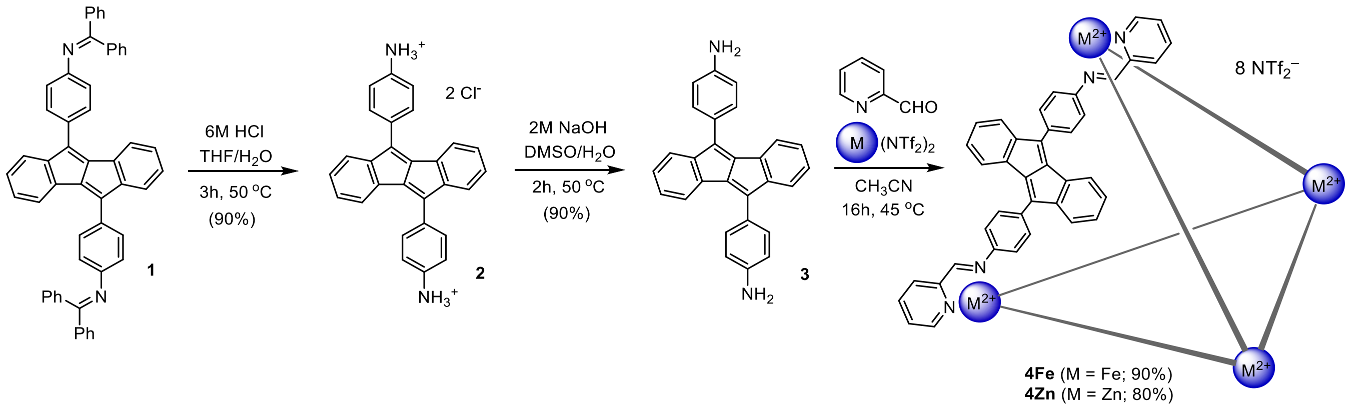

Synthesis of 2

Imine **1** (200 mg, 0.28 mmol) was suspended in a mixture of 6 M $HCl_{(aq)}$ (25 mL) and THF (50 mL) and vigorously stirred at 50 °C for 3 h. After that, most of the THF was removed using a rotary evaporator, and the resulting precipitate was filtered on a Büchner filter and washed with demineralized water (100 mL) and *n*-hexane (30 mL). The obtained yellow/orange precipitate of **2** was thoroughly dried in vacuo at 60 °C for 4 h. Yield: 116 mg (90%).

^{1}H NMR (600 MHz, DMSO-d_6) δ: 8.76 (broad, $-NH_3^+$), 7.64 (d, *J* = 8.1 Hz, 4H), 7.27 (d, *J* = 8.1 Hz, 4H), 7.17 (d, *J* = 7.5 Hz, 2H), 7.04 (d, *J* = 7.5 Hz, 2H), 7.01–6.90 (overlapping m, 4H).

^{13}C NMR (151 MHz, DMSO-d_6) δ: 148.4, 141.2, 139.5, 134.5, 129.7, 127.9, 127.8, 122.6, 121.41, 119.9. Two of the ^{13}C signals are not visible likely due to overlapping and/or broadening.

ESI MS: not detected

Synthesis of 3

Hydrochloride **2** (80 mg, 0.175 mmol) was suspended DMSO (8 mL) at 50 °C, and an aqueous solution of NaOH was added (2 M, 10 mL) over 2 min. The mixture was vigorously stirred for 2 h, after which an extraction with dichloromethane (5 × 50 mL) was performed. The combined organic phases were washed with H_2O three times, and then with brine (1×). The solvent was evaporated, and the obtained solid was washed with *n*-hexane and dried, obtaining brown crystalline solid. Yield: 60.5 mg (90%).

^{1}H NMR (400 MHz, DMSO-d_6) δ: 7.38 (d, *J* = 8.5 Hz, 4H), 7.28–7.21 (m, 2H), 7.13–7.06 (m, 2H), 6.96–6.86 (m, 4H), 6.74 (d, *J* = 8.5 Hz, 4H), 5.64 (s, 4H).

^{13}C NMR (101 MHz, DMSO-d_6) δ: 150.4, 149.0, 140.3, 139.8, 135.8, 130.1, 127.7, 127.5, 123.0, 121.5, 120.5, 114.1.

ESI MS: not detected

X-ray crystallography confirmed the structure (see Figure S103).

Assembly of 4Fe

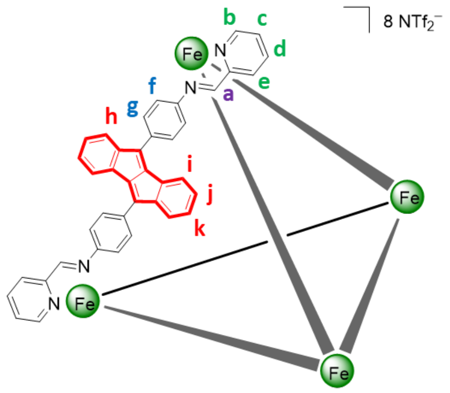

Bis-aniline **3** (25mg, 0.065 mmol), pyridine 2-carboxaldehyde (14 mg, 0.131 mmol, 2 equiv.) and iron(II) triflimide (26.75 mg, 0.043 mmol, 0.67 equiv.) were dissolved in acetonitrile (12.5 mL), and the solution was flushed with argon. The resulting red-brown solution was stirred at 45 °C for 16 h, after which it was concentrated in vacuo to ca. 1.0 mL. Diethyl ether (10 mL) was added, and the mixture was centrifuged. The supernatant was discarded, the brown-red solid was re-dissolved in fresh acetonitrile (1 mL), and the cage was precipitated with diethyl ether again (2×). The isolated solid was dried in high vacuum at 50 °C, which provided cage **4Fe** as a brown-red solid. Yield: 57.0 mg (90%).

^{1}H NMR (600 MHz, CD_3CN) δ 9.05 (imine **a**, s, 12H), 8.62 (pyridine **e**, d, *J* = 7.5 Hz, 12H), 8.43 (pyridine **d**, m, 12H), 7.81 (pyridine **c**, m, 12H), 7.43 (pyridine **b**, d, *J* = 5.5 Hz, 12H), 7.34 (phenylene **g**, d, *J* = 8.2 Hz, 24H), 6.98 (DBP **h**/**i**/**j**/**k**, m, 12H), 6.88 (DBP **h**/**i**/**j**/**k**, m, 12H), 5.53 (phenylene **f**, broad d, 24H).

^{13}C NMR (151 MHz, CD_3CN) δ 176.7, 159.6, 157.5, 152.0, 150.9, 144.8, 141.2, 141.1, 135.8, 135.2, 133.1, 131.5, 131.0, 130.1, 129.6, 123.4, 122.9. One of the signals is missing, probably due to overlapping.

^{19}F NMR (545 MHz, CD_3CN) δ –80.15

HRMS (ESI): observed m/z = 449.8837, calcd. for $Fe_4C_{240}H_{156}N_{24}{}^{8+}$: 449.8793

X-ray crystallography confirmed the structure (see Figure S104).

Assembly of 4Zn

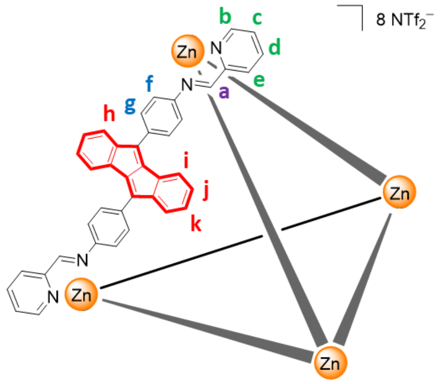

Bis-aniline **3** (25mg, 0.065 mmol), pyridine 2-carboxaldehyde (14 mg, 0.131 mmol, 2 equiv.) and zinc(II) triflimide (26.9 mg, 0.043 mmol, 0.67 equiv.) were dissolved in acetonitrile (12.5 mL). The resulting orange solution was stirred at 45 °C for 16 h, after which it was concentrated in vacuo to ca. 0.5 mL. Diethyl ether (15 mL) was added, and the mixture was centrifuged. The supernatant was discarded, the orange solid was re-dissolved in fresh acetonitrile (0.5 mL), and the cage was precipitated with diethyl ether again (2×). The isolated orange-brown solid was dried in high vacuum at 50 °C, which provided cage **4Zn**. Yield: 51.0 mg (80%).

^{1}H NMR (600 MHz, CD_3CN) δ 8.83–8.74 (imine **a**, 12H), 8.55–8.45 (pyridine **d**, d/m, 12H), 8.38–8.28 (pyridine **e**, d/m, 12H), 8.11–7.78 (pyridine **c**/**b**, d/m 24H), 7.79–7.45 (phenylene **f**/**g**, d, 24H), 7.26–6.36 (phenylene **f**/**g**, d and DBP **h**/**i**/**j**/**k**, m, 72H).

^{13}C NMR (151 MHz, CD_3CN) δ 166.65, 166.57, 166.4, 166.3, 166.1, 166.0, 165.7, 165.6, 151.1, 151.07, 150.98, 150.9, 150.8, 150.6, 150.5, 150.4, 150.3, 149.3, 149.2, 149.15, 149.07, 149.0, 148.81, 148.75, 148.6, 147.9, 147.79, 147.76, 147.74, 147.68, 144.9, 144.78, 144.76, 144.71, 144.69, 144.6, 144.3, 144.24, 144.18, 144.1, 144.04, 141.1, 141.0, 141.00, 140.98, 140.95, 135.85, 135.83, 135.80, 135.76, 135.72, 135.68, 135.12, 134.98, 134.84, 134.70, 134.59, 134.54, 132.9, 132.7, 132.4, 131.35, 131.3, 131.2, 131.07, 130.00, 129.9, 129.8, 129.7, 129.6, 129.40, 129.25, 124.21, 124.15, 124.00, 123.9, 123.8, 123.7, 123.6, 123.45, 123.39, 123.2.

^{19}F NMR (545 MHz, CD_3CN) δ –80.08

ESI MS: not detected, likely due to the labile character of Zn assemblies in comparison with Fe analogues

9,10-Dihydroxyanthracene (AQ-H_2)

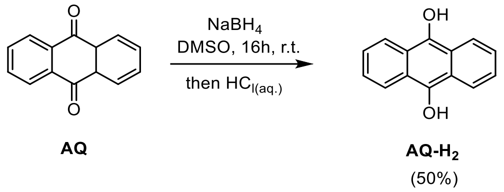

The compound was synthesized according to a modified literature procedure.[3]

Anthraquinone (AQ, 400 mg, 1.92 mmol) was placed in a flask containing dry, degassed (by bubbling with argon) DMSO (6 mL), and $NaBH_4$ (435 mg, 11.52 mmol, 6 equiv.) was added in one portion. The flask was closed, and the obtained mixture was vigorously stirred at room temperature for 16 h, which was followed by a color change to dark red. The solution was carefully, but quickly transferred to 2 M HCl (100 mL; hydrogen evolving!) and immediately filtered under reduced pressure (in air), and washed with water (50 mL) and dichloromethane (20 mL). The obtained green-yellow solid was immediately transferred to a flask and thoroughly dried at 80 °C for 6 h. At this point, the solid is a mixture of 9,10-dihydroxyanthracene (AQ-H_2) and anthraquinone (AQ), which was formed due to exposure to air during quenching with acid and filtration. To obtain pure 9,10-dihydroxyanthracene (AQ-H_2), the obtained solid was suspended in degassed dichloromethane under argon (20 mL), and filtration under argon over a Schlenk frit was performed, followed by a wash of the isolated precipitate with another portion of dichloromethane (20 ml). The Schlenk frit with the obtained solid was dried in vacuum at room temperature (6 h) and transferred to a glovebox. The product was isolated as a yellow solid. Yield: 50% (200 mg).

^{1}H NMR (400 MHz, CD_3CN) δ 8.31 (dd, *J* = 6.8, 3.3 Hz, 4H), 7.49 (dd, *J* = 6.8, 3.3 Hz, 4H), 7.10 (s, 2H).

^{13}C NMR (101 MHz, CD_3CN) δ 142.4, 125.8, 123.4, 122.0.

3. NMR and mass spectra

3.1. Bis-aniline hydrochloride 2

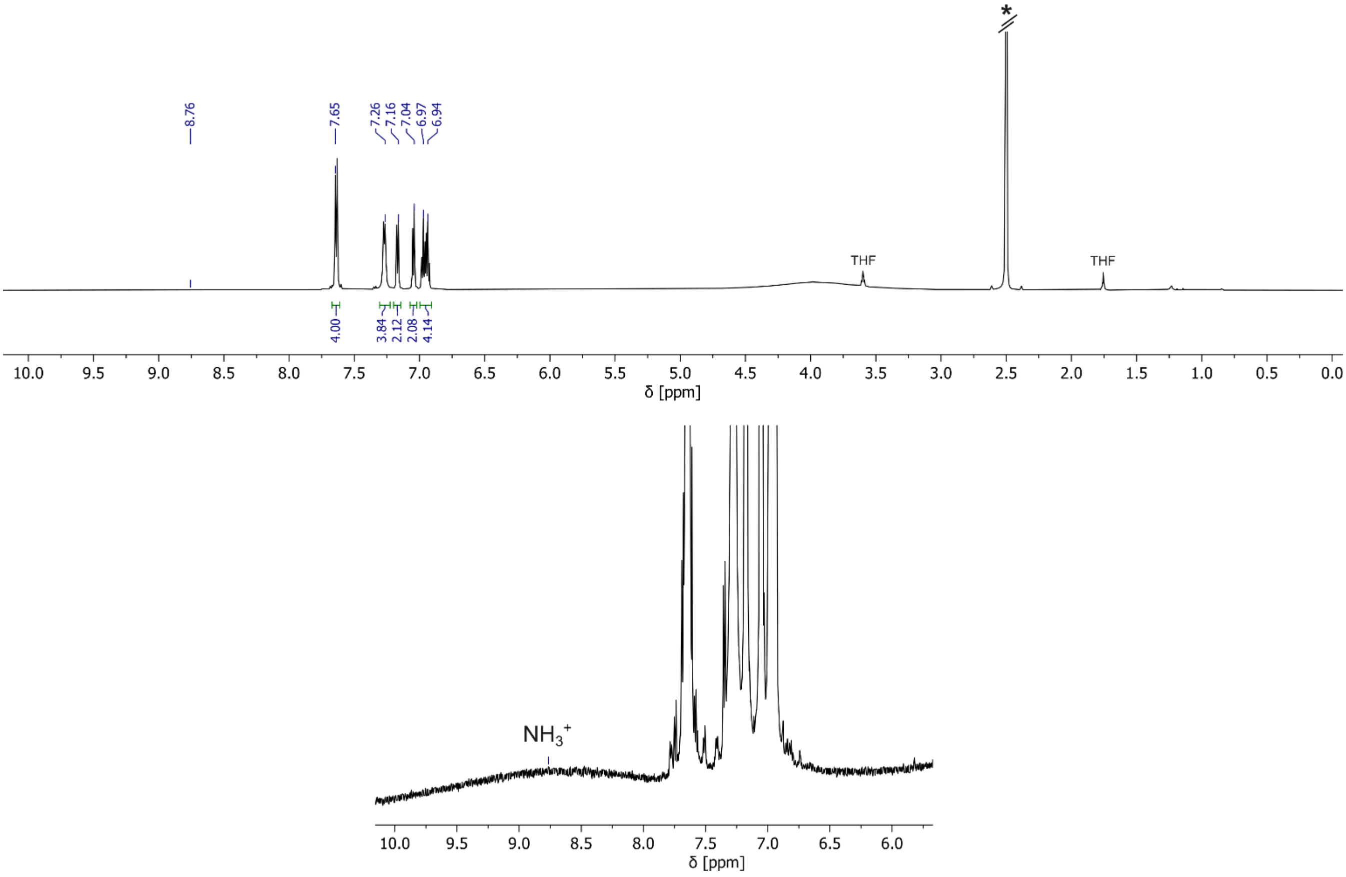

Figure S1. ^{1}H NMR spectrum of **2** (top) and zoom on the ammonium signal (bottom), DMSO-*d₆*, 298 K, 600 MHz.

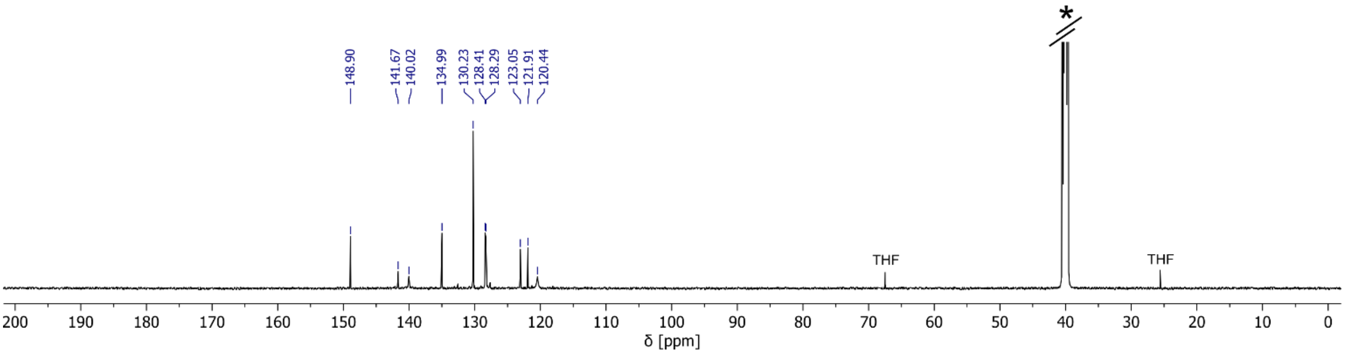

Figure S2. ^{13}C NMR spectrum of **2**, DMSO-d_6, 298 K, 151 MHz.

3.2. Bis-aniline 3

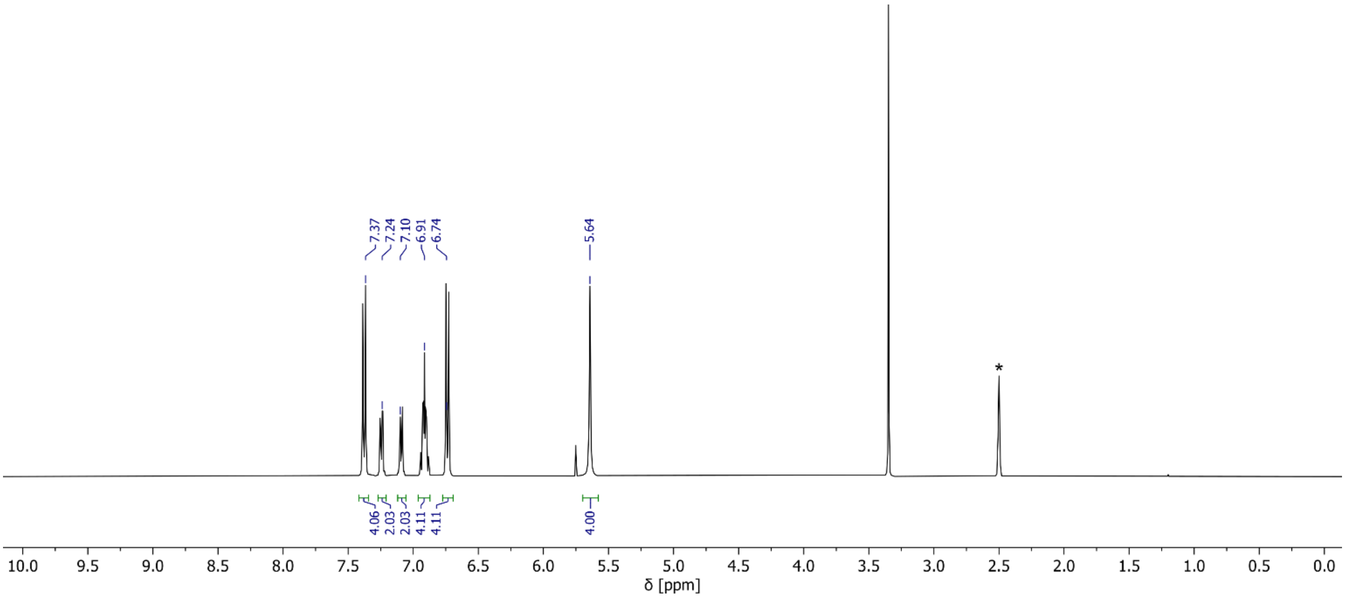

Figure S3. ^{1}H NMR spectrum of **3**, DMSO-d_6, 298 K, 400 MHz.

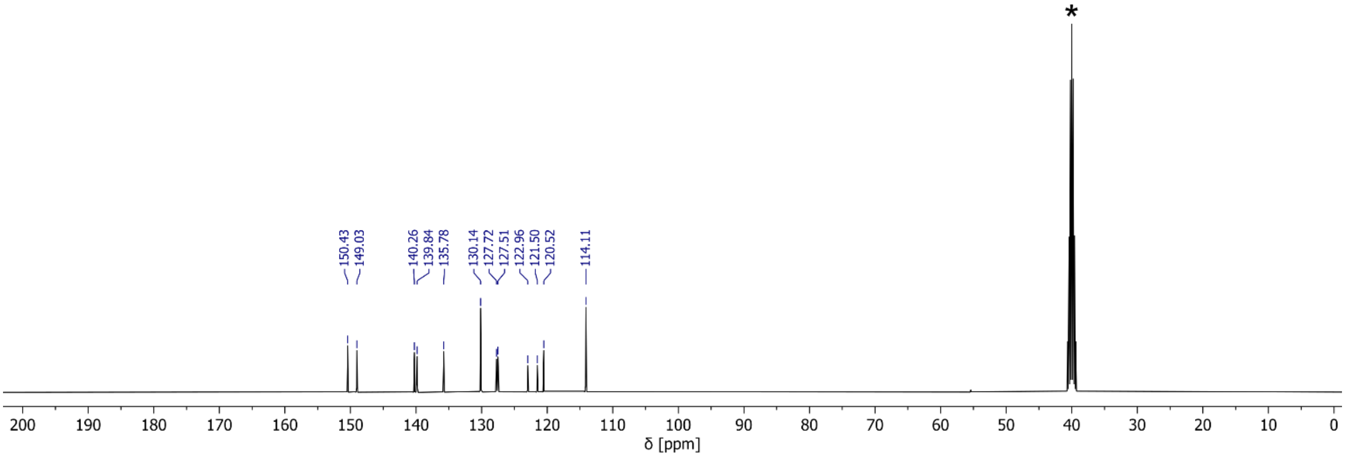

Figure S4. ^{13}C NMR spectrum of **3**, DMSO-d_6, 298 K, 101 MHz.

3.3. Cage 4Fe

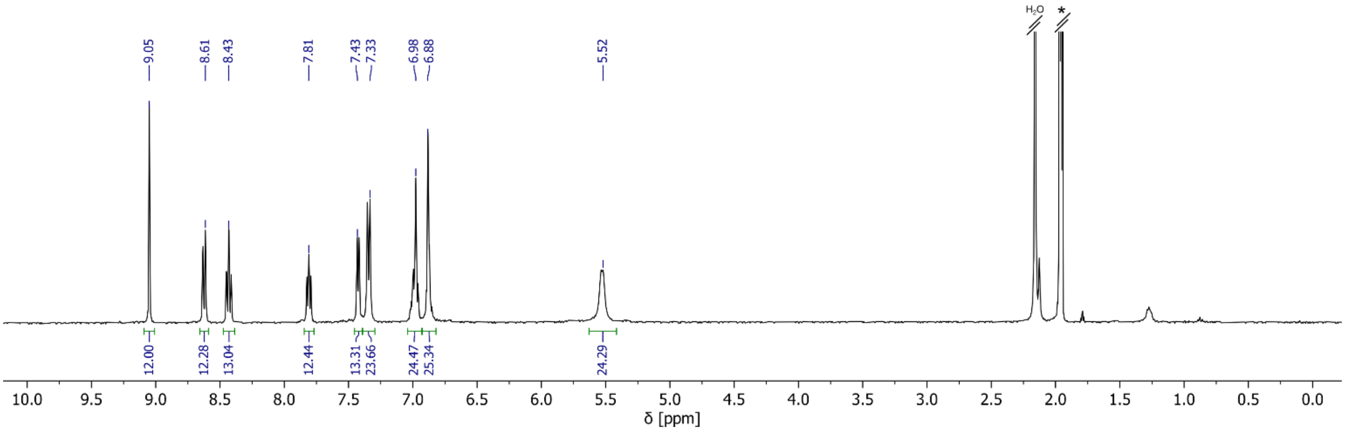

Figure S5. Full ^{1}H NMR spectrum of **4Fe**, CD_3CN, 298 K, 600 MHz.

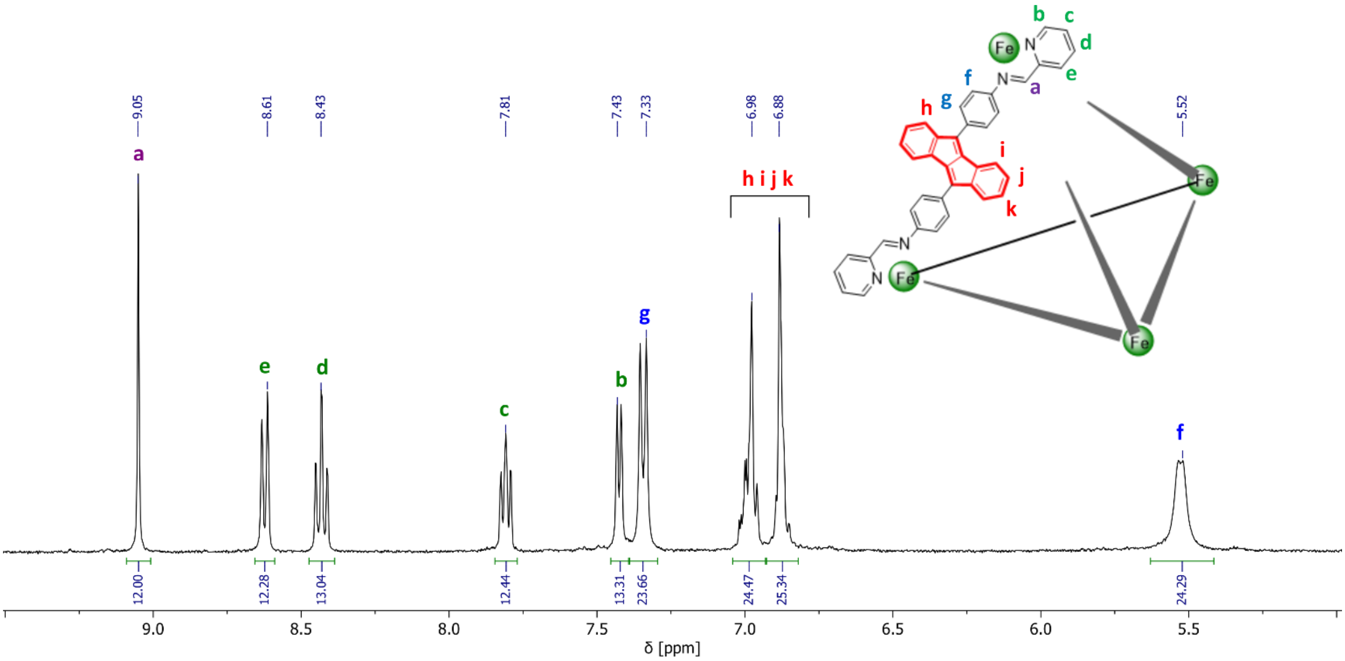

Figure S6. ^{1}H NMR spectrum of **4Fe**, CD_3CN, 298 K, 600 MHz. Zoom in of the aromatic region with partial signals assignment.

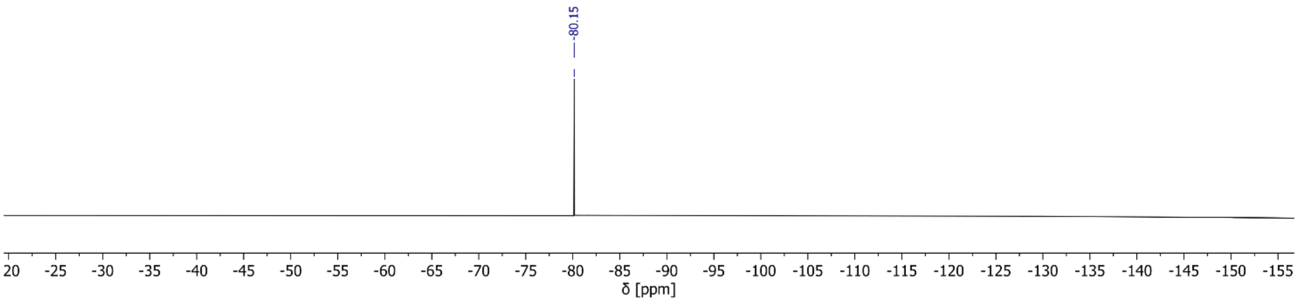

Figure S7. ^{19}F NMR spectrum of **4Fe**, CD_3CN, 298 K, 565 MHz.

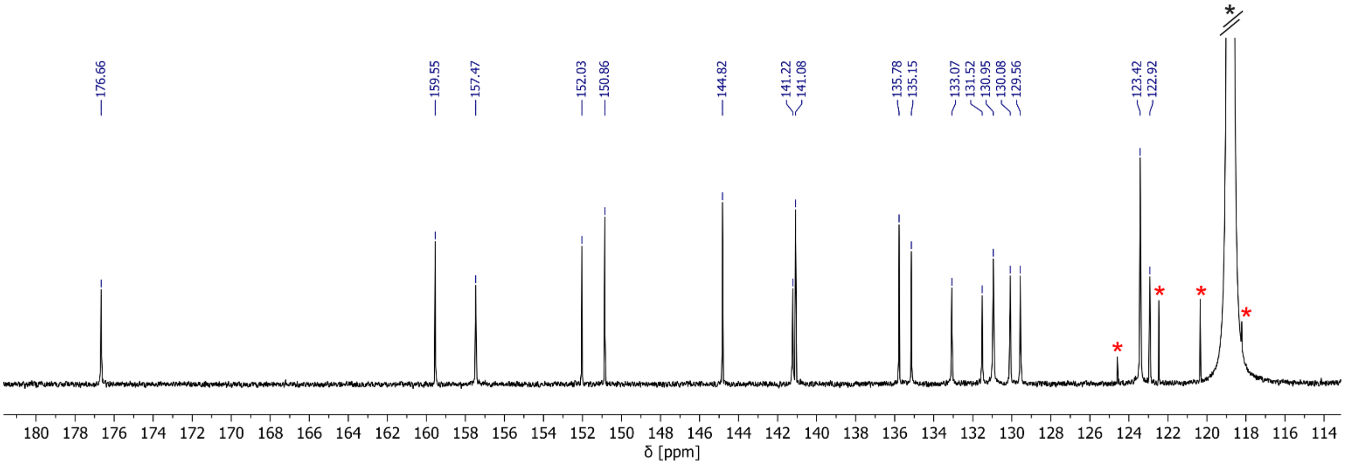

Figure S8. ^{13}C NMR spectrum of **4Fe**, CD_3CN, 298 K, 151 MHz. Zoom in of the aromatic region. Black asterisks indicate residual CD_3CN signal and red asterisks the triflimide signal (quartet due to coupling with fluorine).

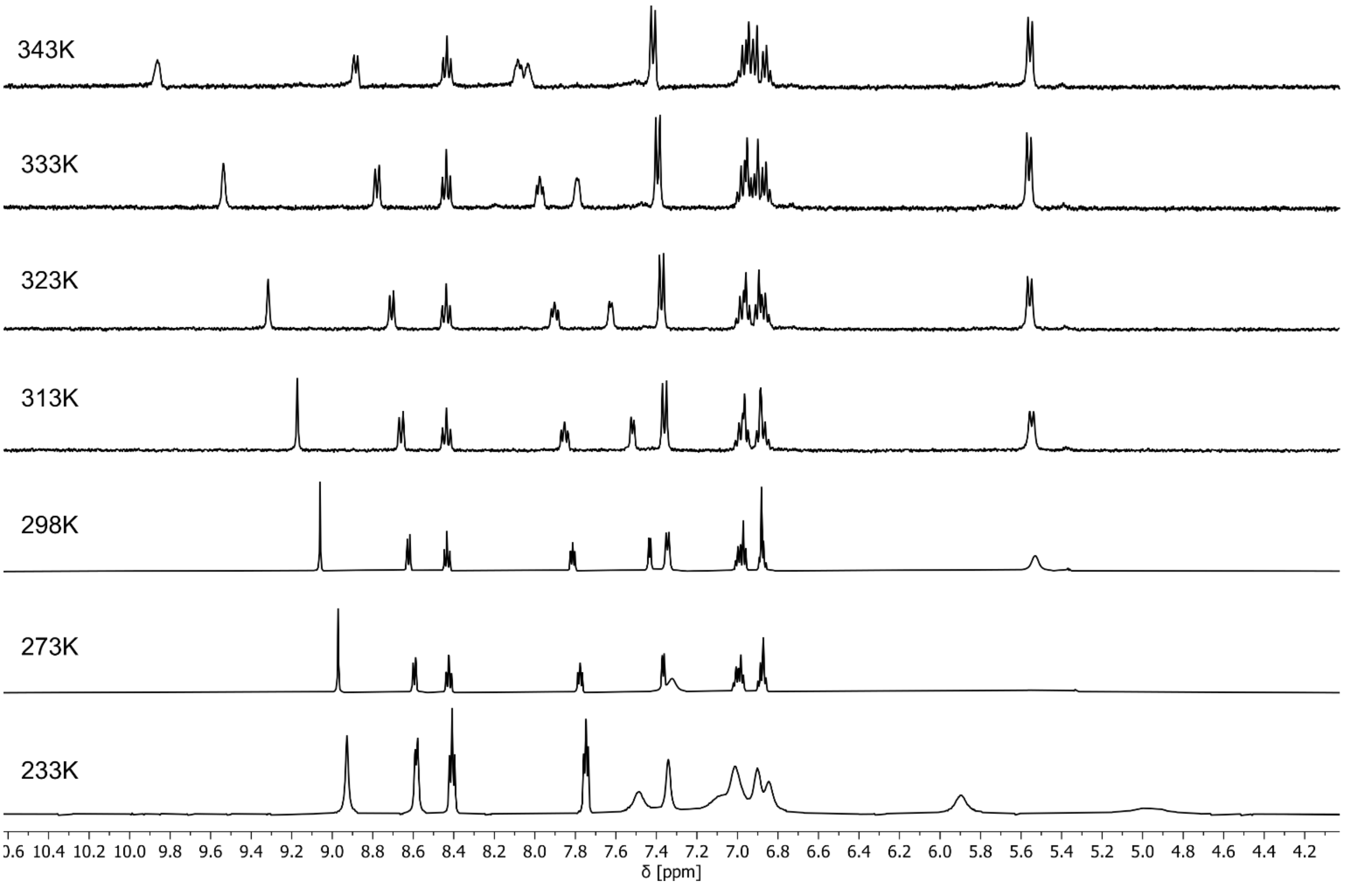

Figure S9. Variable-temperature ^{1}H NMR spectra of **4Fe**, CD_3CN, 600 MHz.

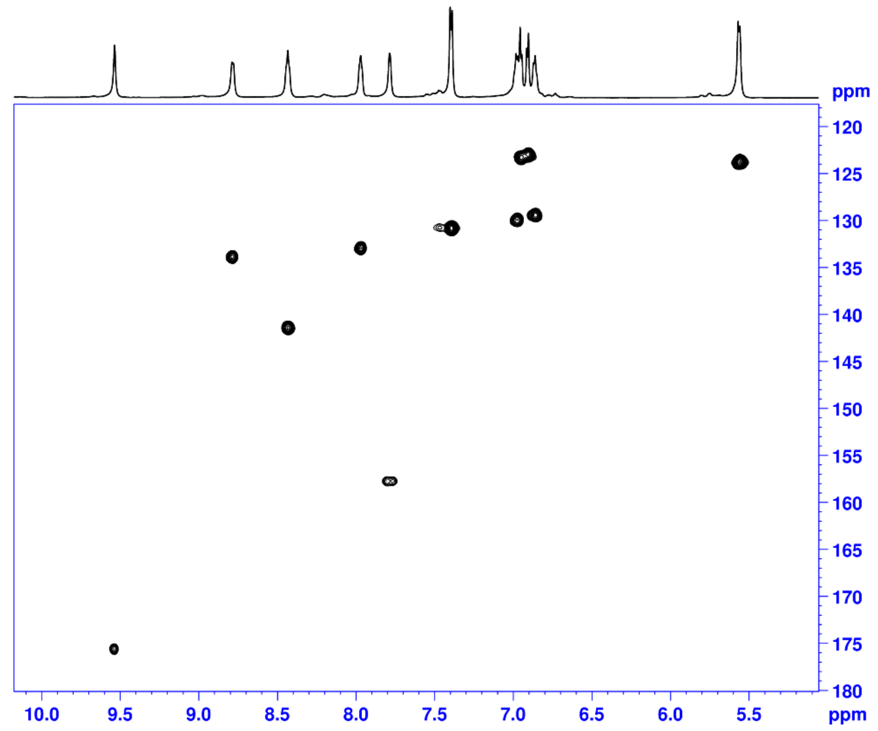

Figure S10. ^{1}H-^{13}C HSQC NMR spectrum of **4Fe**, CD_3CN, 333 K, 600 MHz. The DBP peaks became resolved at this temperature and four different HSQC correlations are visible.

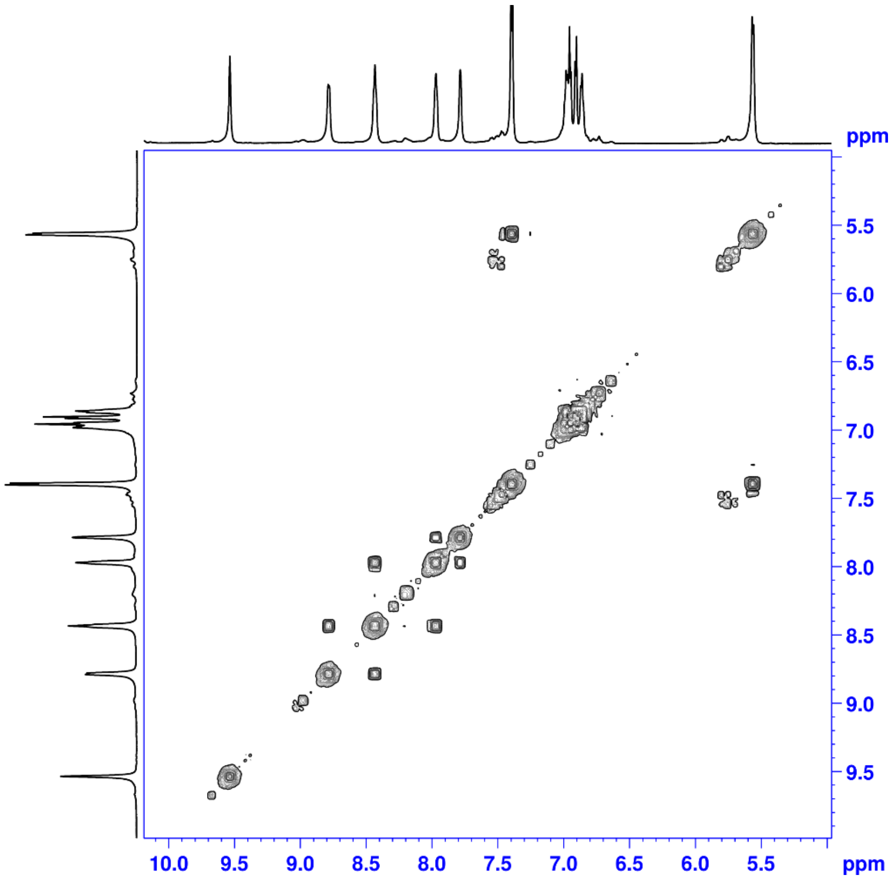

Figure S11. ^{1}H-^{1}H COSY NMR spectrum of **4Fe**, CD_3CN, 333 K, 600 MHz.

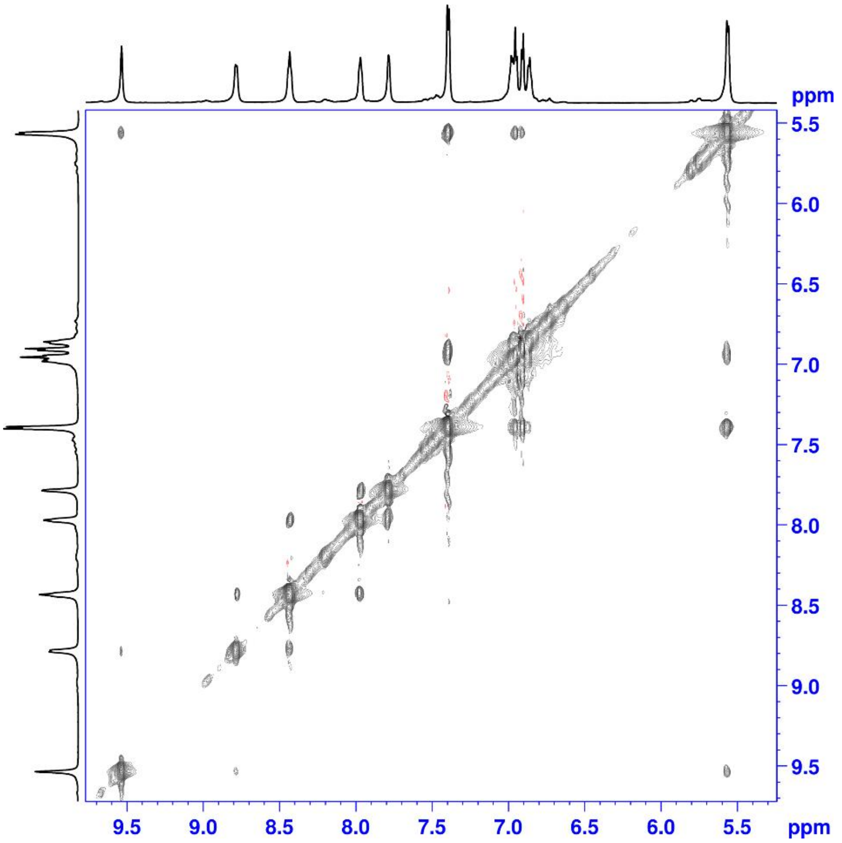

Figure S12. ^{1}H-^{1}H NOESY NMR spectrum of **4Fe**, CD_3CN, 333 K, 600 MHz.

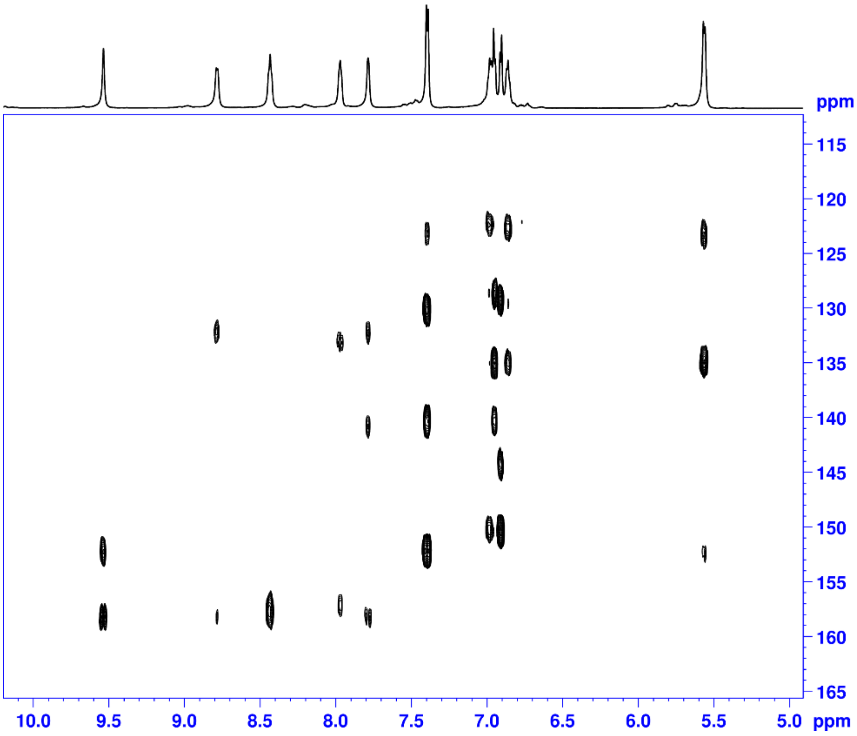

Figure S13. ^{1}H-^{13}C HMBC NMR spectrum of **4Fe**, CD_3CN, 333 K, 600 MHz.

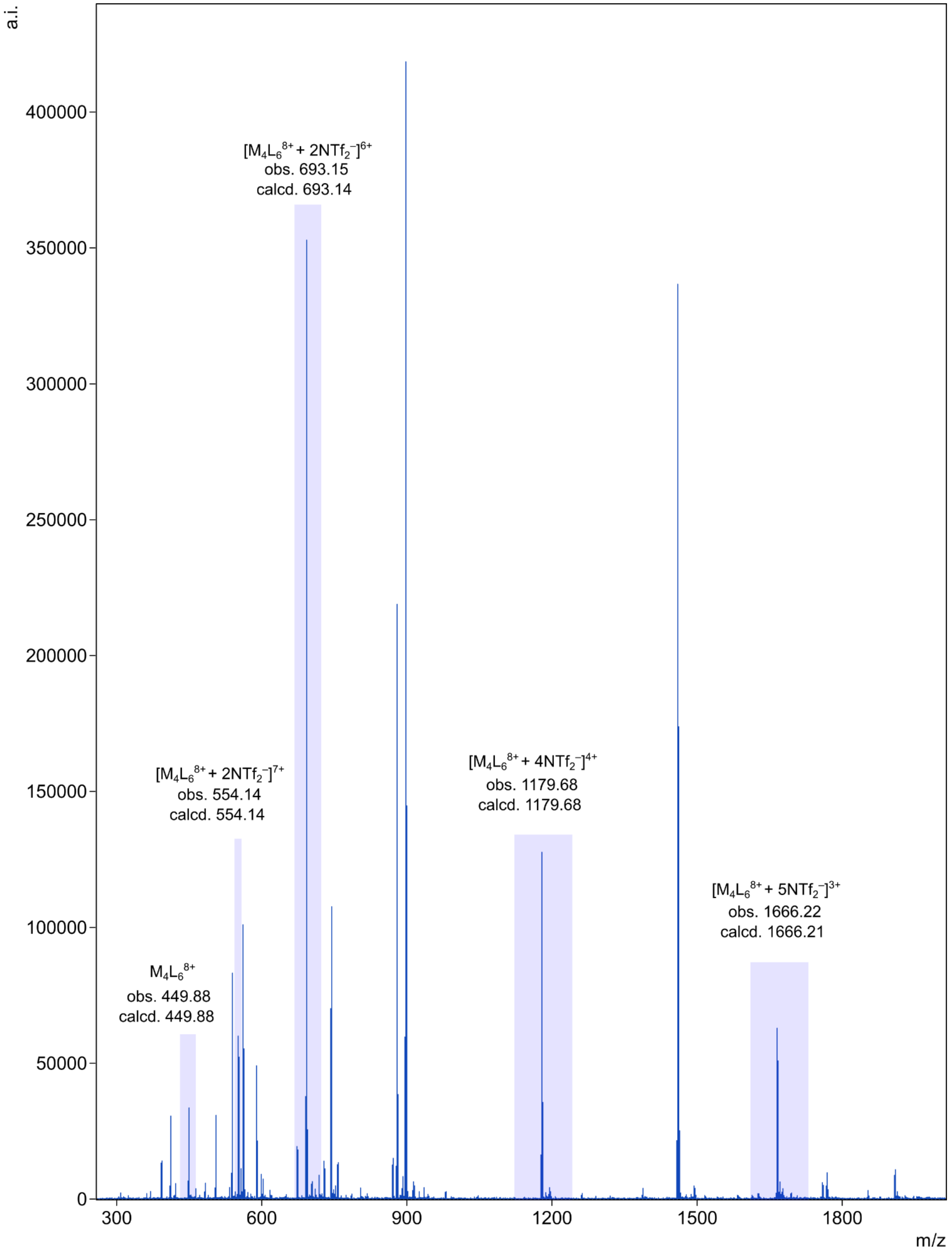

Figure S14. ESI-TOF mass spectrum of **4Fe**, view on the full spectrum.

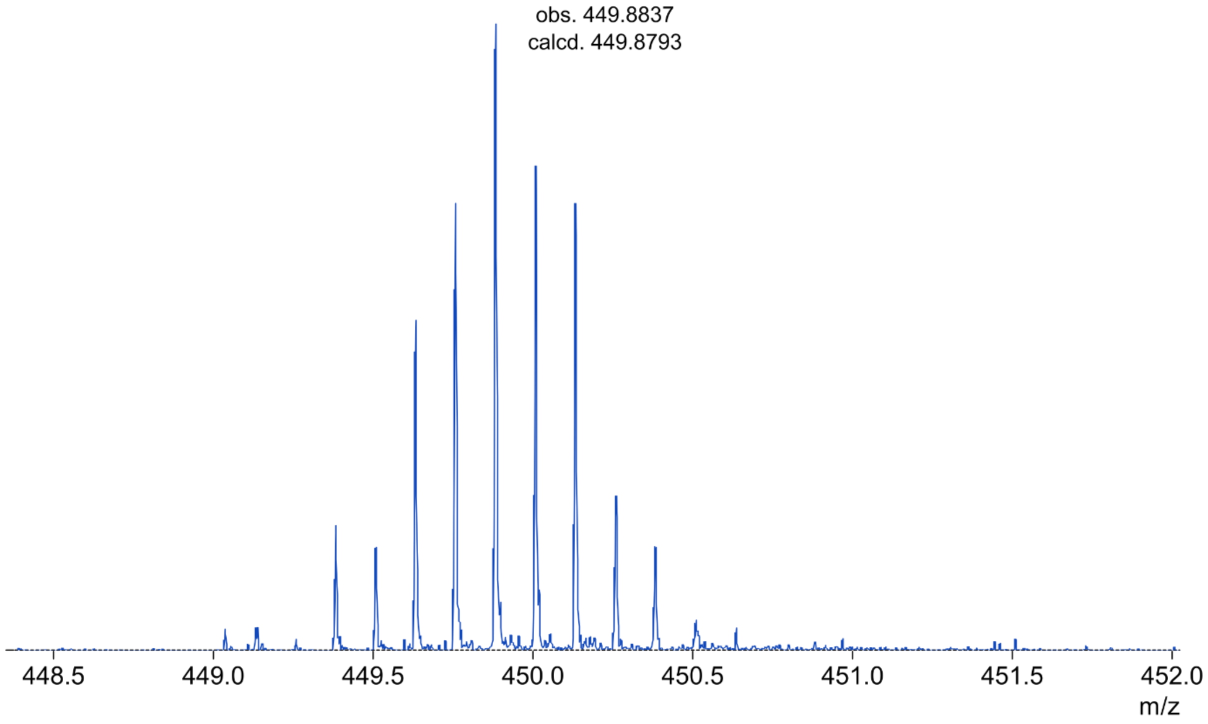

Figure S15. Zoom at the isotopic pattern of octacation of **4Fe**.

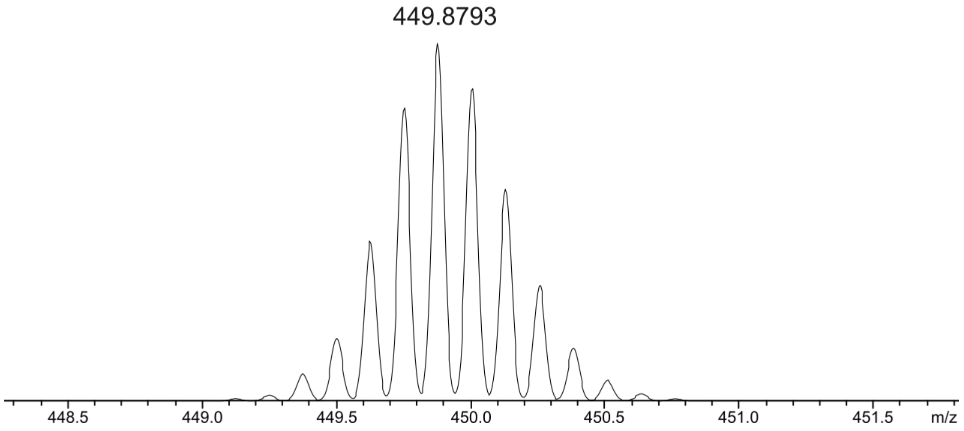

Figure S16. Simulated isotopic pattern of octacation of **4Fe**.

3.4. Cage 4Zn

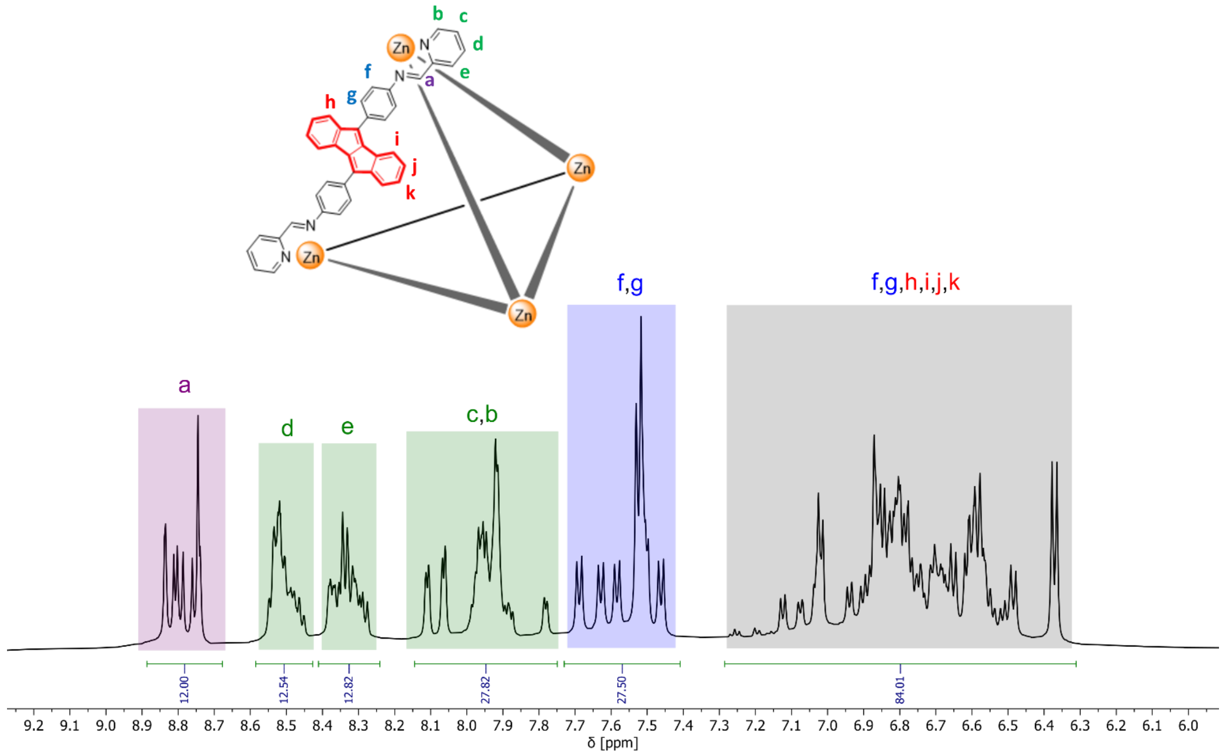

Figure S17. ^{1}H NMR spectrum of **4Zn**, CD_3CN, 298 K, 600 MHz.

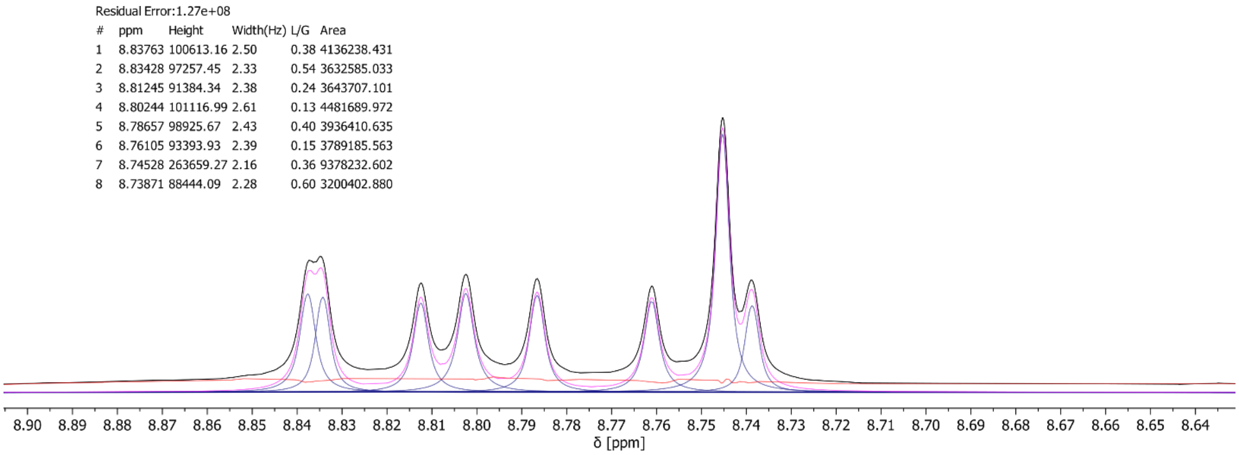

Figure S18. Deconvolution of the imine region, CD_3CN, 298 K, 600 MHz.

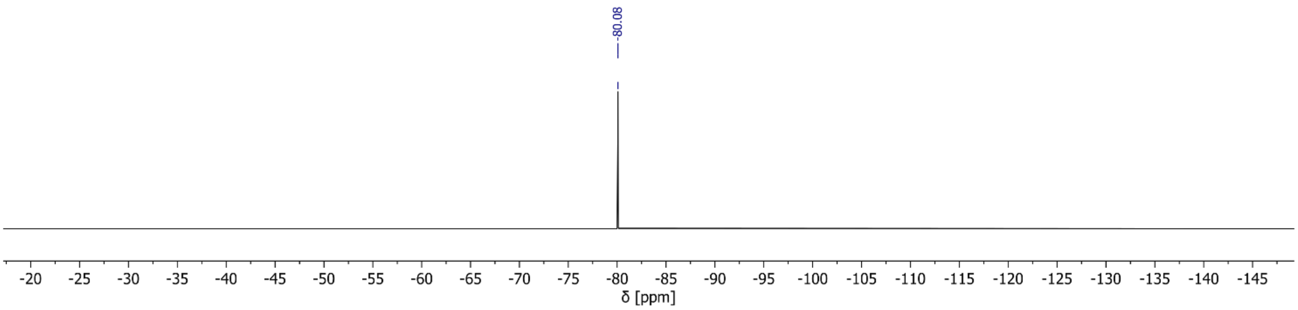

Figure S19. ^{19}F NMR spectrum of **4Zn**, CD_3CN, 298 K, 565 MHz.

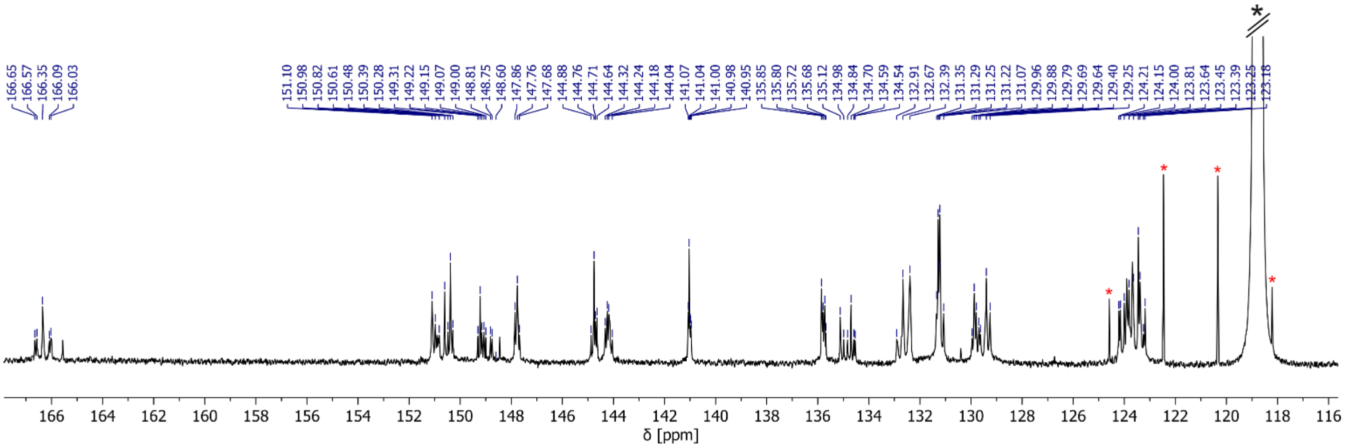

Figure S20. ^{13}C NMR spectrum of **4Zn**, CD_3CN, 298 K, 151 MHz. Black asterisk indicates residual CD_3CN signal and red asterisks triflimide signal (quartet due to coupling with fluorine).

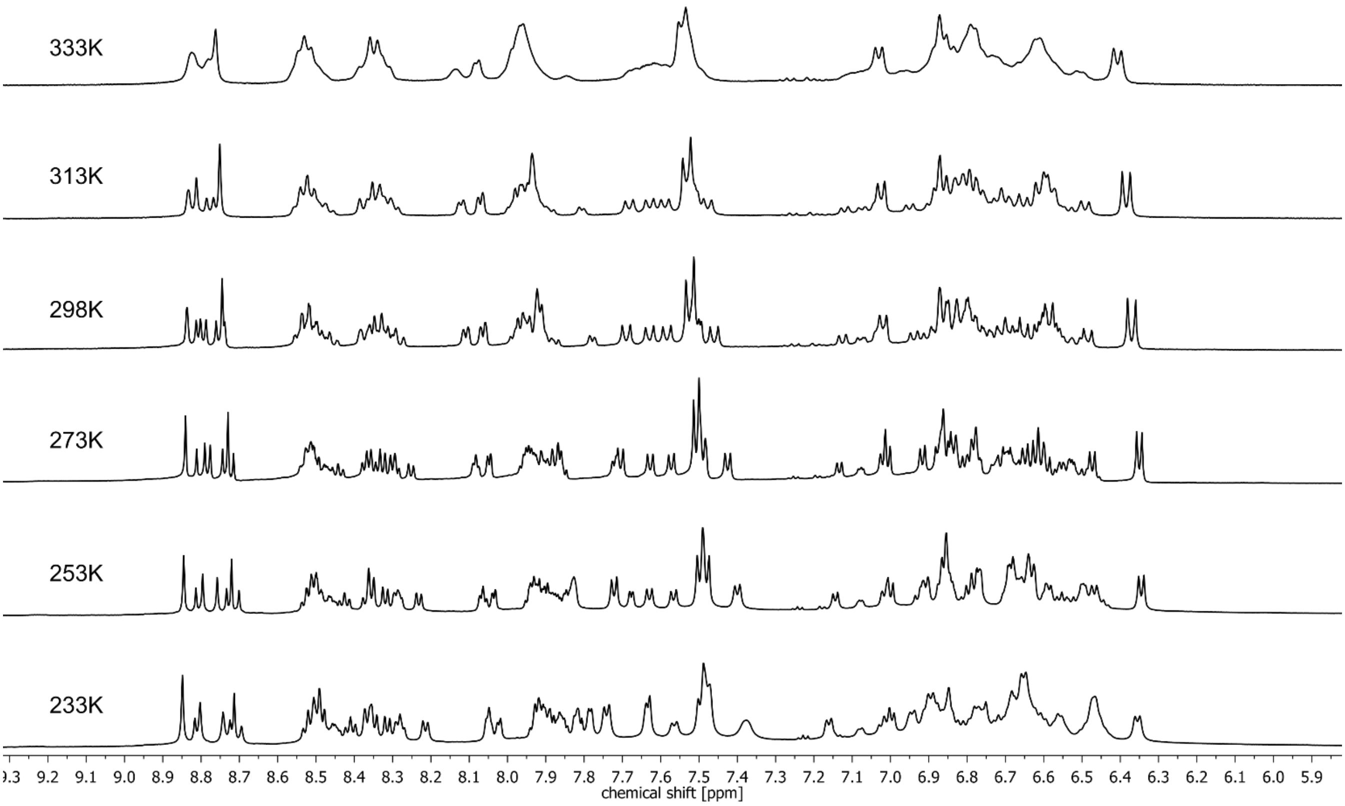

Figure S21. Variable-temperature ^{1}H NMR spectra of **4Zn**, CD_3CN, 600 MHz.

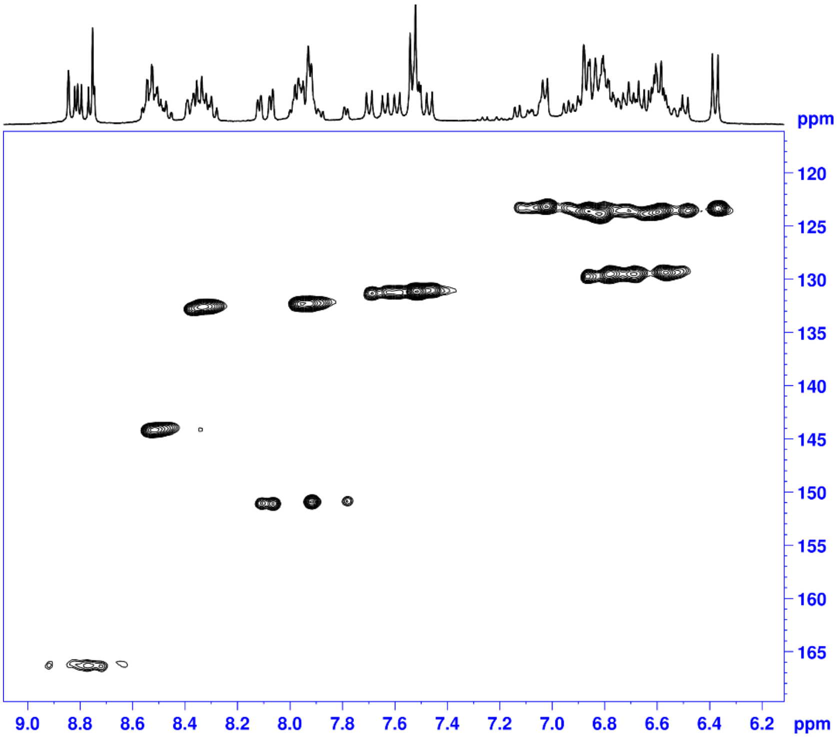

Figure S22. ^{1}H-^{13}C HSQC NMR spectrum of **4Zn**, CD_3CN, 298 K, 600 MHz.

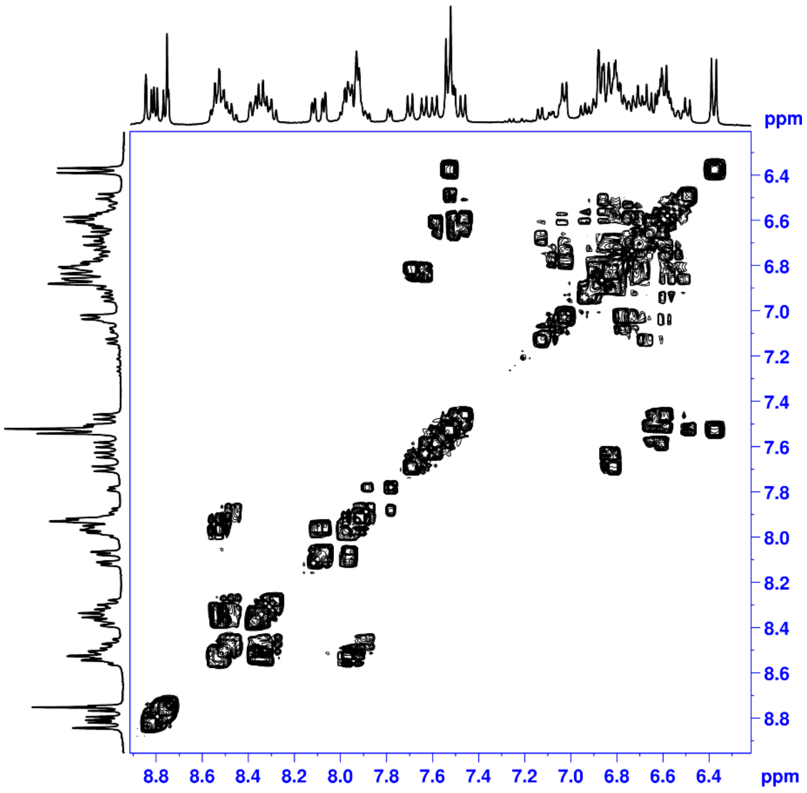

Figure S23. ^{1}H-^{1}H COSY NMR spectrum of **4Zn**, CD_3CN, 298 K, 600 MHz.

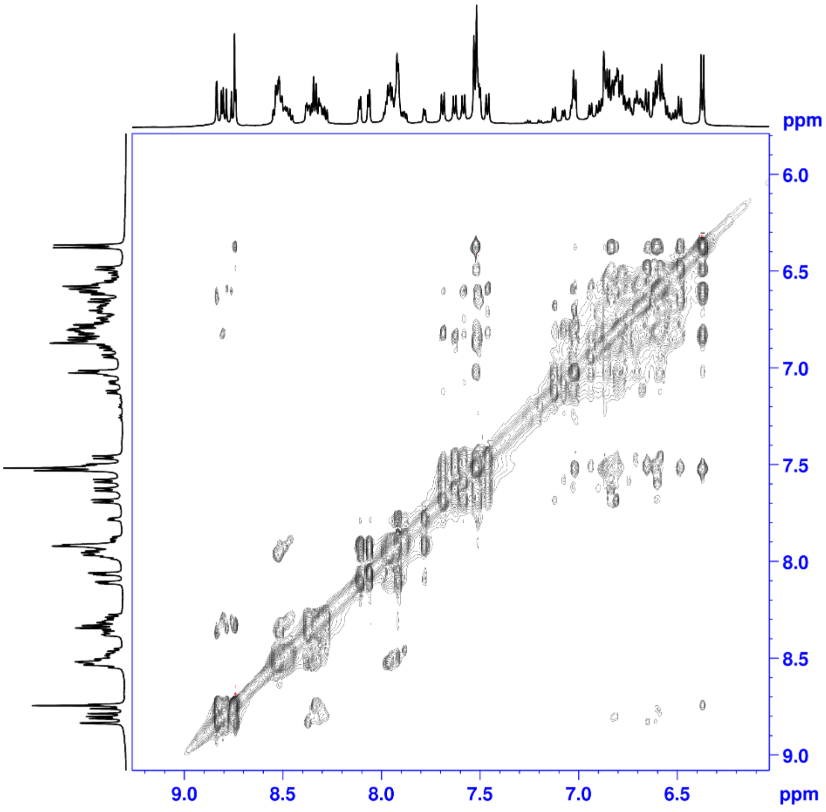

Figure S24. ^{1}H-^{1}H NOESY NMR spectrum of **4Zn**, CD_3CN, 298 K, 600 MHz.

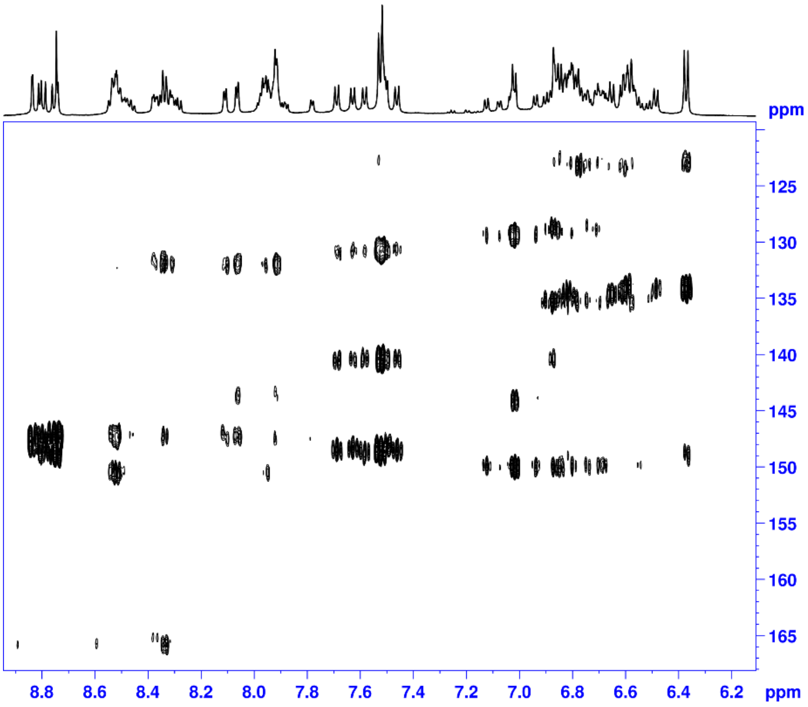

Figure S25. ^{1}H-^{13}C HMBC NMR spectrum of **4Zn**, CD_3CN, 298 K, 600 MHz.

3.5. 9,10-Dihydroxyanthracene (AQ-H$_2$)

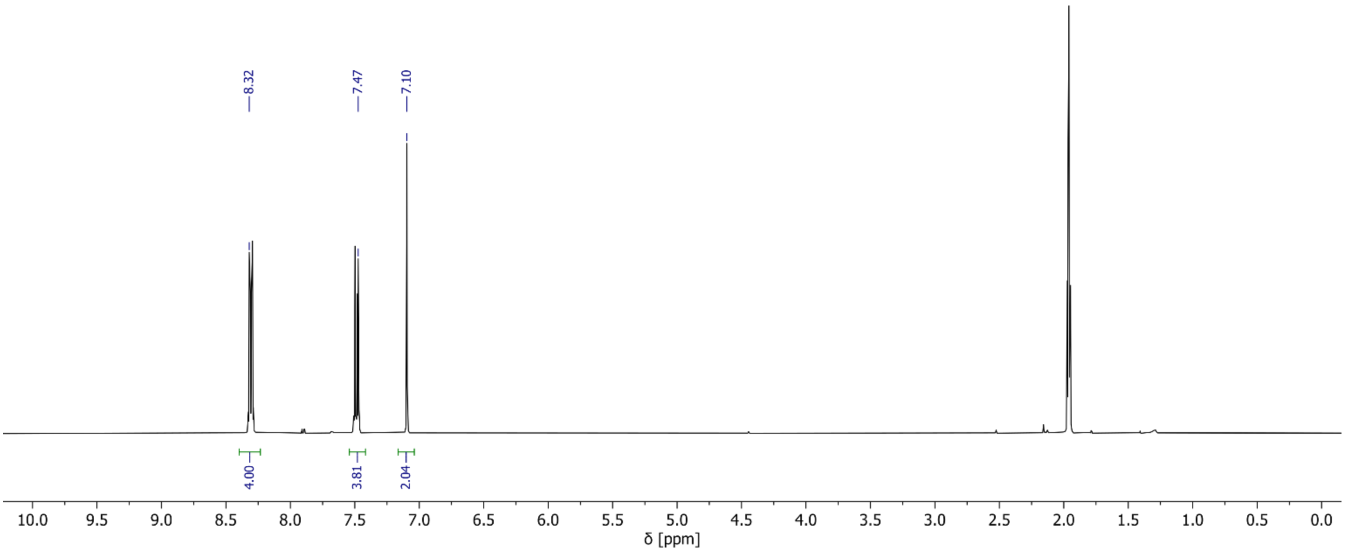

Figure S26. ^{1}H NMR spectrum of **AQ-H$_2$**, CD_3CN, 298 K, 400 MHz. The NMR sample was prepared in an Ar-filled glovebox in a J-Young tube due to the air-sensitive nature of the sample (oxidation to anthraquinone by air).

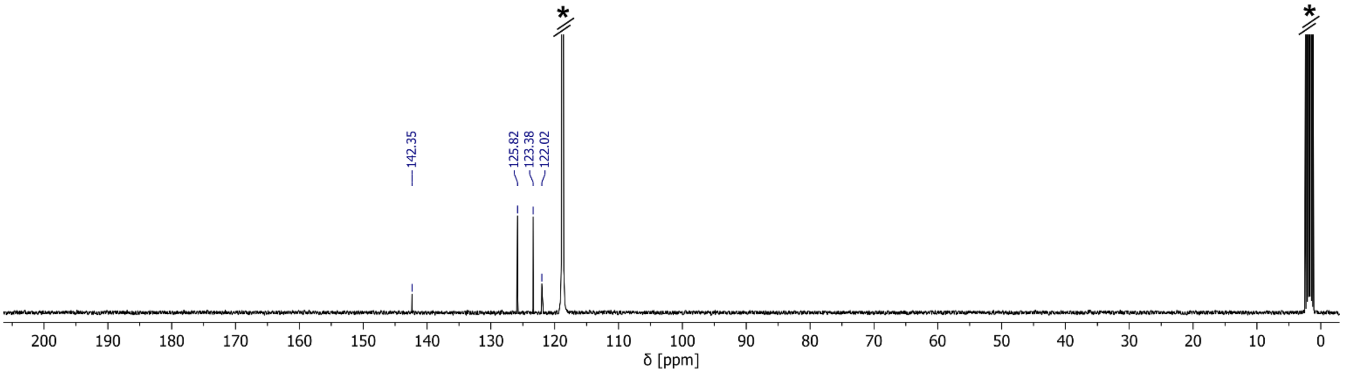

Figure S27. ^{13}C NMR spectrum of **AQ-H$_2$**, CD_3CN, 298 K, 101 MHz. The NMR sample was prepared in an Ar-filled glovebox in a J-Young tube due to the air-sensitive nature of the sample (oxidation to anthraquinone by air).

4. Guest binding

4.1. Fullerene binding

Solutions of cages **4Fe** and **4Zn** (ca. 1 mM) were placed in NMR tubes and excess of C_{60} or C_{70} fullerenes was added (ca. 10 equiv.). The NMR tubes were sonicated for 1 min and then rotated by attaching them to a rotary evaporator's joint to ensure sufficient mixing, and binding was monitored by ^{1}H NMR spectroscopy. When the reaction was not progressing or the progress was very slow at room temperature, the NMR tube was immersed in an oil bath preheated to 50 °C, or, if the reaction was too slow and to speed up the process, the mixture from the NMR tube was transferred to a glass vial and was vigorously stirred at 50 °C.

4.1.1. Binding of C_{60}

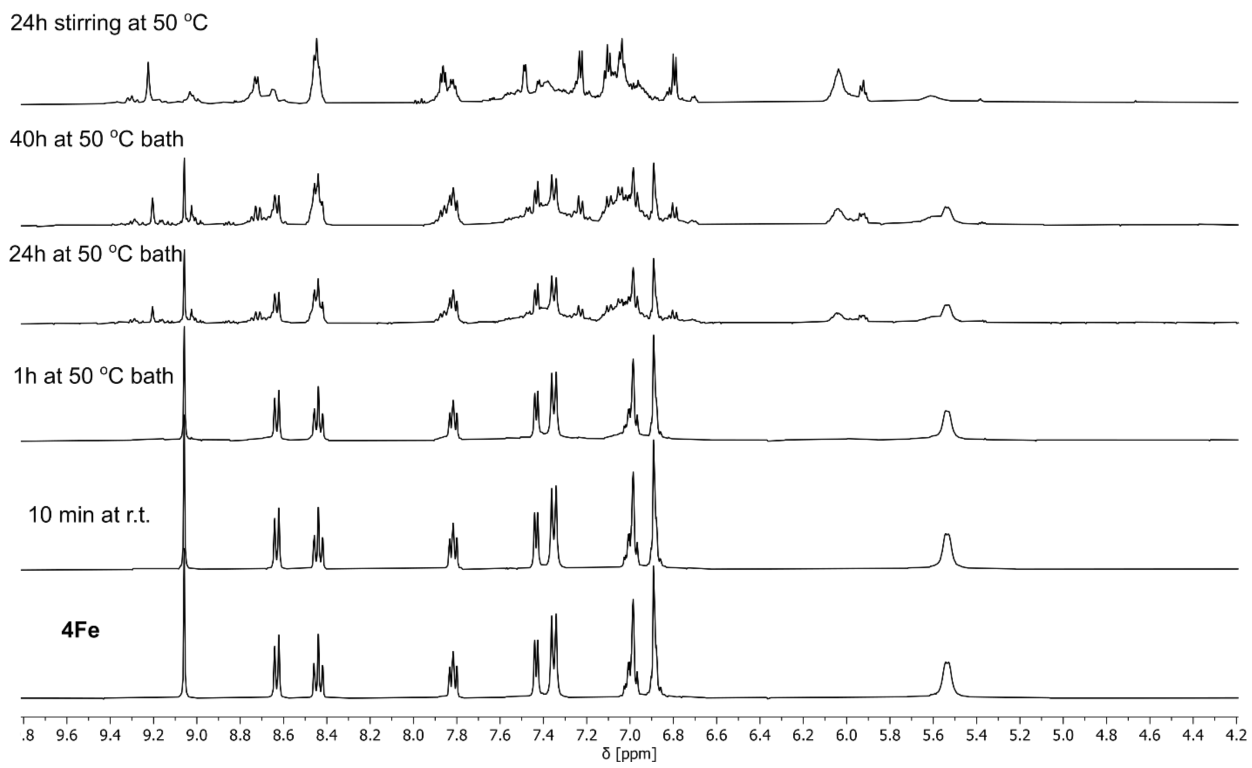

Figure S28. ^{1}H NMR-monitored progress of C_{60} binding by **4Fe**, CD_3CN, 298 K, 400 MHz.

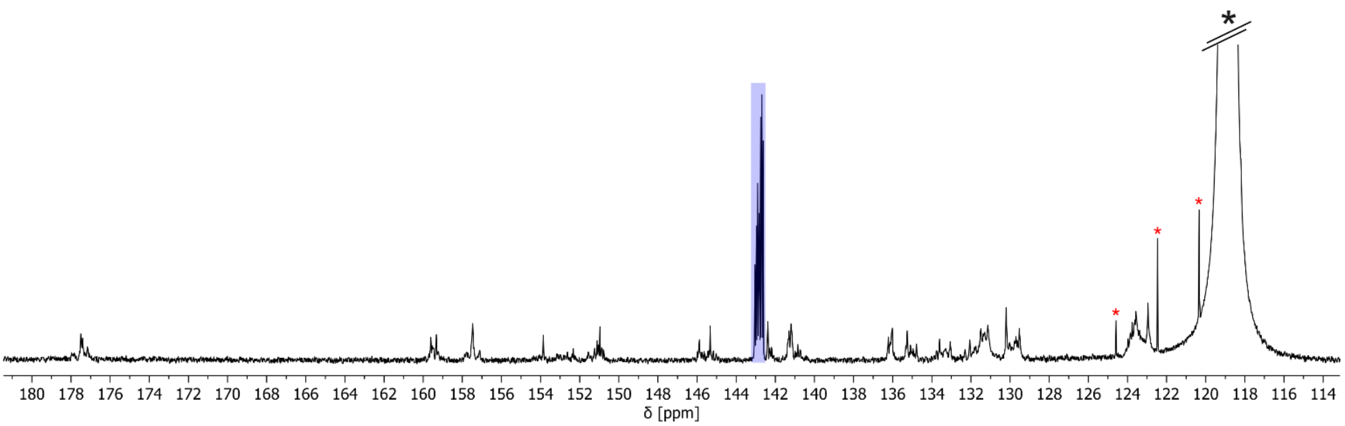

Figure S29. ^{13}C NMR spectrum of **4Fe·C$_{60}$**, CD_3CN, 298 K, 151 MHz. Zoom in of the aromatic region. Black asterisk indicates residual CD_3CN signal, and red asterisks triflimide signal (quartet due to coupling with fluorine). Blue-highlighted area contains fullerene signals from different isomers and/or orientations of fullerene within these isomers.

ESI mass spectra were recorded and showed evidence of encapsulation of C_{60} to both cages (Figures S30 and S42). For other guests, signals for host-guest adducts were not visible, suggesting that they do not survive the experimental conditions.

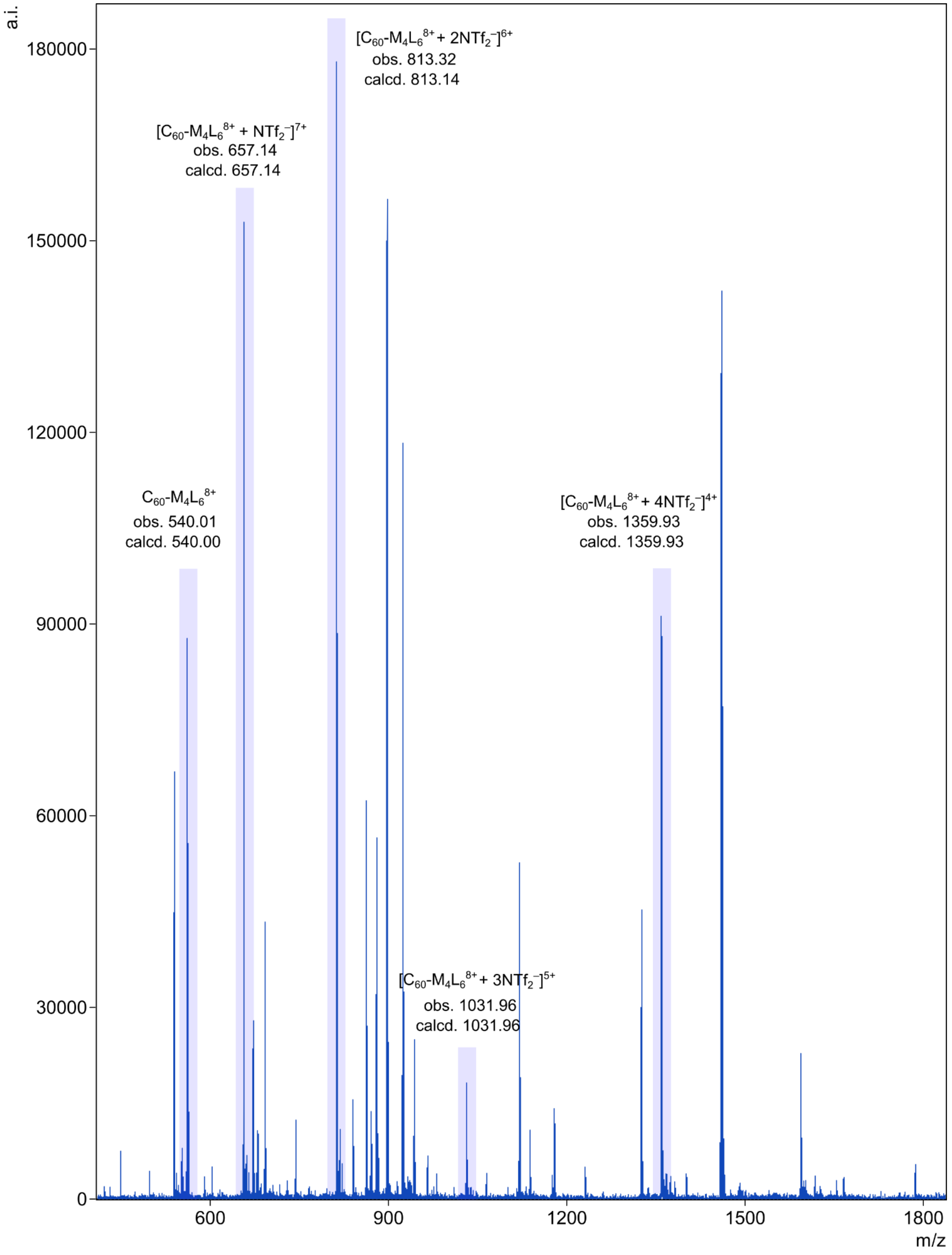

Figure S30. ESI-TOF mass spectrum of **4Fe·C$_{60}$**, view on the full spectrum.

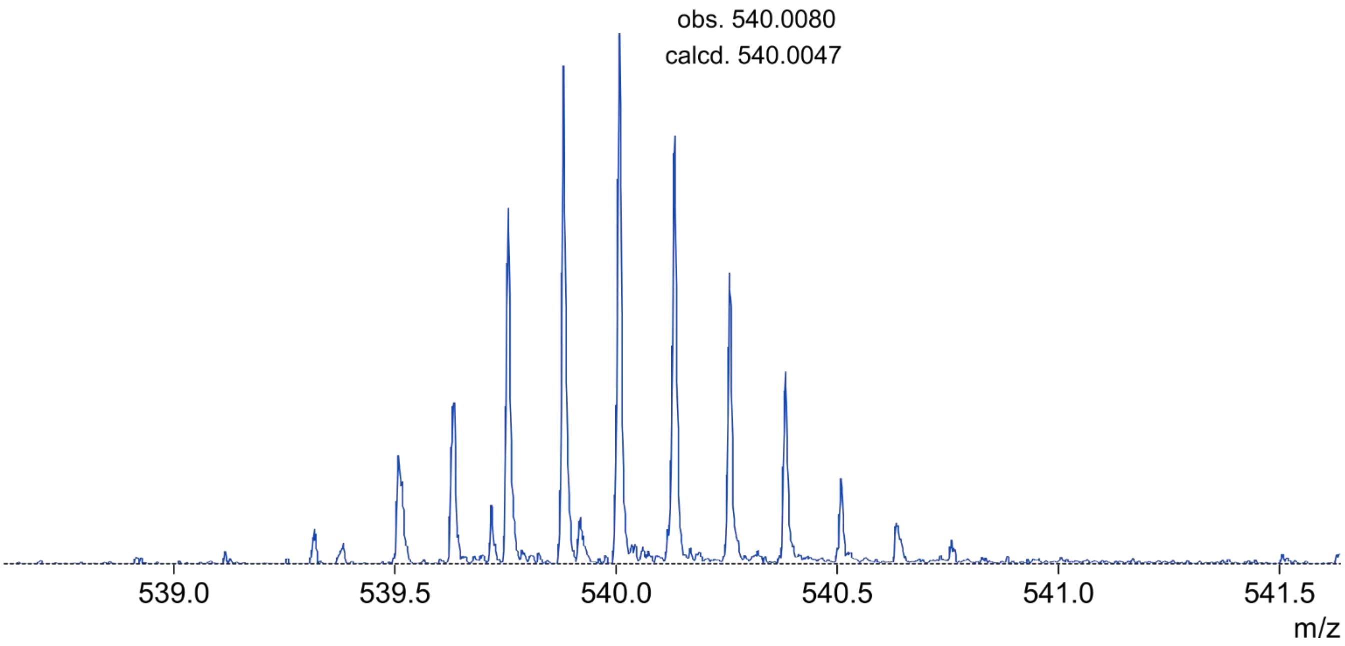

Figure S31. Zoom in of the isotopic pattern of the octacation of **4Fe·C$_{60}$**.

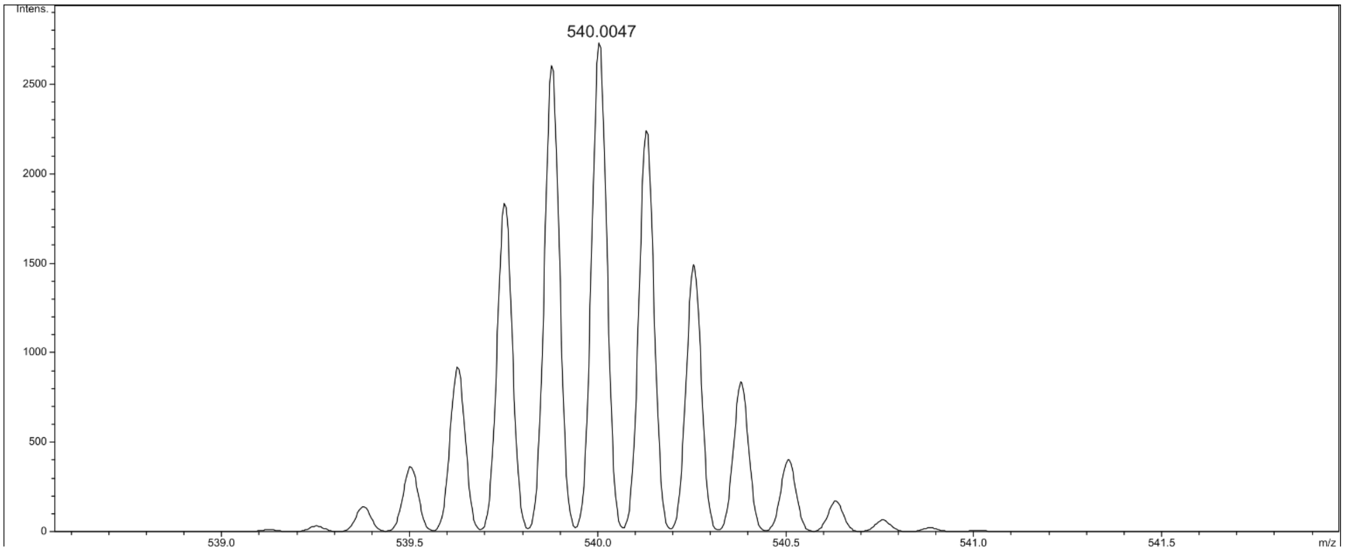

Figure S32. Simulated isotopic pattern of the octacation of **4Fe·C$_{60}$**.

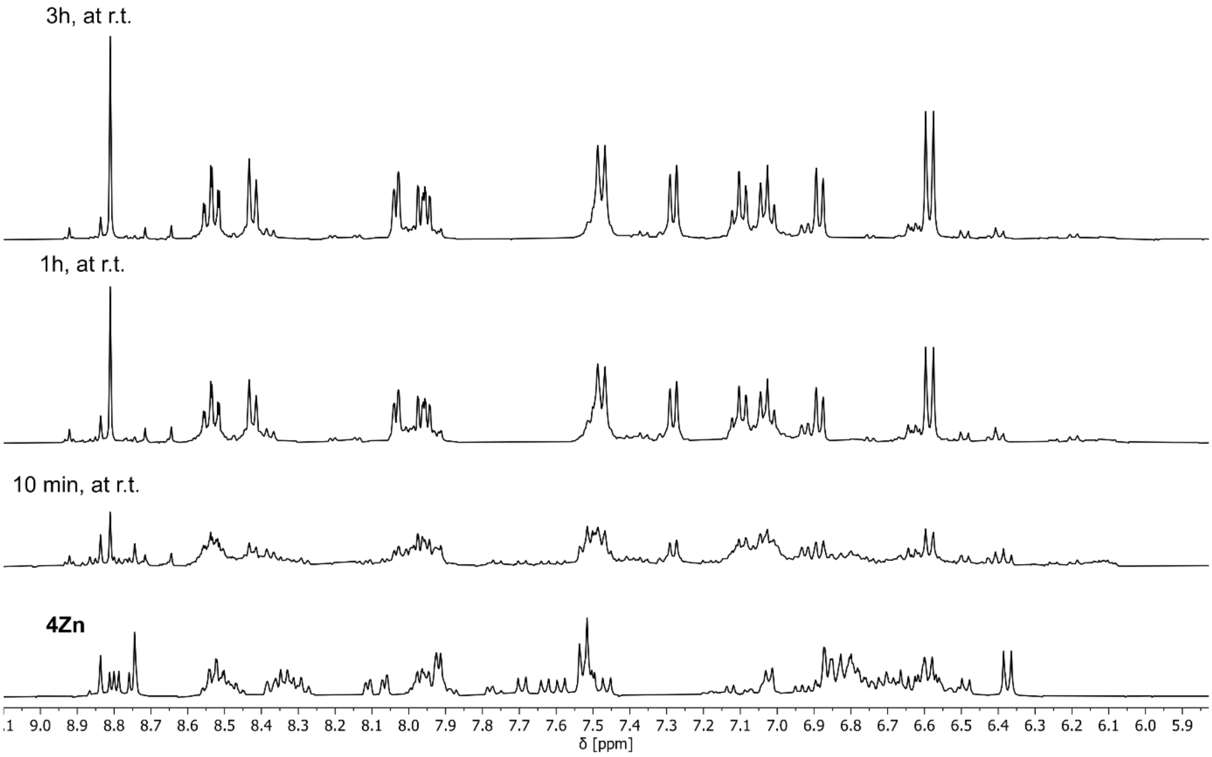

Figure S33. ^{1}H NMR-monitored progress of C_{60} binding by **4Zn**, CD_3CN, 298 K, 400 MHz.

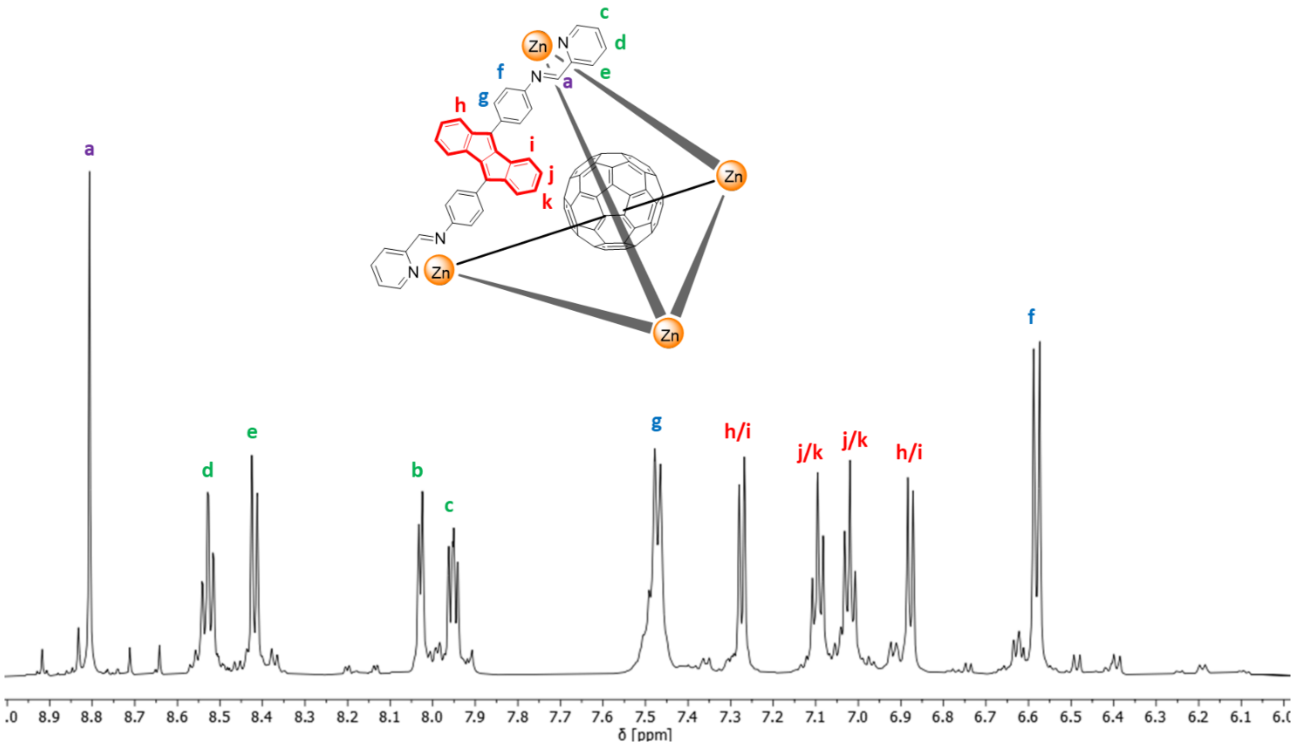

Figure S34. ^{1}H NMR spectrum of **4Zn·C60**, CD_3CN, 298 K, 600 MHz. Zoom in of the aromatic region with partial signals assignment.

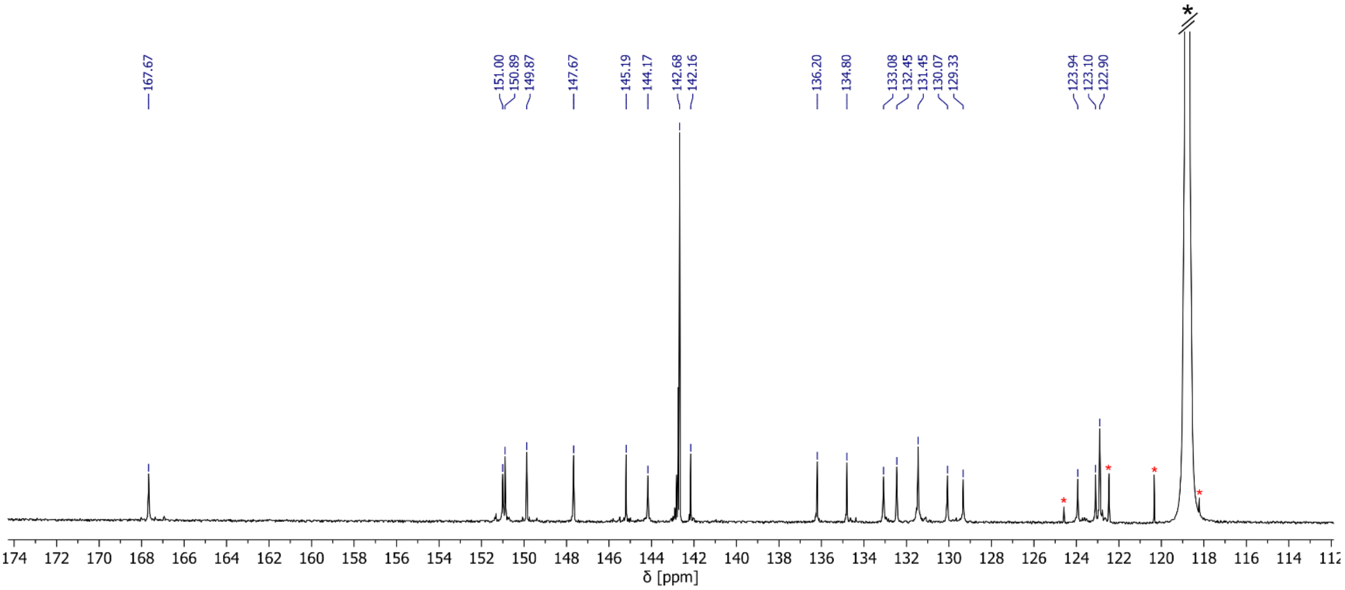

Figure S35. ^{13}C NMR spectrum of **4Zn·C_{60}**, CD_3CN, 298 K, 600 MHz. Zoom in of the aromatic region. Black asterisk indicates residual CD_3CN signal and red asterisks triflimide signal (quartet due to coupling with fluorine). Indicated peaks correspond to the main *T* isomer and bound C_{60}.

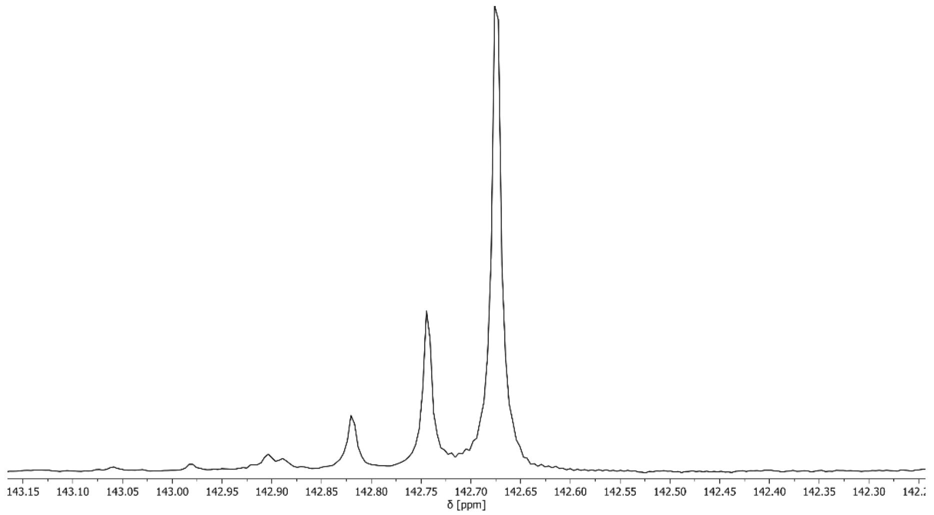

Figure S36. Zoom in on the fullerene signal in the ^{13}C NMR spectrum of **4Zn·C_{60}**. The highest peak corresponds to the fullerene encapsulated in the *T* isomer; the smaller peaks likely correspond to the fullerene encapsulated by other isomers present in solution.

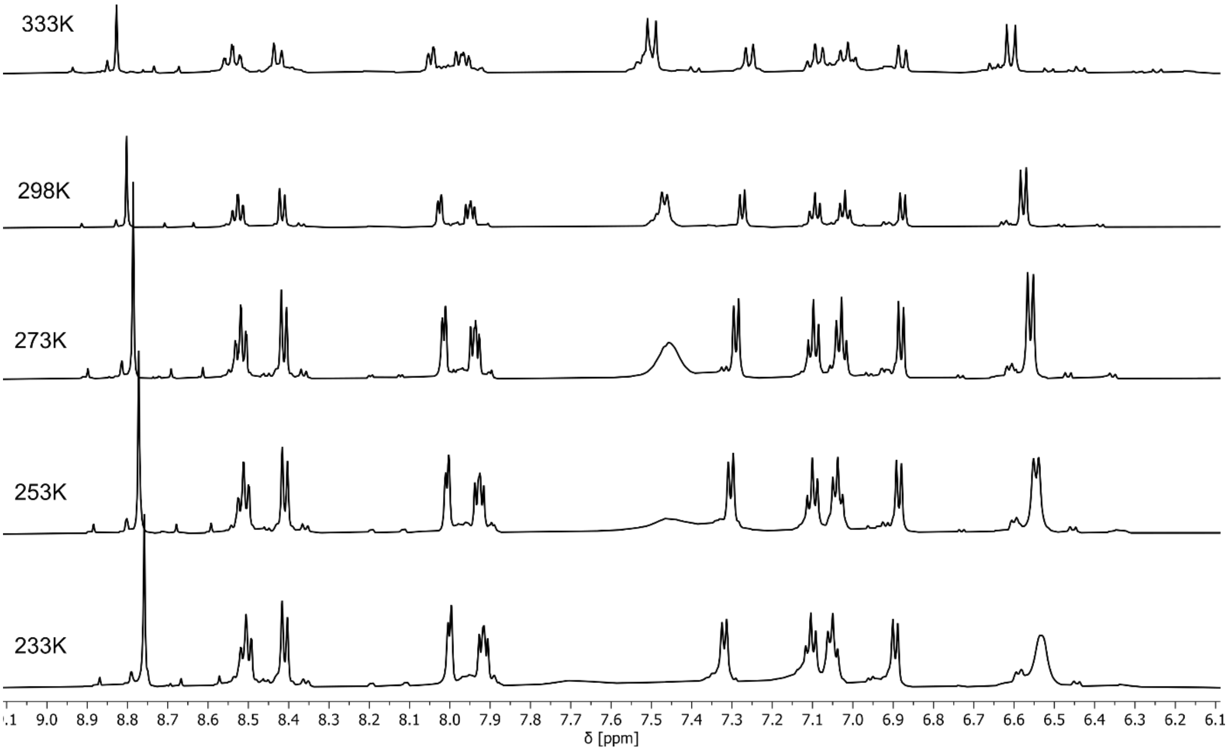

Figure S37. Variable-temperature ^{1}H NMR spectra of **4Zn·C$_{60}$**, CD_3CN, 600 MHz.

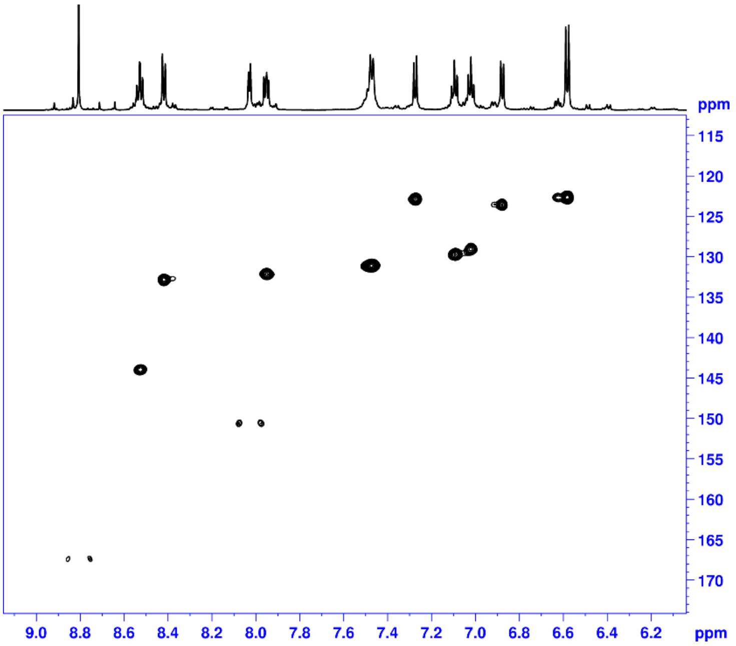

Figure S38. ^{1}H-^{13}C HSQC NMR spectrum of **4Zn·C$_{60}$**, CD_3CN, 298 K, 600 MHz.

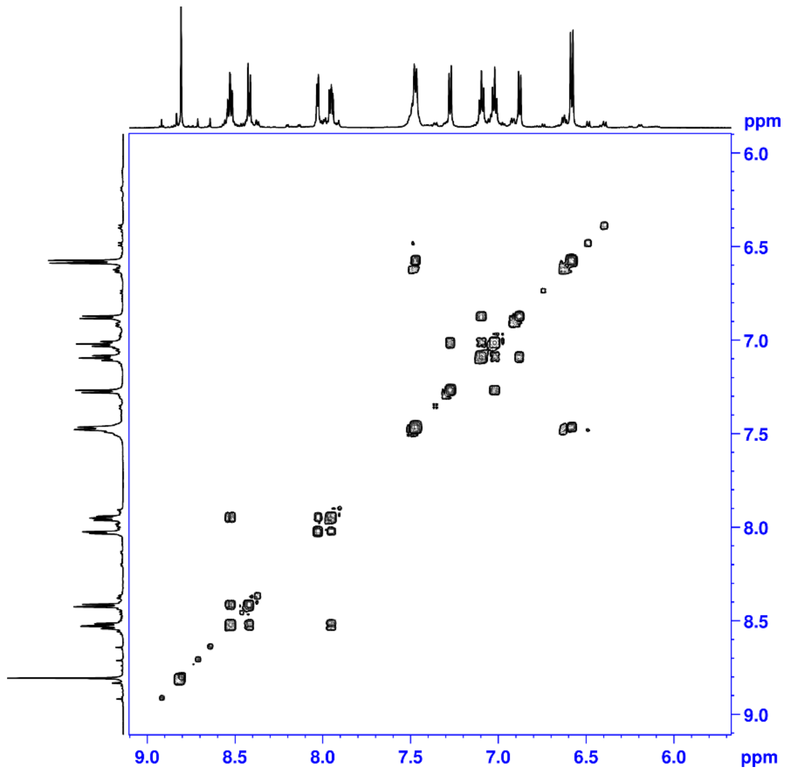

Figure S39. ^{1}H-^{1}H COSY NMR spectrum of **4Zn·C$_{60}$**, CD_3CN, 298 K, 600 MHz.

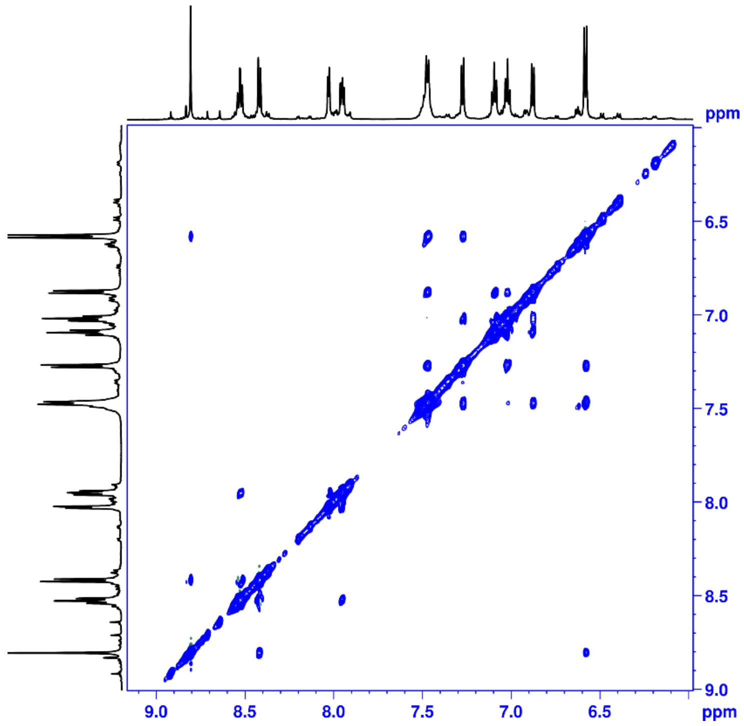

Figure S40. ^{1}H-^{1}H NOESY NMR spectrum of **4Zn·C$_{60}$**, CD_3CN, 298 K, 600 MHz.

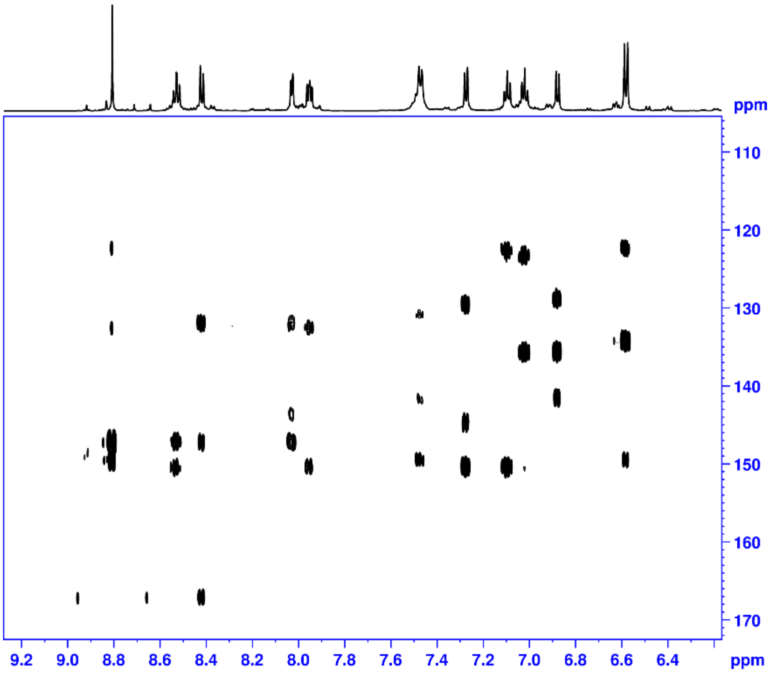

Figure S41. ^{1}H-^{13}C HMBC NMR spectrum of **4Zn·C$_{60}$**, CD_3CN, 298 K, 600 MHz.

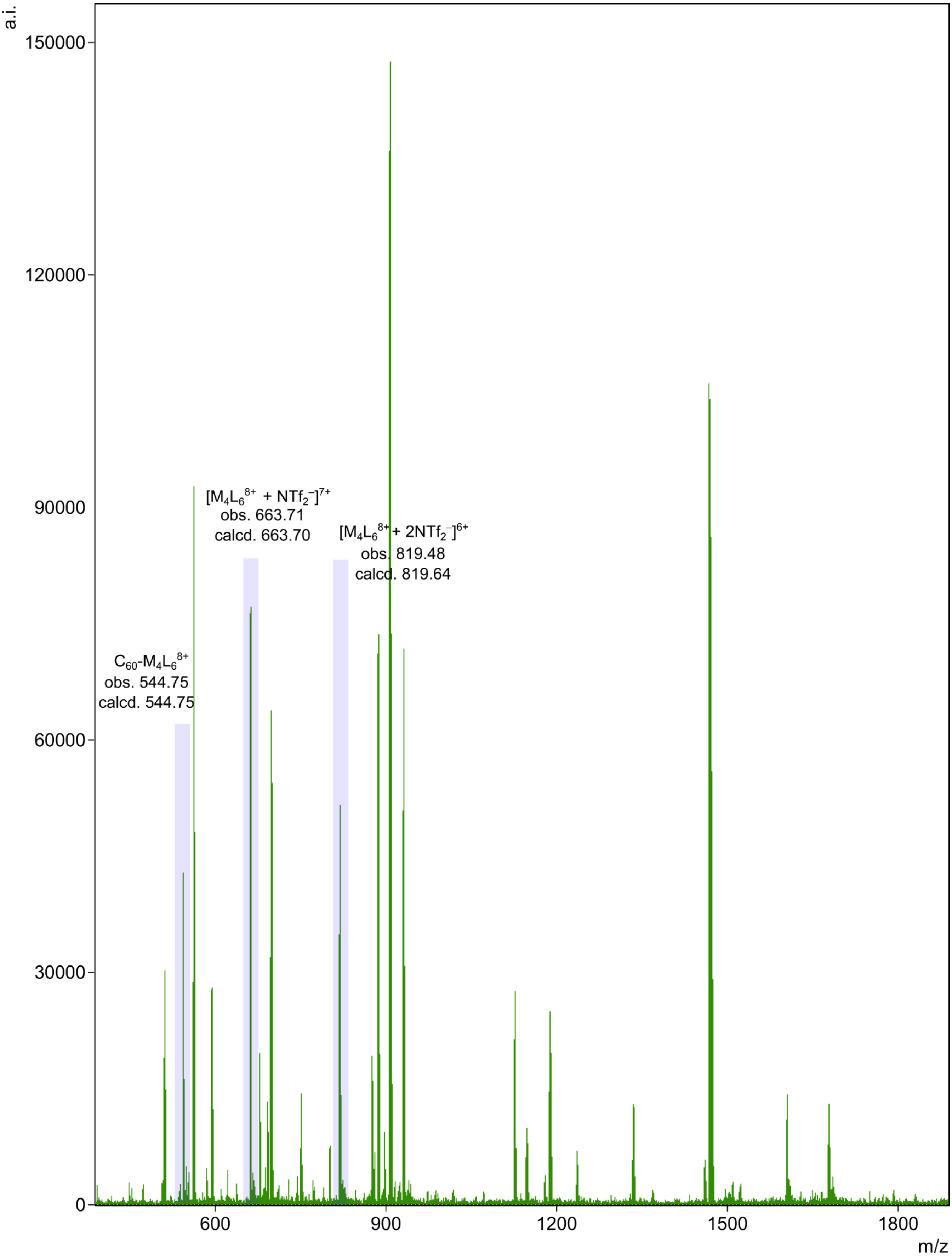

Figure S42. ESI-TOF mass spectrum of **4Zn·C60**, view on the full spectrum.

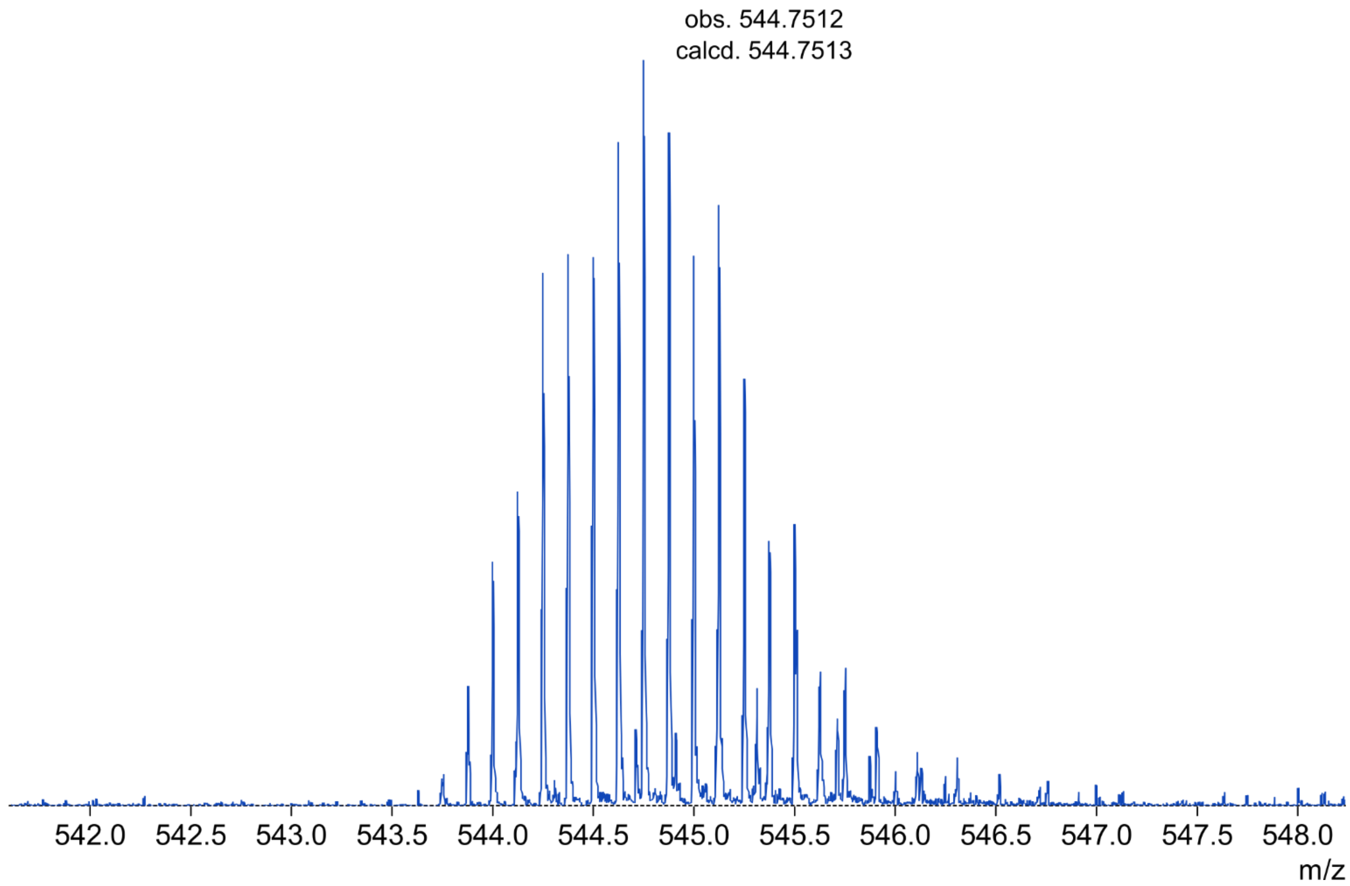

Figure S43. Zoom in of the isotopic pattern of octacation of **4Zn·C$_{60}$**.

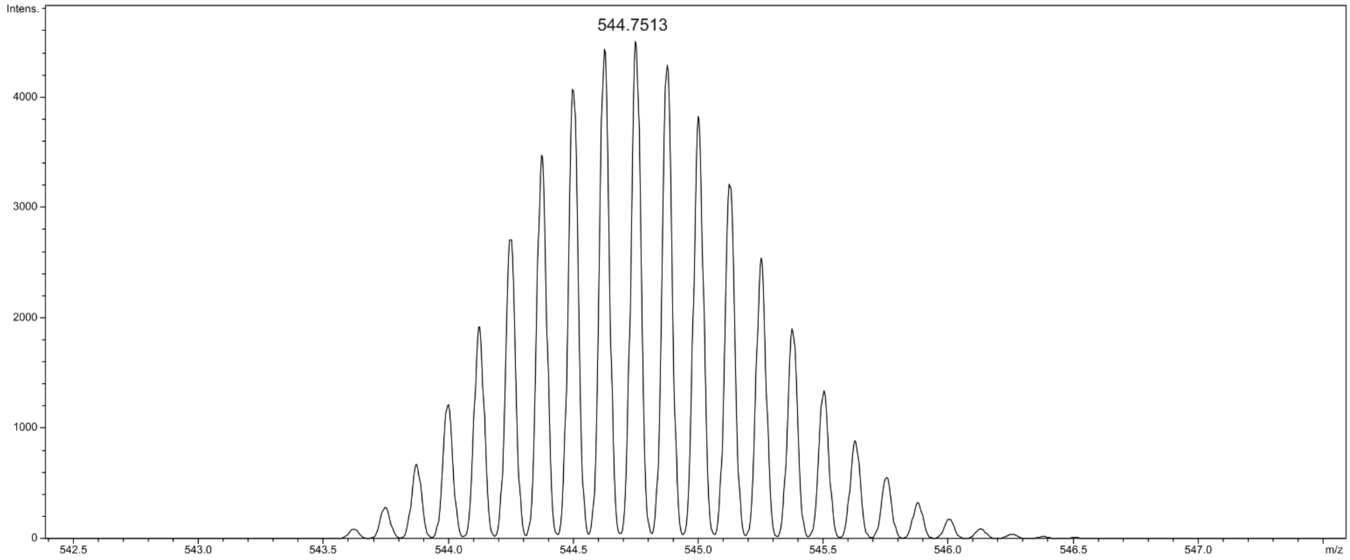

Figure S44. Simulated isotopic pattern of the octacation of **4Zn·C$_{60}$**.

4.1.2. Binding C_{70}

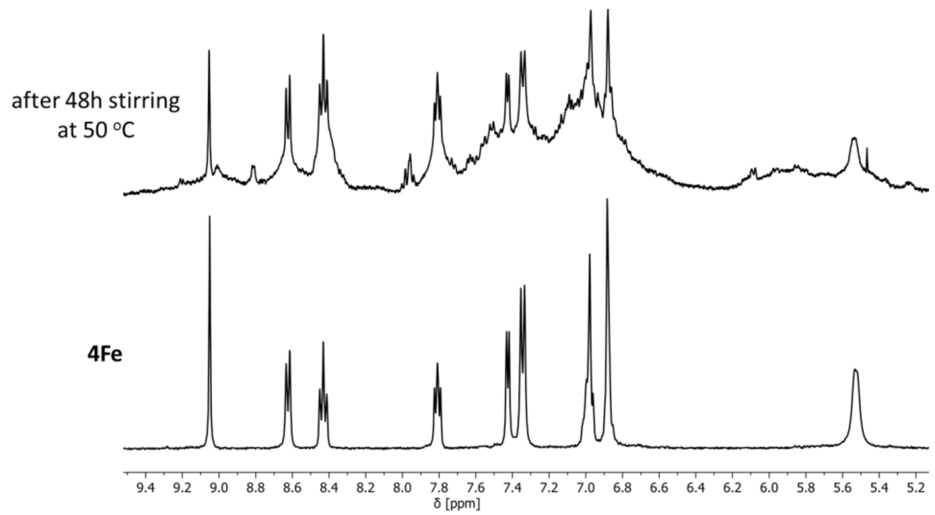

Figure S45. ^{1}H NMR spectrum of **4Fe** before (bottom) and after (top) stirring with an excess of C_{70} at 50 °C for one week, CD_3CN, 298 K, 400 MHz.

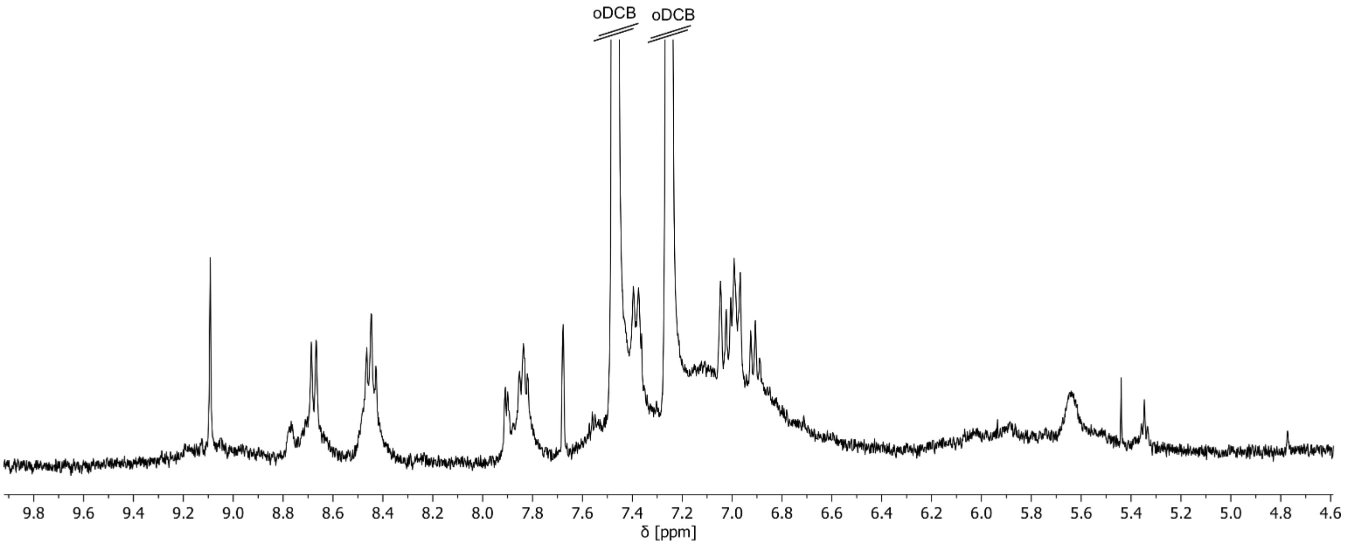

Figure S46. ^{1}H NMR spectrum of **4Fe** after stirring with an excess of C_{70} at 50 °C for one week and then, after addition of *o*-dichlorobenzene, stirring at 50 °C for 16 h, CD_3CN, 298 K, 400 MHz.

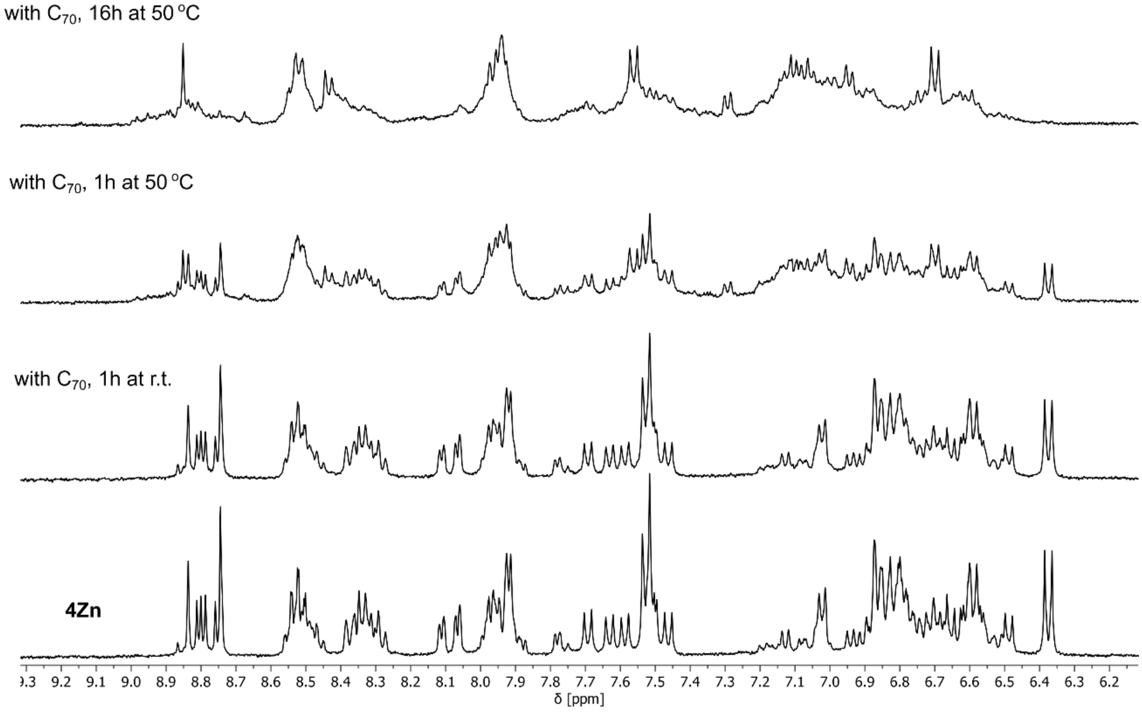

Figure S47. ^{1}H NMR-monitored progress of C_{70} binding by **4Zn**, CD_3CN, 298 K, 400 MHz.

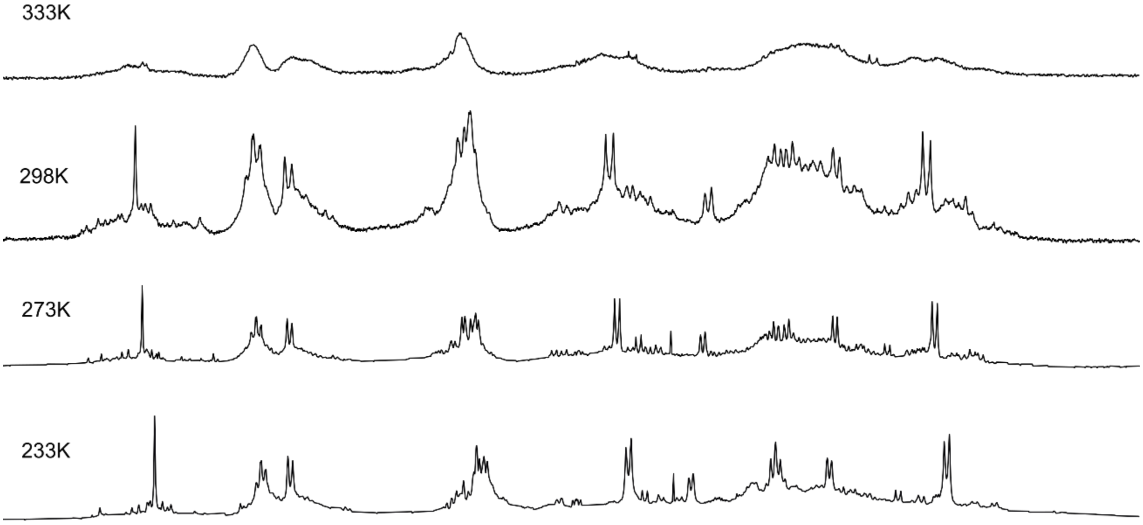

Figure S48. Variable-temperature ^{1}H NMR spectra of **4Zn·C₇₀**, CD_3CN, 600 MHz.

ESI mass spectra did not show peaks corresponding to the C_{70} complex. The cage likely is not stabilized by C_{70} due to a poorer match to the cavity (oval shape of fullerene C_{70}).

4.1.3. Fullerene displacement

The solution of **4Zn** was treated with an excess of C_{70} and stirred overnight at 45 °C. The NMR spectrum showed no difference (Figure S49). However, when **4Zn·C$_{70}$** was treated with C_{60}, full conversion to **4Zn·C$_{60}$** was observed (Figure S50).

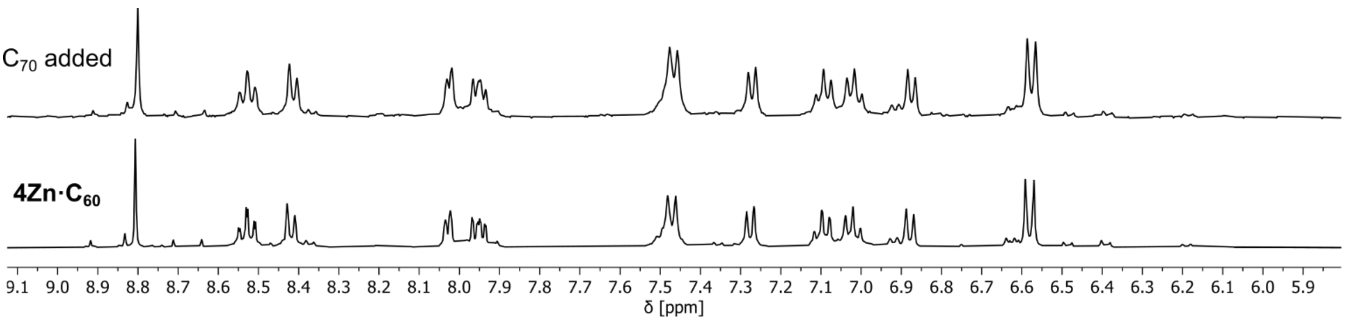

Figure S49. Comparison of the ^{1}H NMR spectra of **4Zn·C$_{60}$** before (bottom) and after (top) adding C_{70}, CD_3CN, 298 K, 400 MHz.

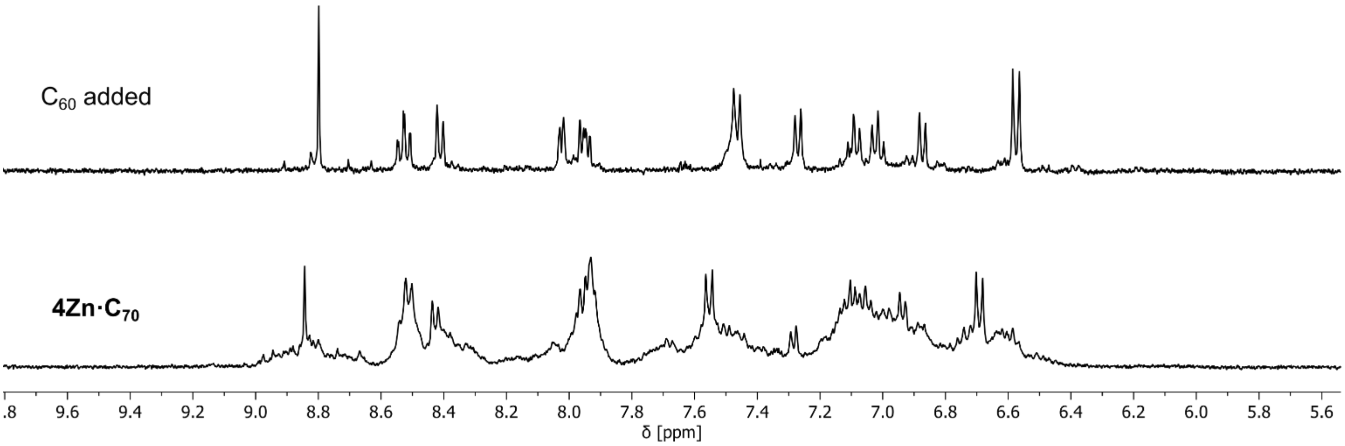

Figure S50. Comparison of the ^{1}H NMR spectra of **4Zn·C$_{70}$** before (bottom) and after (top) adding C_{60}, CD_3CN, 298 K, 400 MHz.

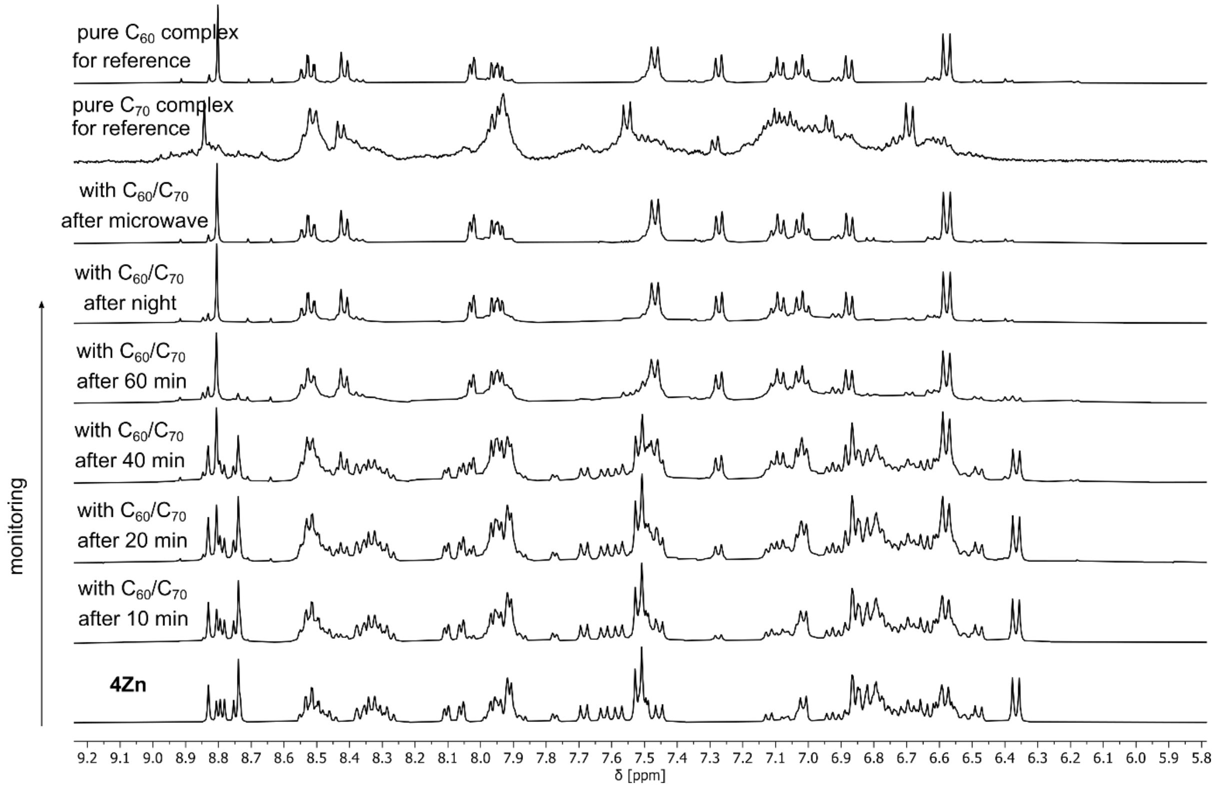

Figure S51. NMR spectra obtained during monitoring 1:1 mixture of C_{60} and C_{70} fullerenes with **4Zn** over time, and comparison with a spectrum after exposing this mixture to 140 °C in a microwave reactor for 1h. CD_3CN, 400 MHz, 300 K.

4.2. Small-molecule binding

A solution of the cage (ca. 1 mM) in CD_3CN was stirred with 4–8 equiv. of guest present overnight at room temperature, after which NMR spectra were recorded. In general, most of the used guest molecules have a low solubility in acetonitrile and as such, knowing the exact added amount is never very exact. For this reason, we also have not determined binding constant and only looked for the signs of binding interactions between the host and guest molecules, as often done in the literature with similar cages.[4,5] In some cases, we were able to monitor the progress of the binding by adding an increasing amount of the guest.

We were able to confirm whether the guest is in fast/slow exchange regime on the NMR timescale by measuring NOESY (Nuclear Overhauser Effect Spectroscopy) or ROESY (Rotating-frame Overhauser Effect Spectroscopy) spectra. These two experiments work in a slightly different way, depending on the molecular size. With the size regime represented by the M_4L_6 cages, it works as follows: the NOEs for through-space correlations (NOE) and for the exchange correlations (EXSY) have the same sign and therefore they cannot be distinguished from each other. It is therefore not possible to say whether a cross peak corresponds to an NOE signal between some protons within the cage, an NOE signal between the cage and a guest, or a signal between the bound and free guest, if they are visible separately due to slow exchange. In this situation, ROESY is helpful, because with this size regime, in the ROESY spectra, the NOE-like and EXSY peaks can be distinguished: the NOE-like peaks are in phase with the diagonal (same sign), whereas the EXSY peaks appear with a sign opposite to the diagonal.

If the guest is in slow exchange on the time scale of the NMR experiment, there are two sets of signals of the guest (compared to only one set of signals, shifted, in case of fast exchange). When excess of guest is added, two different sets of guest signals appear if the guest is in slow exchange, and they will give an EXSY signal to each other. These signals can be confirmed as EXSY peaks by measuring ROESY and checking whether the sign of these peaks is in-phase or out-of-phase with the diagonal. This enables identifying a slow exchange of the guest. If the signals were corresponding to the cage walls, or interactions between the cage walls and the encapsulated guest, they would appear in-phase with the diagonal.

4.2.1. Cage 4Fe with pyrene

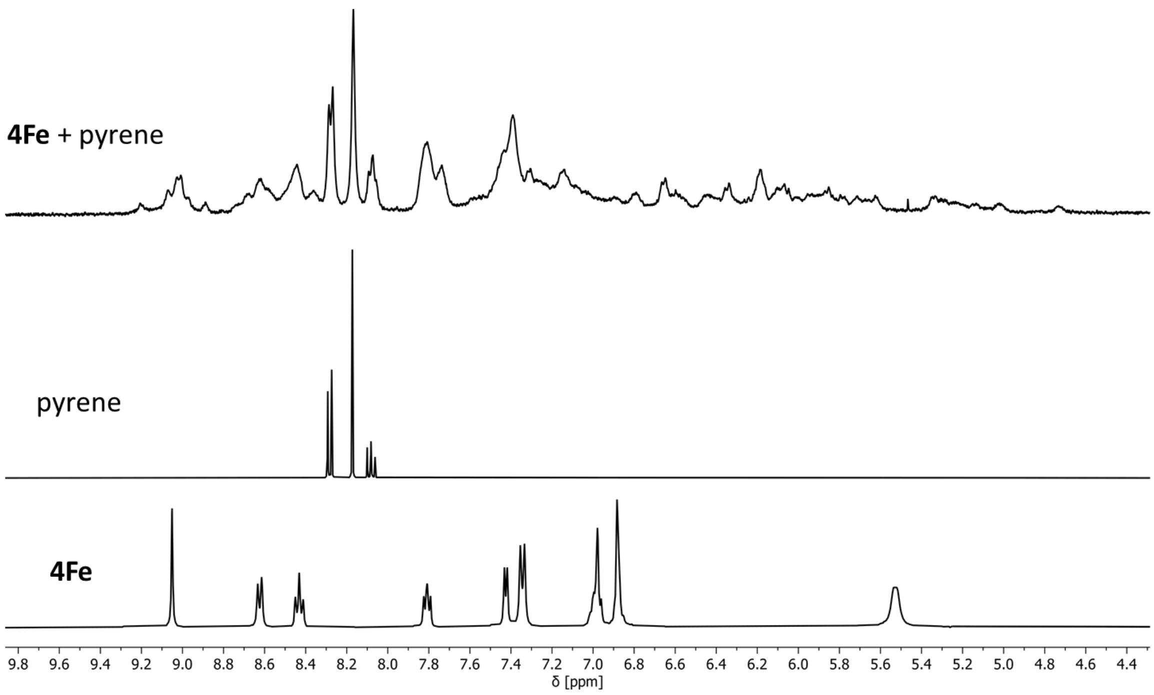

Figure S52. Comparison of ^{1}H NMR spectra of **4Fe**, pyrene and a mixture of **4Fe** with pyrene (ca. 6 equiv). CD_3CN, 298 K, 400 MHz.

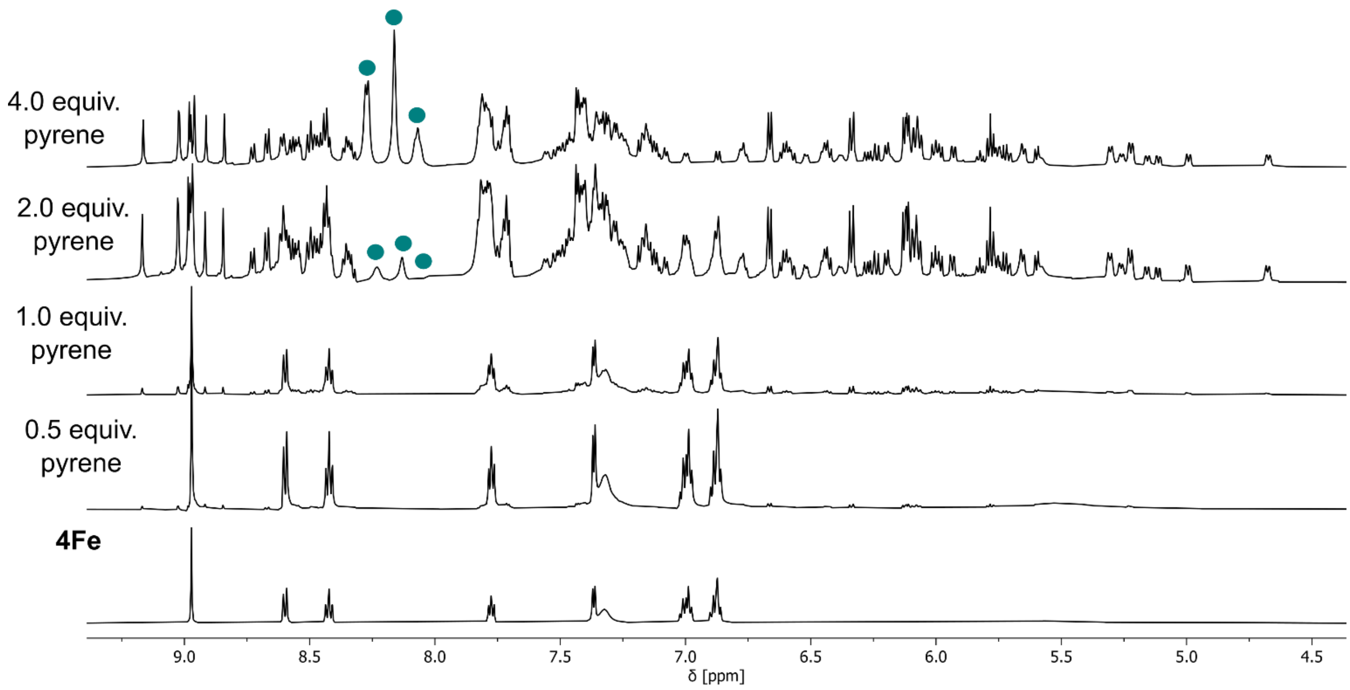

Figure S53. Comparison of ^{1}H NMR spectra of **4Fe** containing different amounts of pyrene. CD_3CN, 273 K, 600 MHz. Blue dots indicate signals of the excess guest.

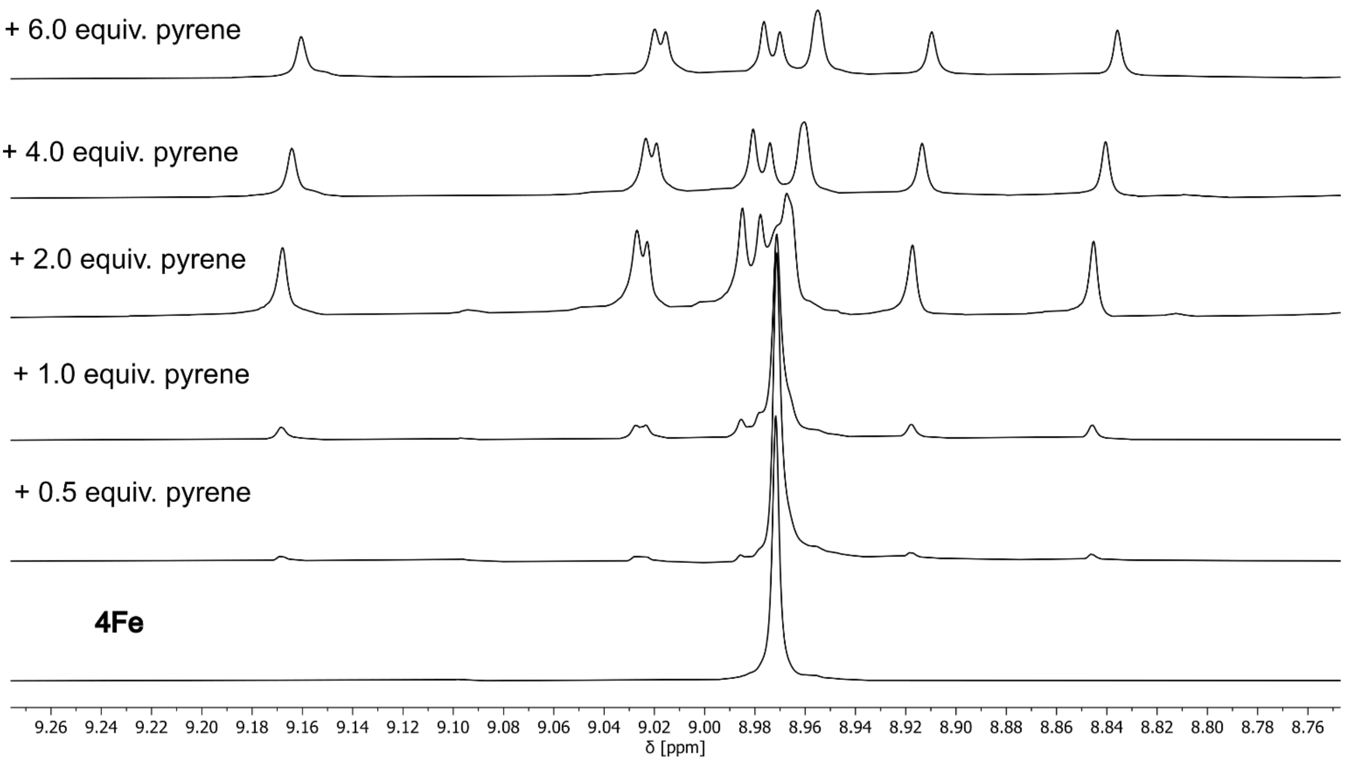

Figure S54. Zoom in of the imine peaks of the ^{1}H NMR spectra of **4Fe** containing different amounts of pyrene. CD_3CN, 273 K, 600 MHz.

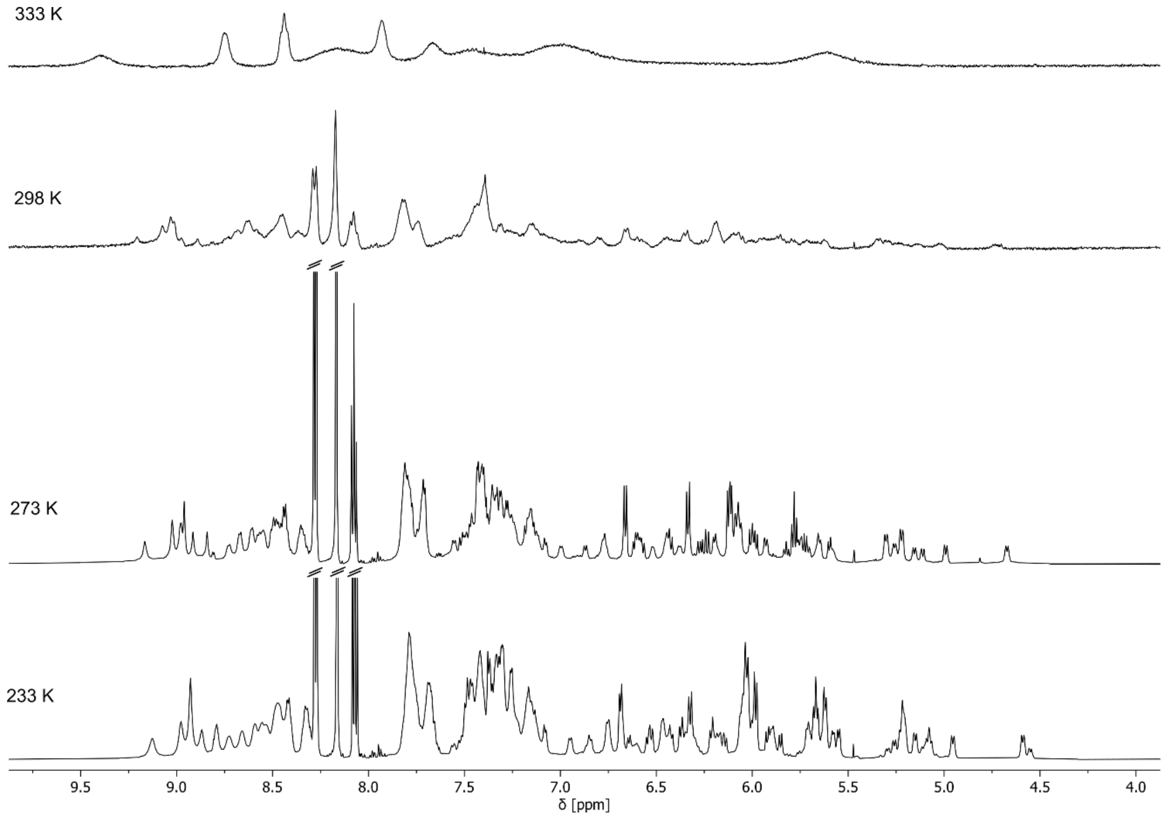

Figure S55 Variable-temperature ^{1}H NMR spectra of **4Fe** containing pyrene (6 equiv.). Note that for the high-temperature measurement, a different sample was used. CD_3CN, 600 MHz.

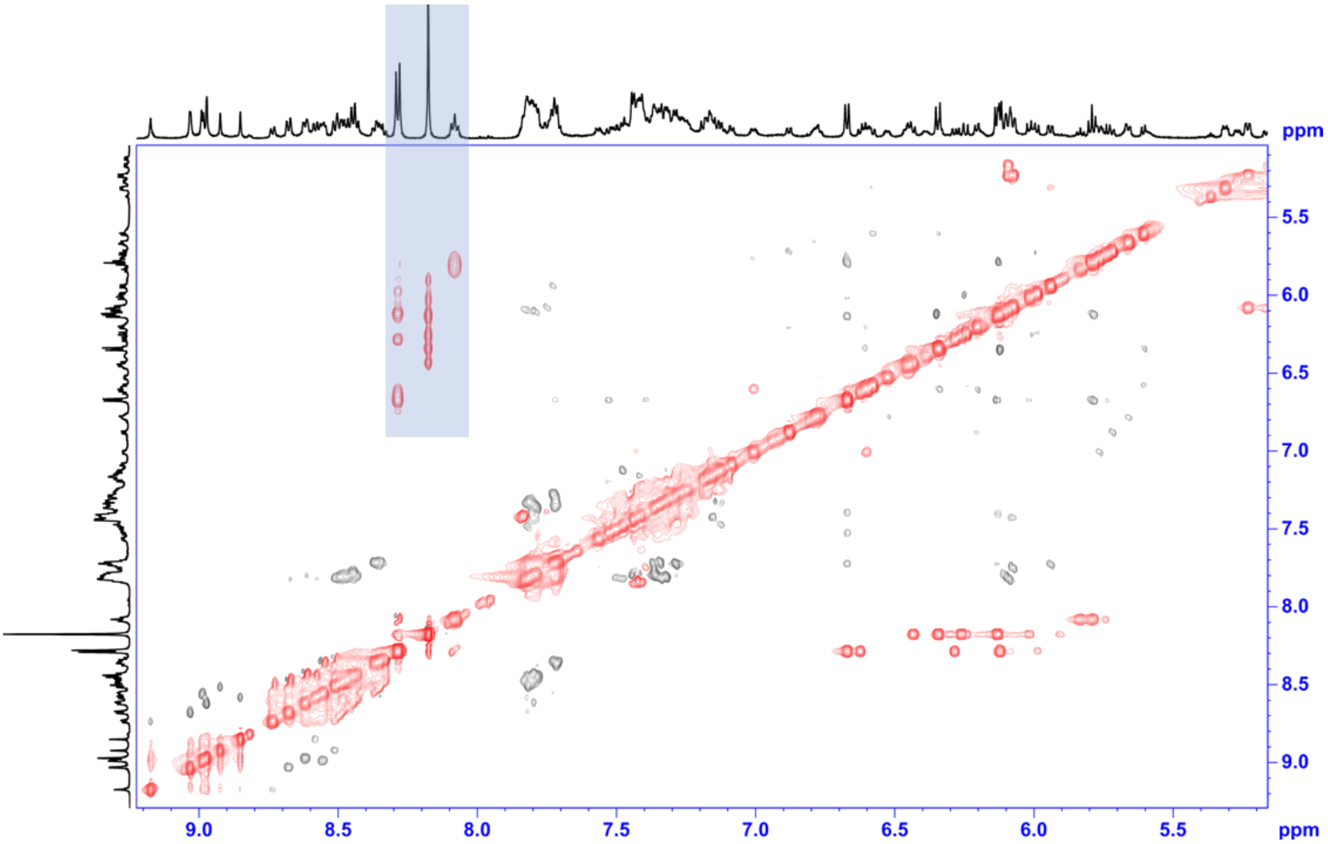

Figure S56. ^{1}H-^{1}H ROESY NMR spectrum of **4Fe** with pyrene (6 equiv.), CD_3CN, 273 K, 600 MHz. Exchange correlations of the free guest are highlighted in blue.

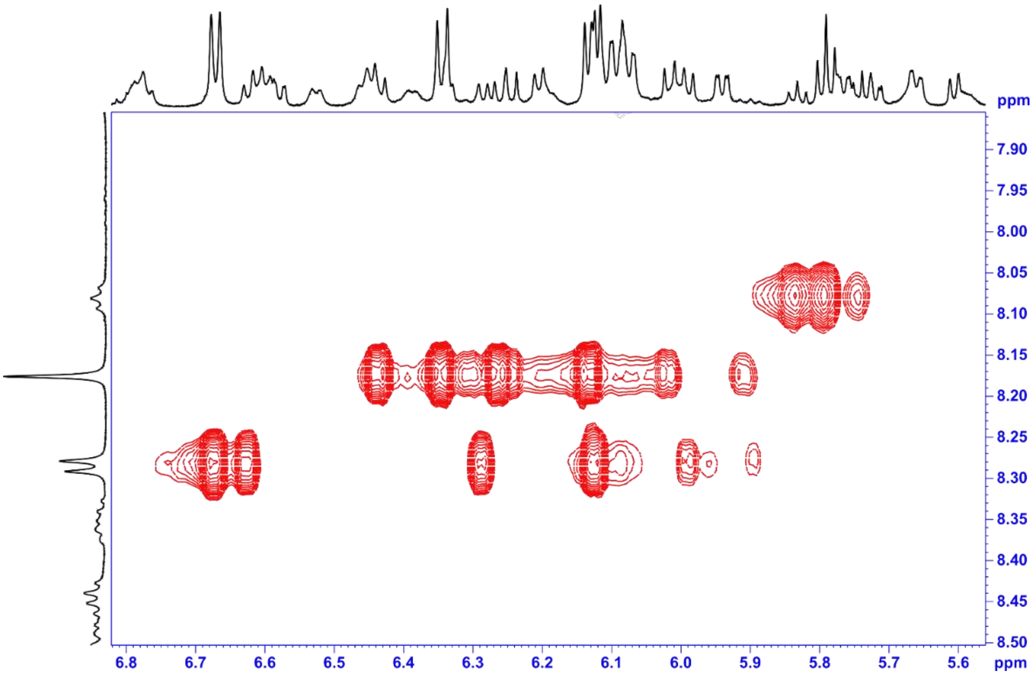

Figure S57. Zoom in of the ^{1}H-^{1}H ROESY NMR spectrum of **4Fe** with pyrene (6 equiv.), CD_3CN, 273 K, 600 MHz.

4.2.2. Cage 4Zn with pyrene

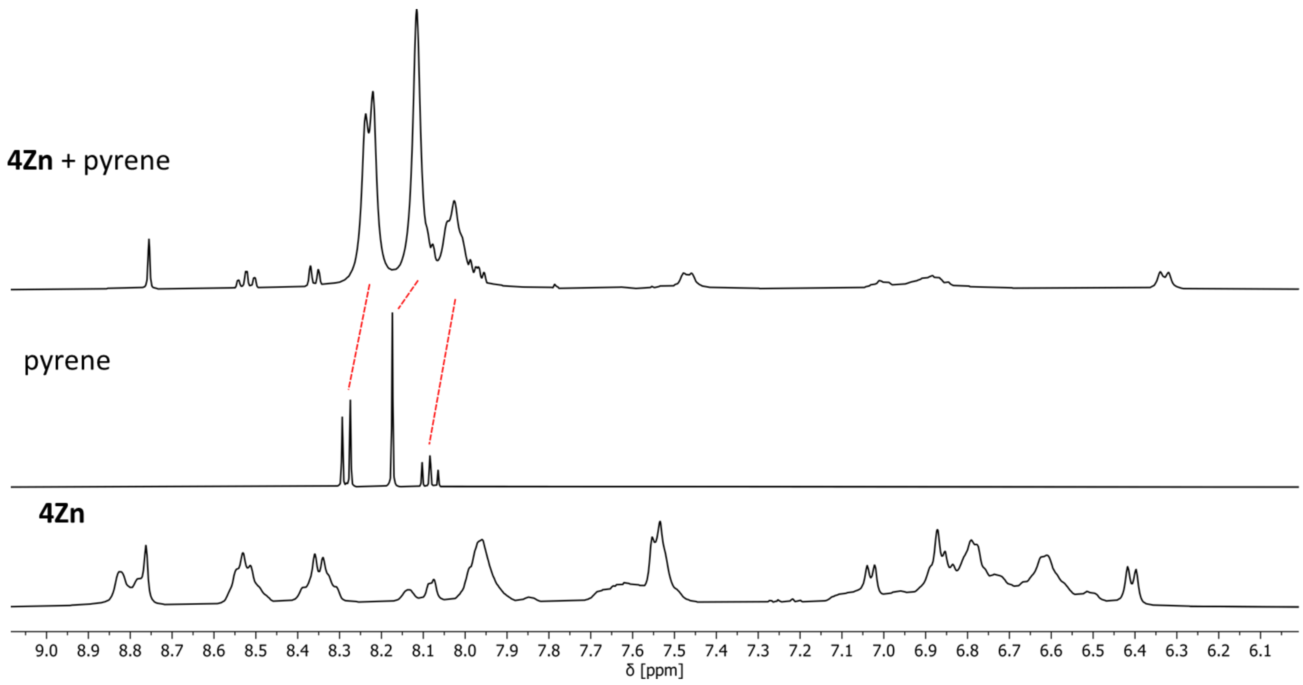

Figure S58. Comparison of ^{1}H NMR spectra of **4Zn**, pyrene and a mixture of **4Zn** with pyrene. CD_3CN, 333 K, 400 MHz. Shifts in the guest signals are marked with red dotted lines.

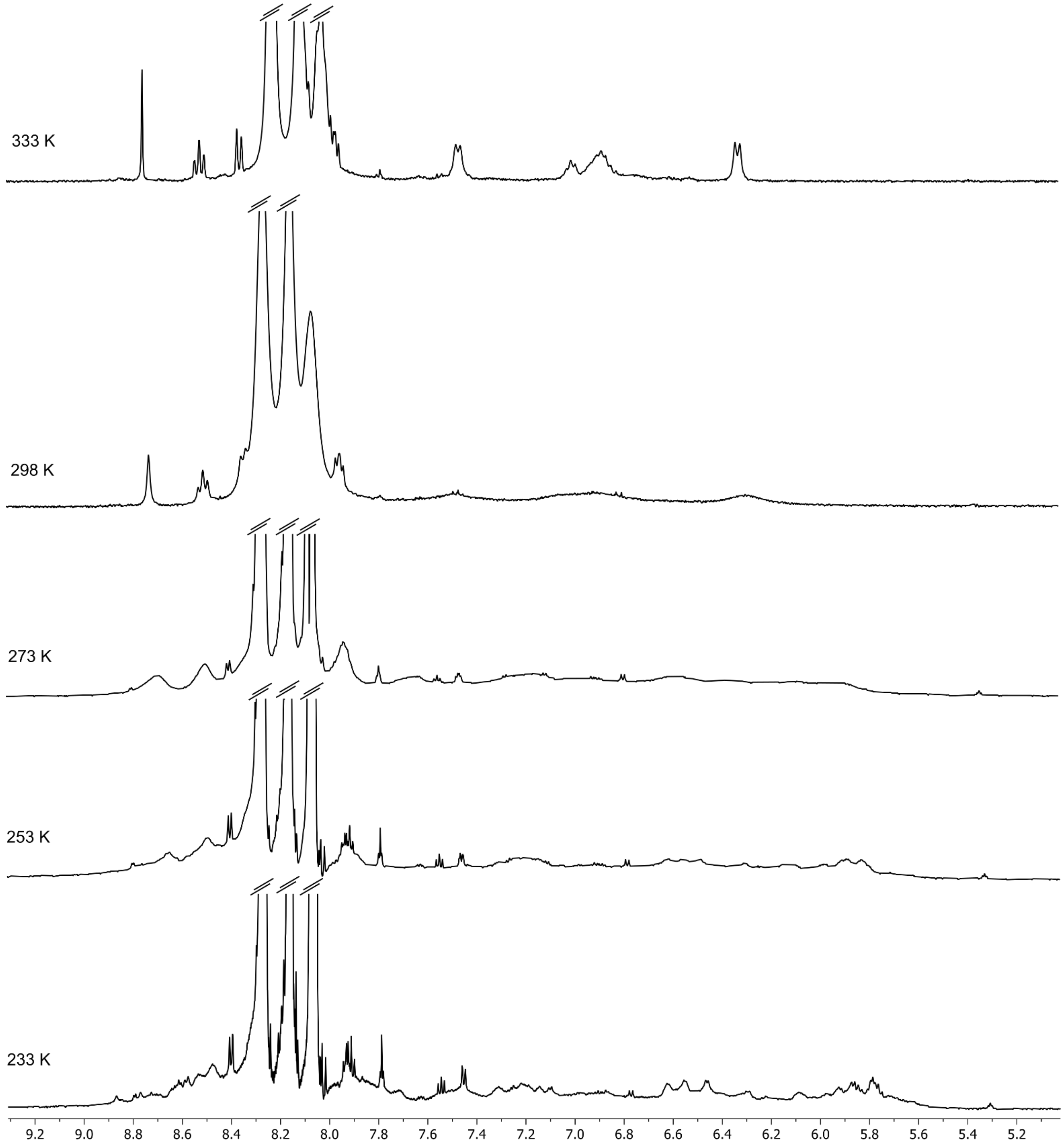

Figure S59 Variable-temperature ^{1}H NMR spectra of **4Zn** containing pyrene. CD_3CN, 600 MHz.

4.2.3. Cage 4Fe with perylene

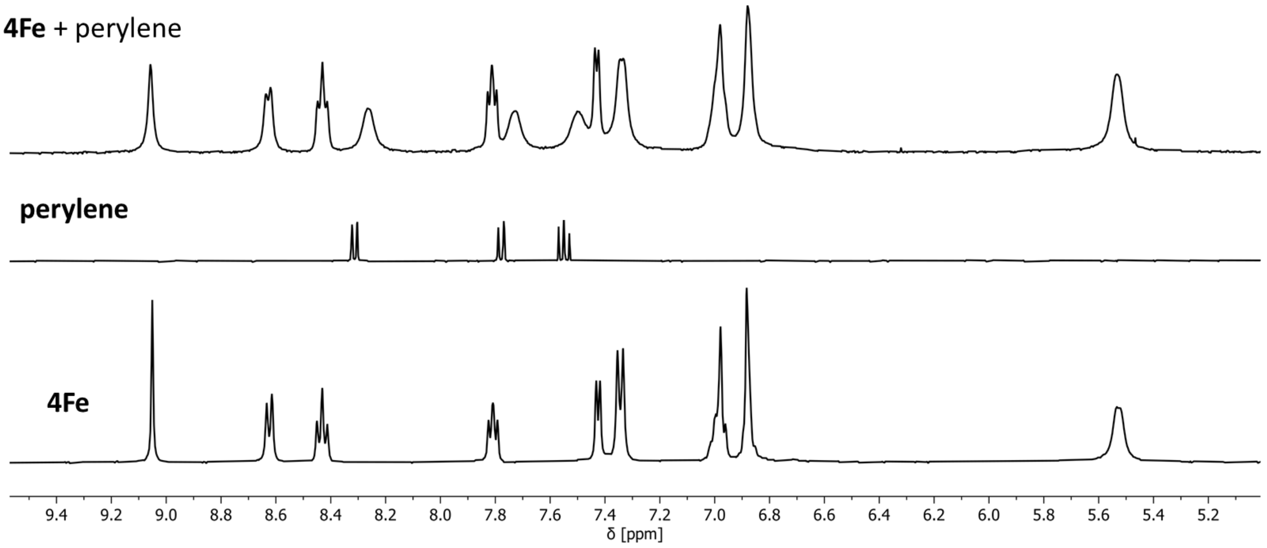

Figure S60. Comparison of ^{1}H NMR spectra of **4Fe**, perylene and a mixture of **4Fe** with perylene (ca. 8 equiv.). CD_3CN, 298 K, 400 MHz.

4.2.4. Cage 4Zn with perylene

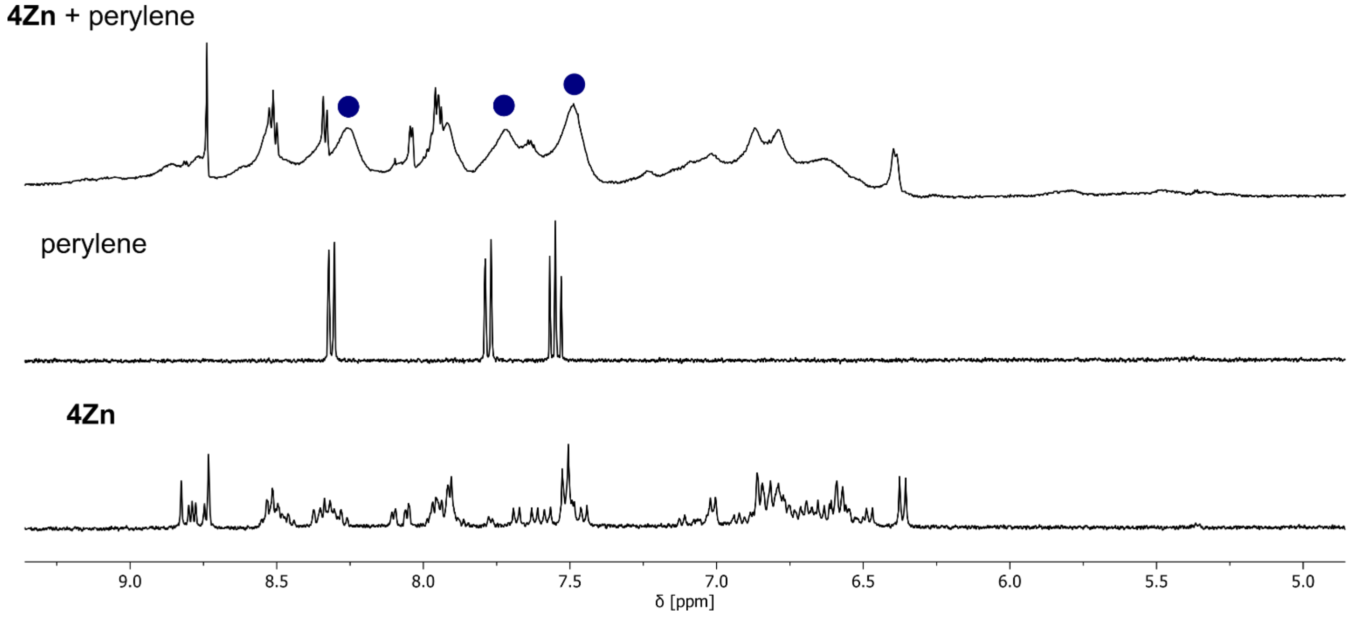

Figure S61. Comparison of ^{1}H NMR spectra of **4Zn**, perylene and a mixture of **4Zn** with perylene (ca. 6 equiv.). CD_3CN, 298 K, 400 MHz. Blue dots indicate signals of the guest.

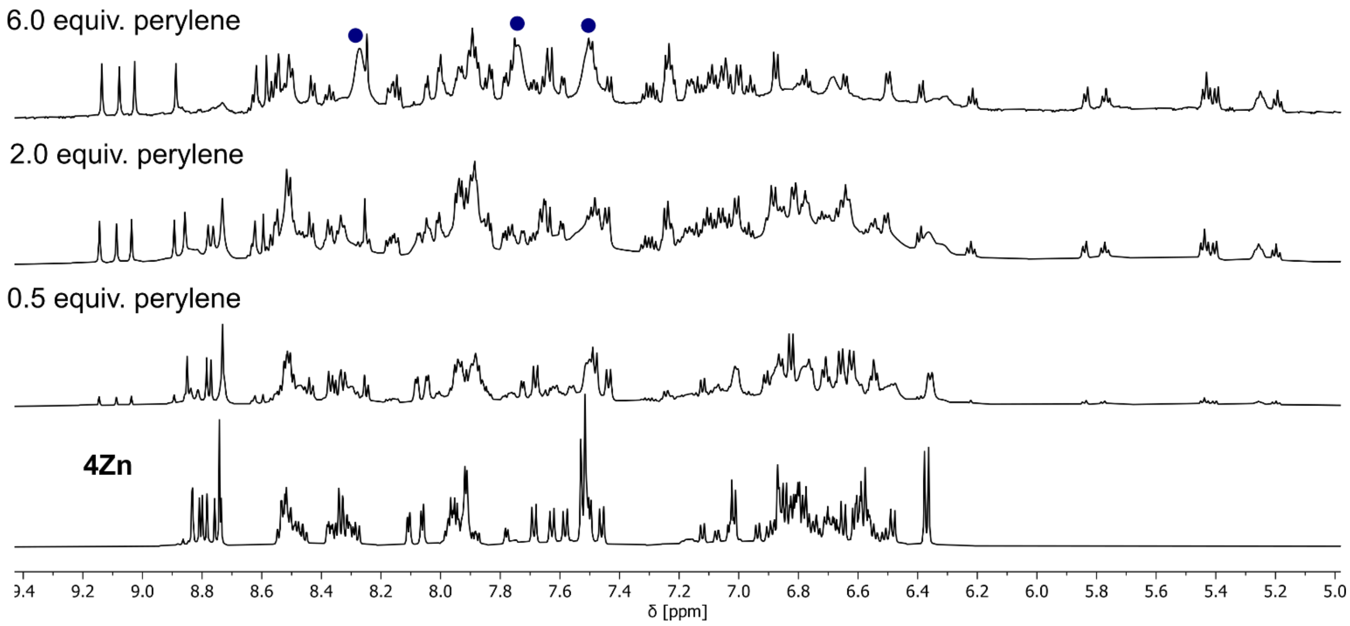

Figure S62. Comparison of ^{1}H NMR spectra of **4Zn** containing different amounts of perylene (ca. 6 equiv.). CD_3CN, 273 K, 600 MHz. Blue dots indicate signals of the guest.

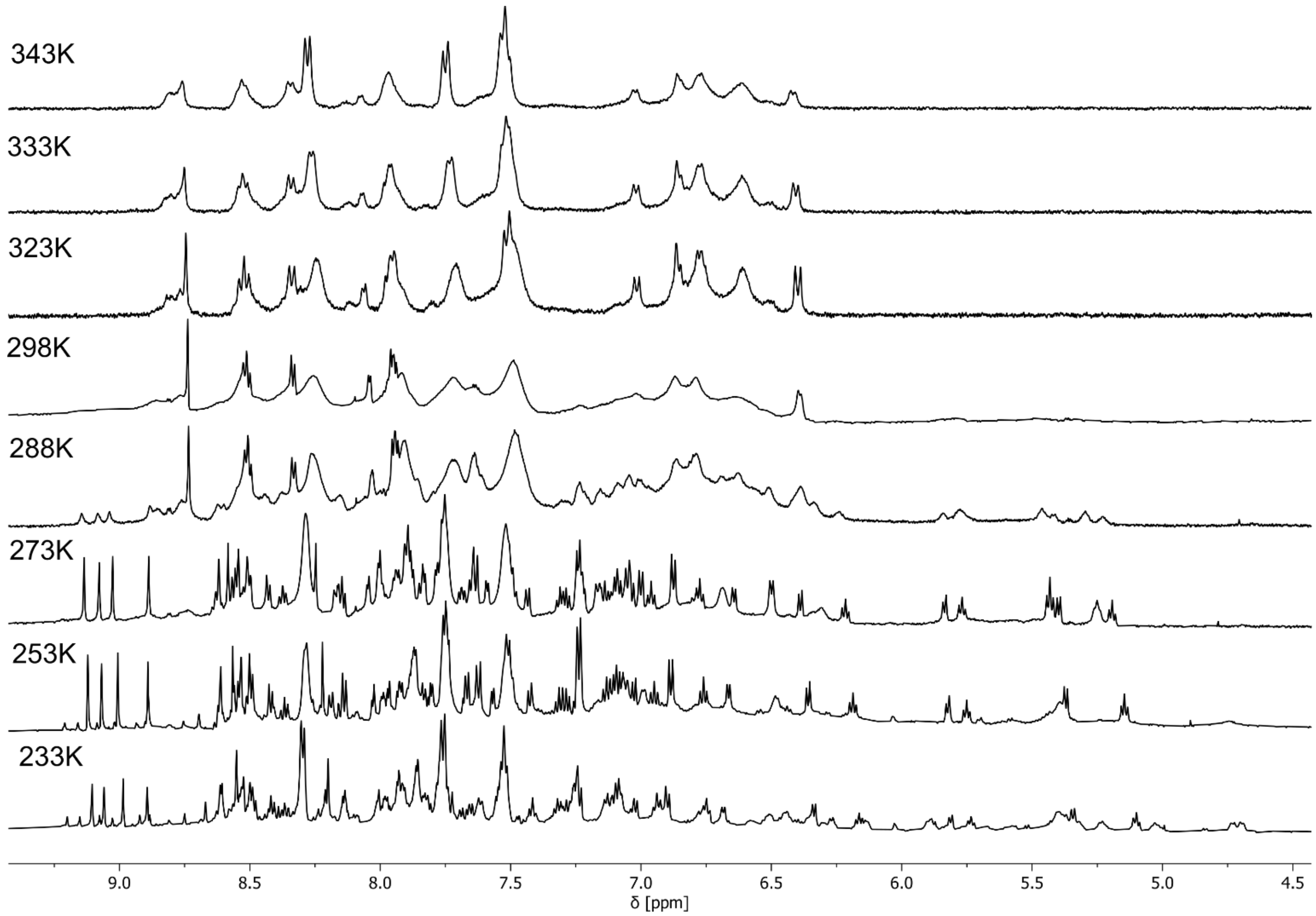

Figure S63. Variable-temperature ^{1}H NMR spectra of **4Zn** containing perylene (ca. 6 equiv.). CD_3CN, 600 MHz.

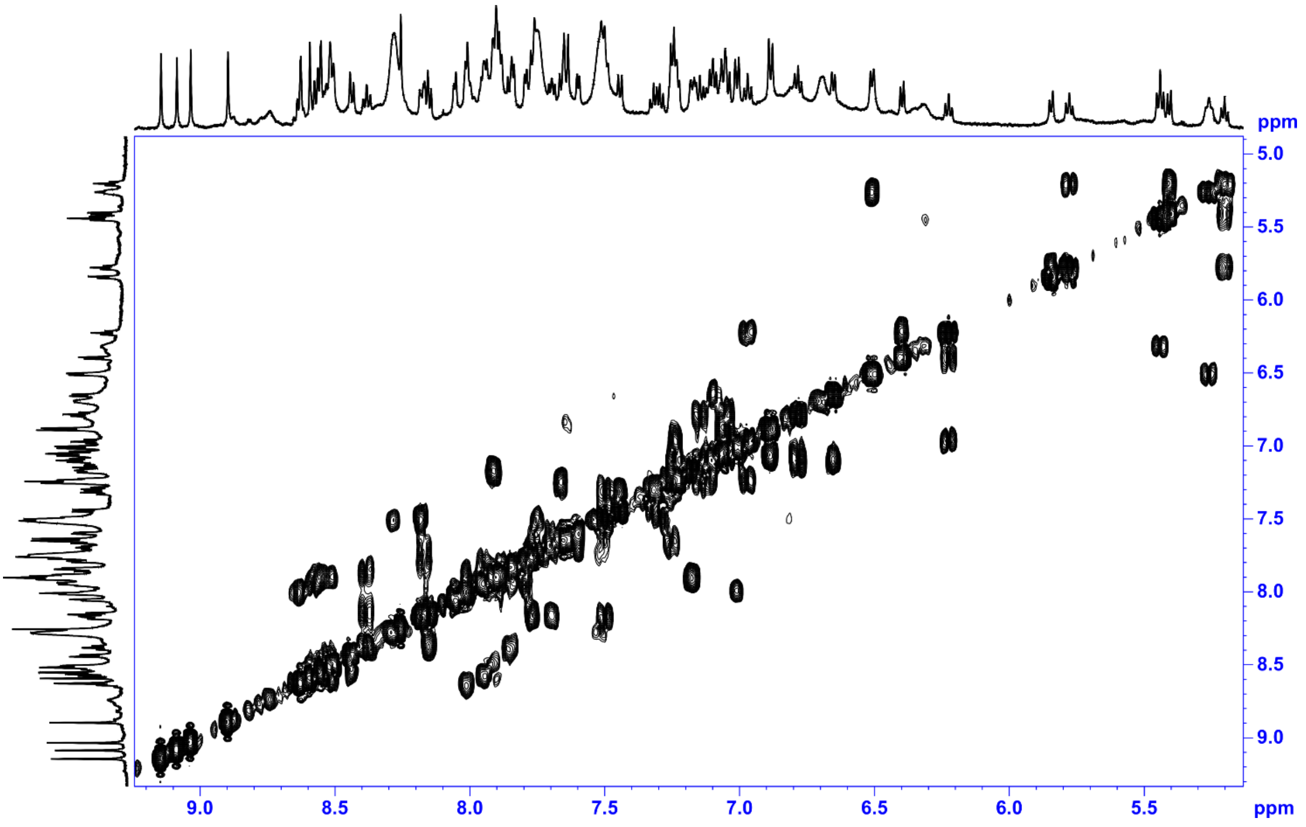

Figure S64. ^{1}H-^{1}H COSY NMR spectrum of **4Zn** with perylene (ca. 6 equiv.), CD_3CN, 273 K, 600 MHz.

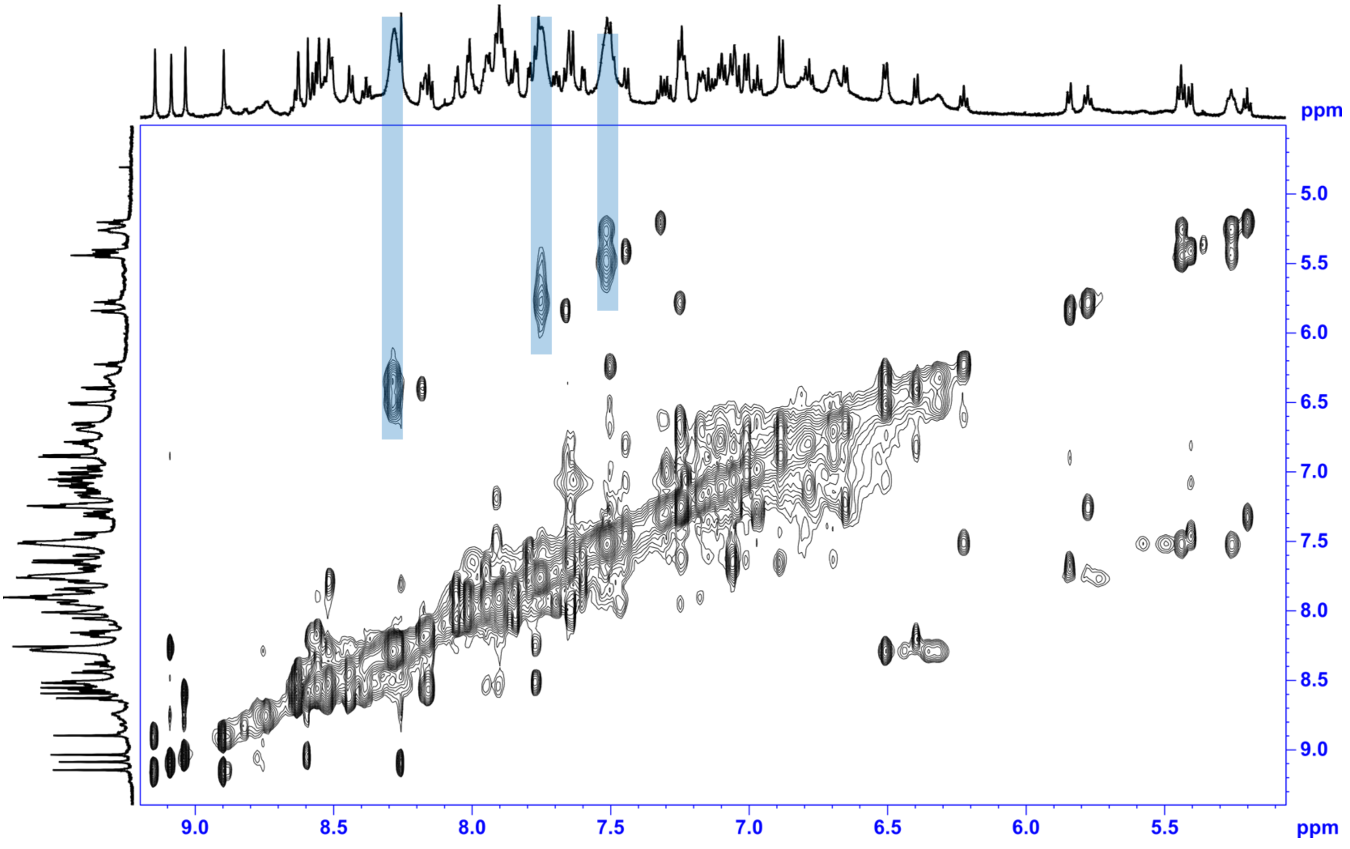

Figure S65. ^{1}H-^{1}H NOESY NMR spectrum of **4Zn** with perylene, CD_3CN, 273 K, 600 MHz, d_8 = 0.3 sec.

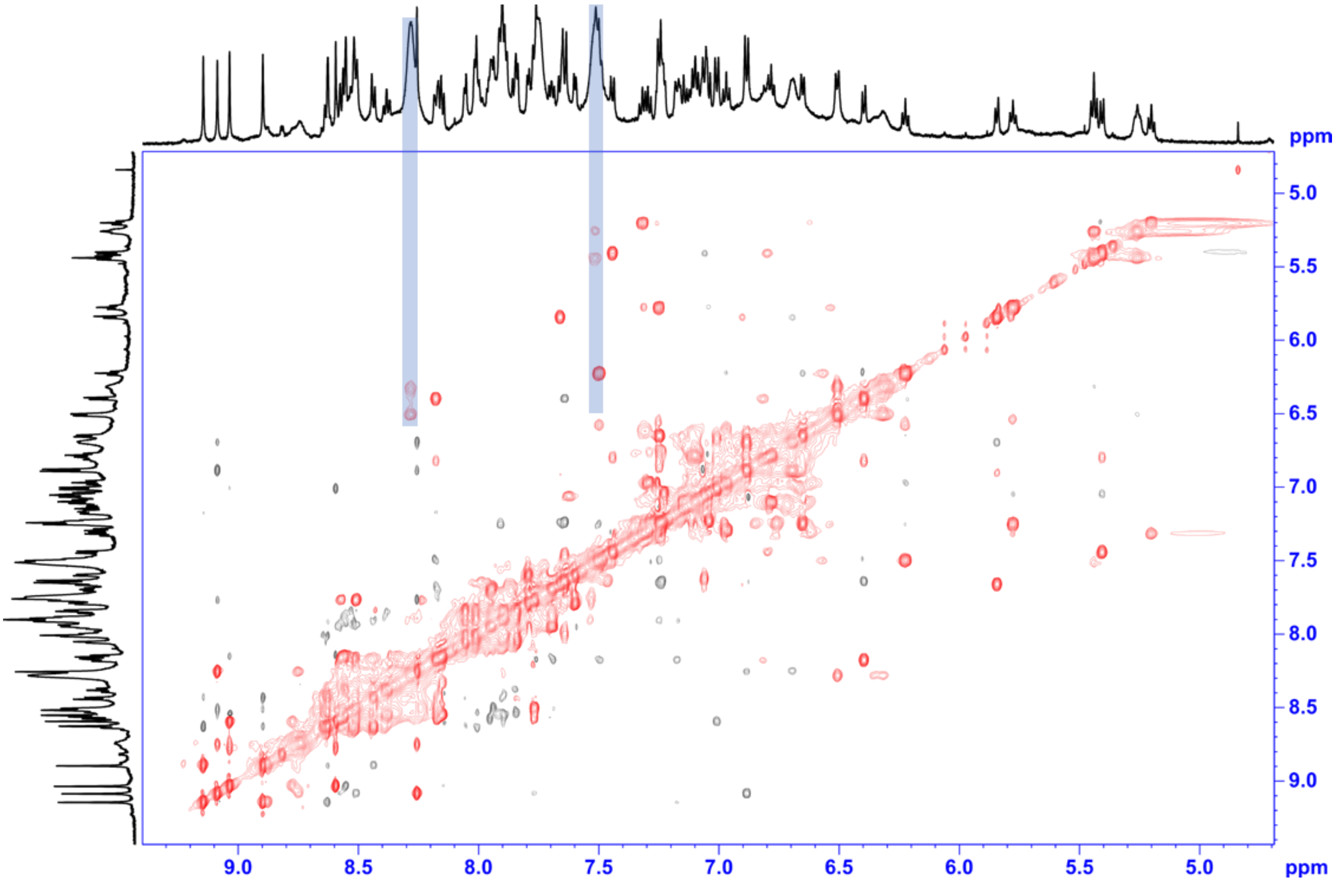

Figure S66. ^{1}H-^{1}H ROESY NMR spectrum of **4Zn** with perylene (ca. 6 equiv.), CD_3CN, 273 K, 600 MHz. Exchange correlations to the free guest molecules are highlighted in blue.

4.2.5. Cage 4Fe with corannulene

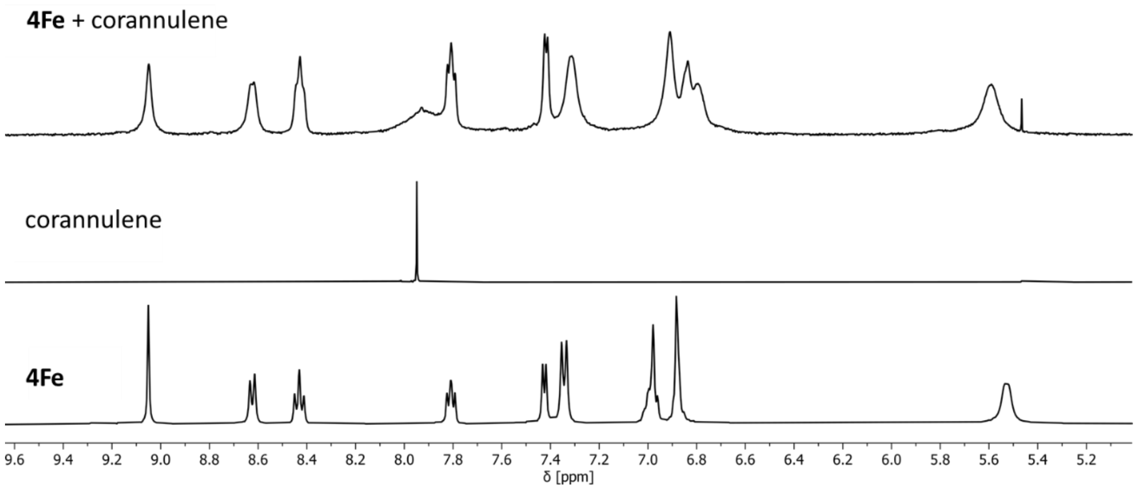

Figure S67. Comparison of ^{1}H NMR spectra of **4Fe**, corannulene and a mixture of **4Fe** with corannulene (ca. 8 equiv.). CD_3CN, 298 K, 400 MHz.

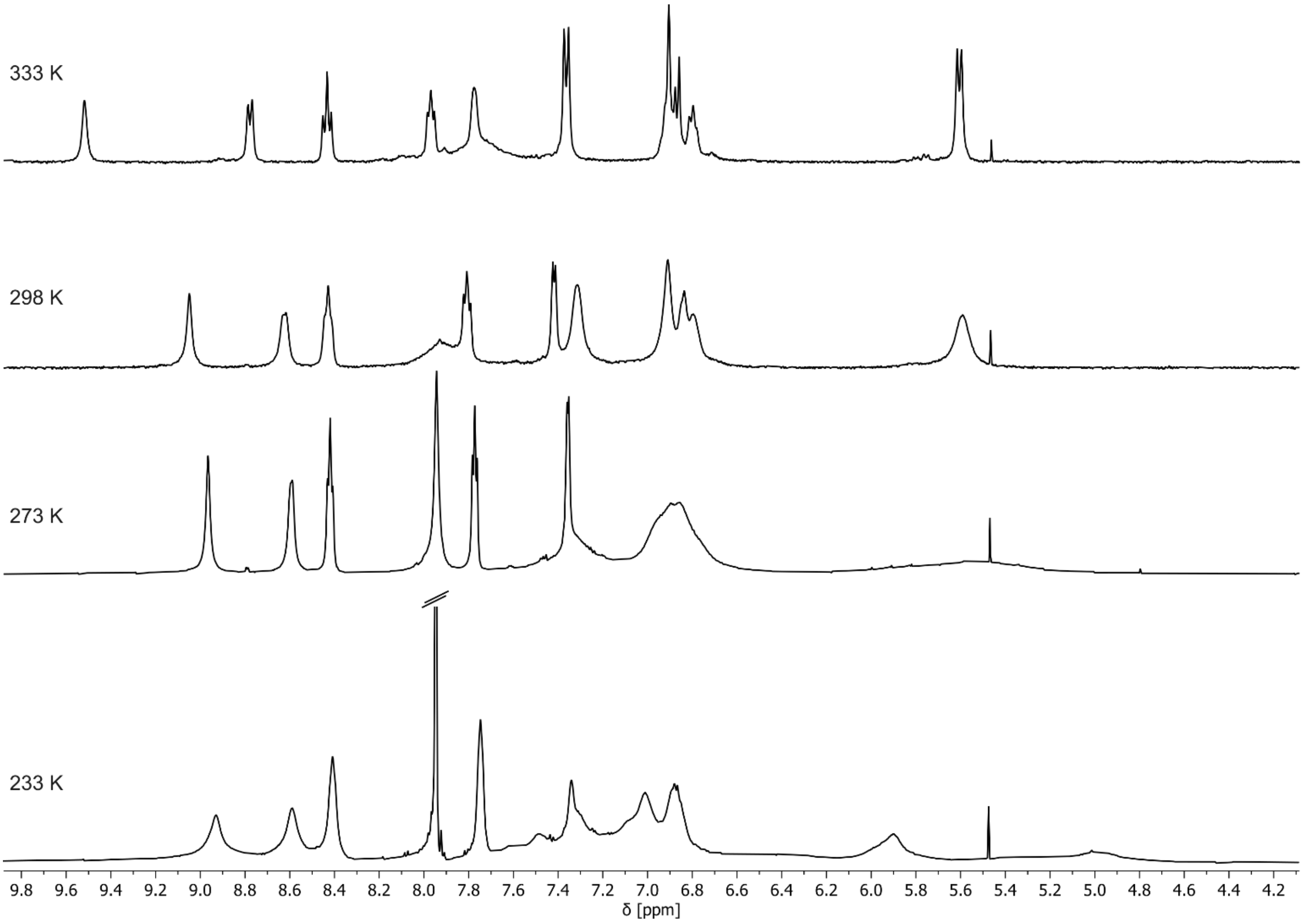

Figure S68. Variable-temperature ^1H NMR spectra of **4Fe** containing corannulene (ca. 8 equiv.). CD_3CN, 600 MHz.

4.2.6. Cage 4Zn with corannulene

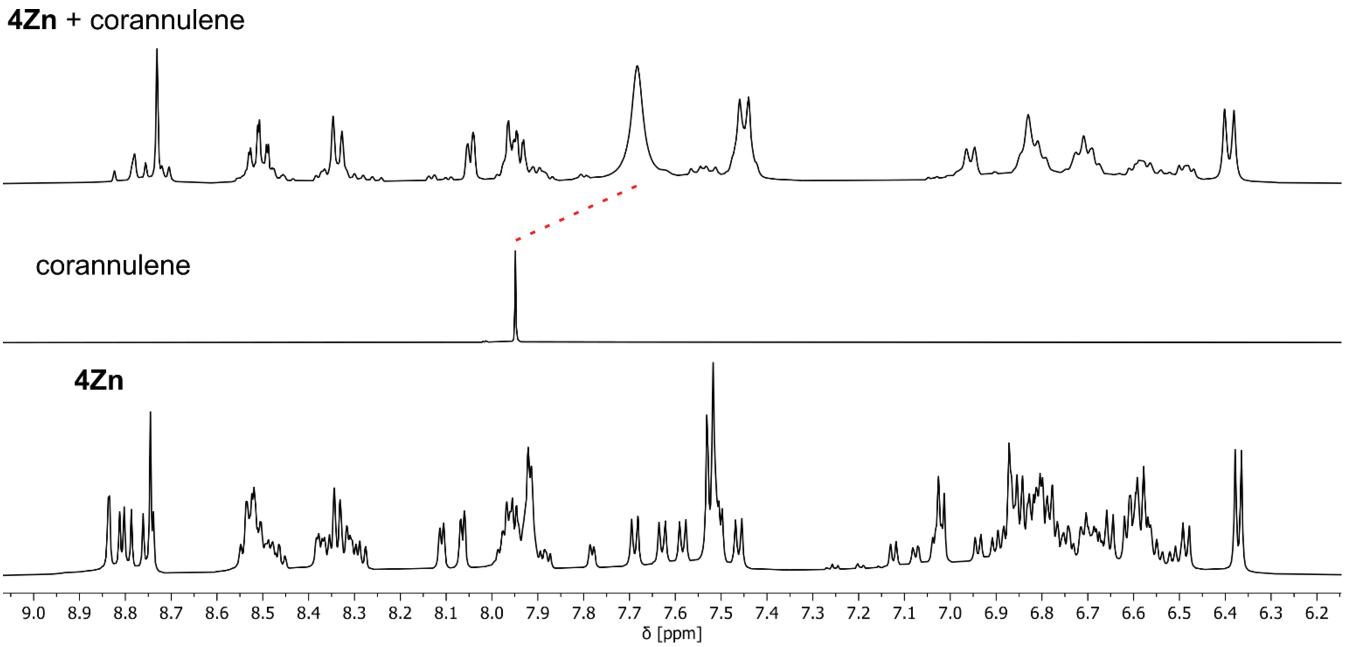

Figure S69. Comparison of ^{1}H NMR spectra of **4Zn**, corannulene and a mixture of **4Zn** with corannulene (ca. 8 equiv.). CD_3CN, 298 K, 400 MHz. The shift of the guest signal is denoted with a red dotted line.

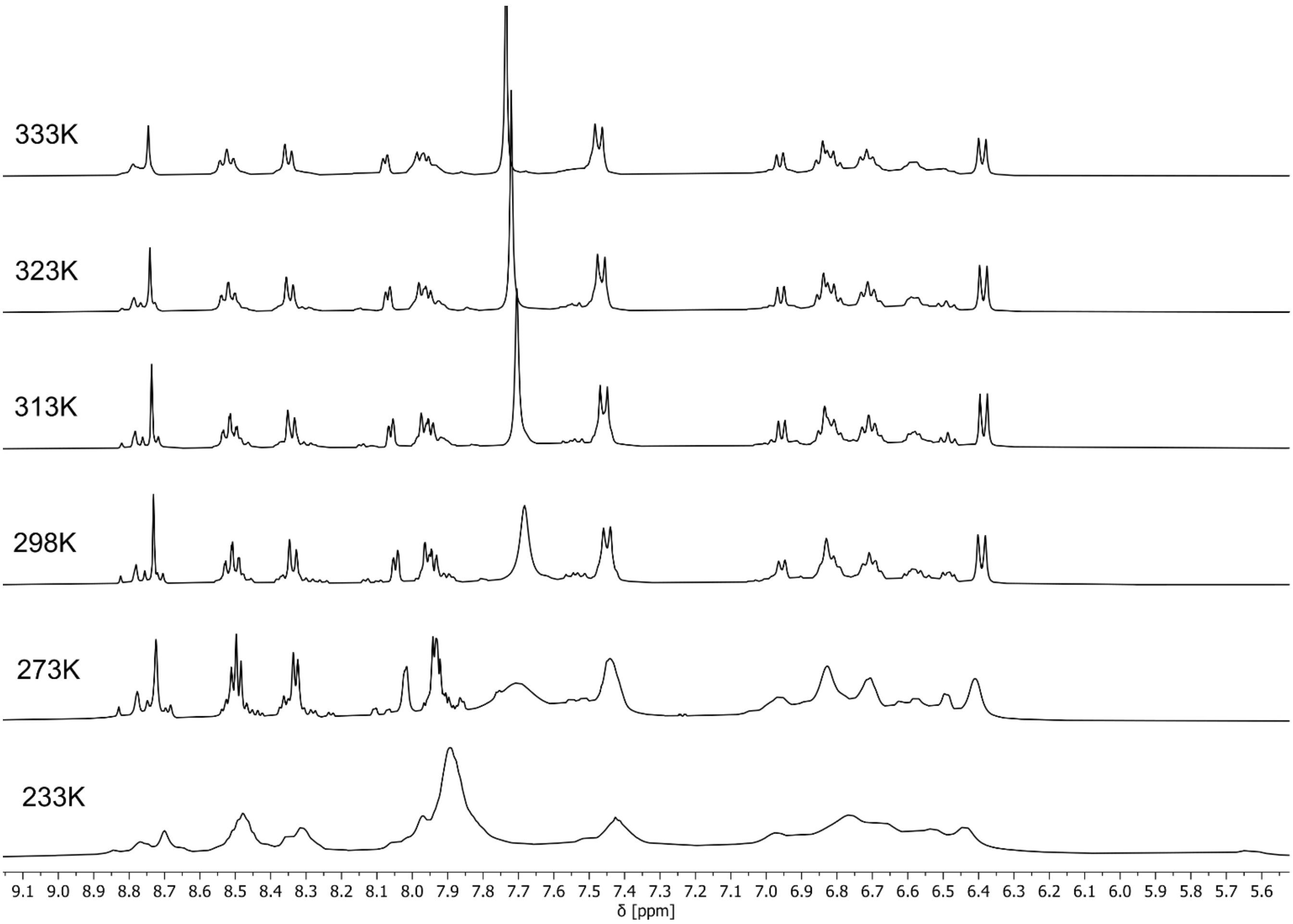

Figure S70. Variable-temperature ^{1}H NMR spectra of **4Zn** containing corannulene (ca. 8 equiv.). CD_3CN, 600 MHz.

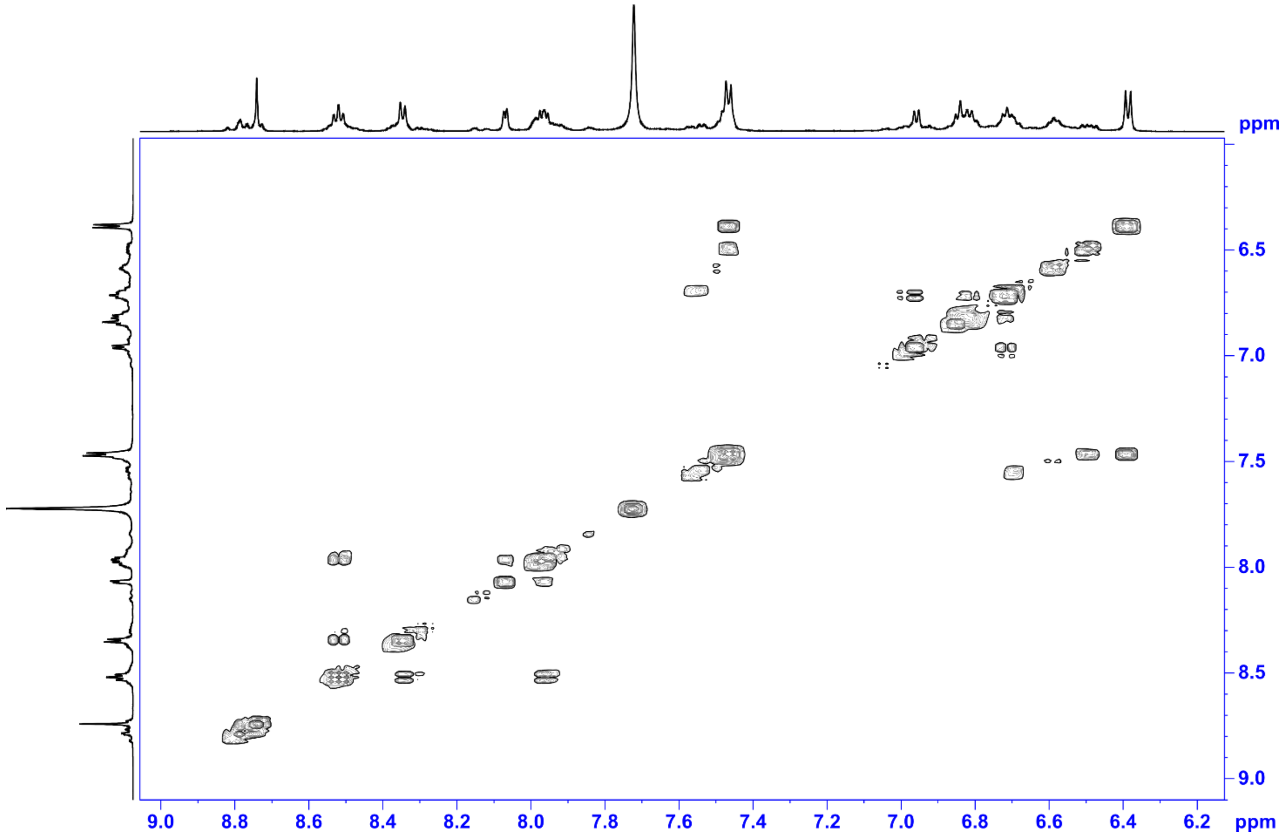

Figure S71. ^{1}H-^{1}H COSY NMR spectrum of **4Zn** with corannulene (ca. 8 equiv.), CD_3CN, 323 K, 600 MHz.

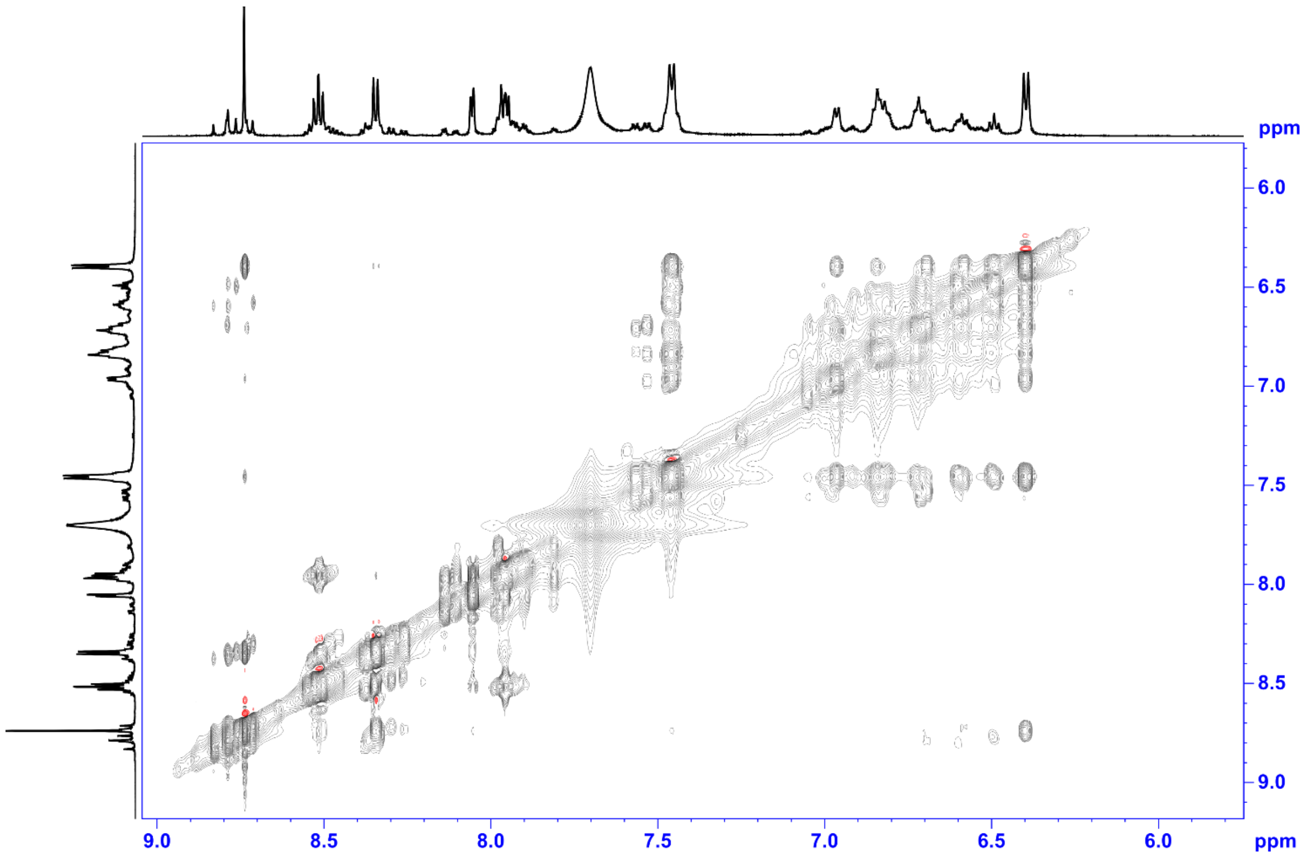

Figure S72. ^{1}H-^{1}H NOESY NMR spectrum of **4Zn** with corannulene (ca. 8 equiv.), CD_3CN, 298 K, 600 MHz. d_8 = 0.3 sec. No correlations to the corannulene signal are visible, consistent with fast guest exchange.

4.2.7. Cage 4Fe with coronene

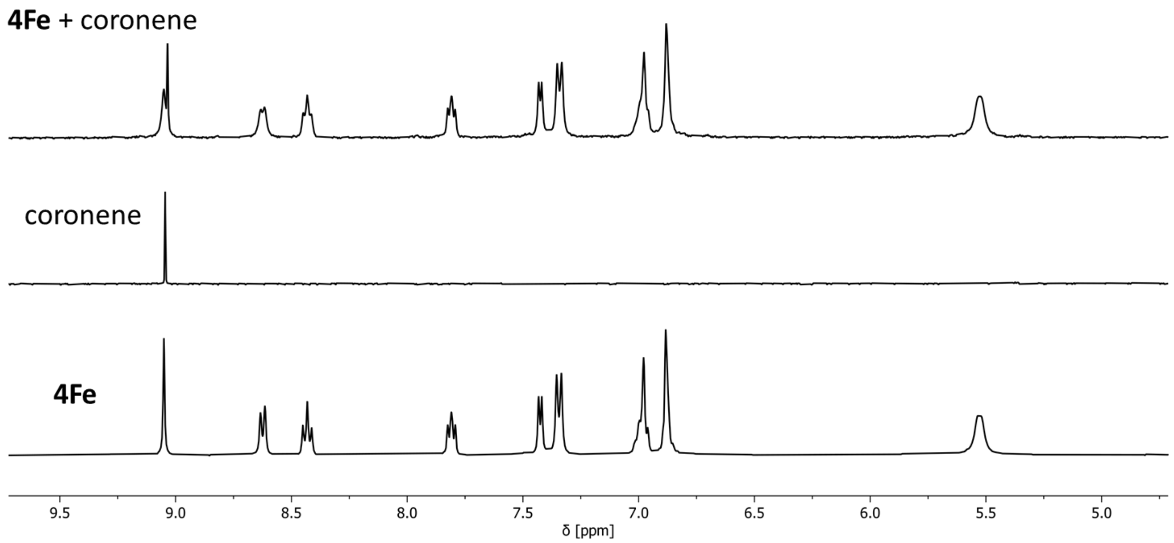

Figure S73. Comparison of ^{1}H NMR spectra of **4Fe**, coronene and a mixture of **4Fe** with coronene (ca. 8 equiv.). CD_3CN, 298 K, 400 MHz.

4.2.8. Cage 4Zn with coronene

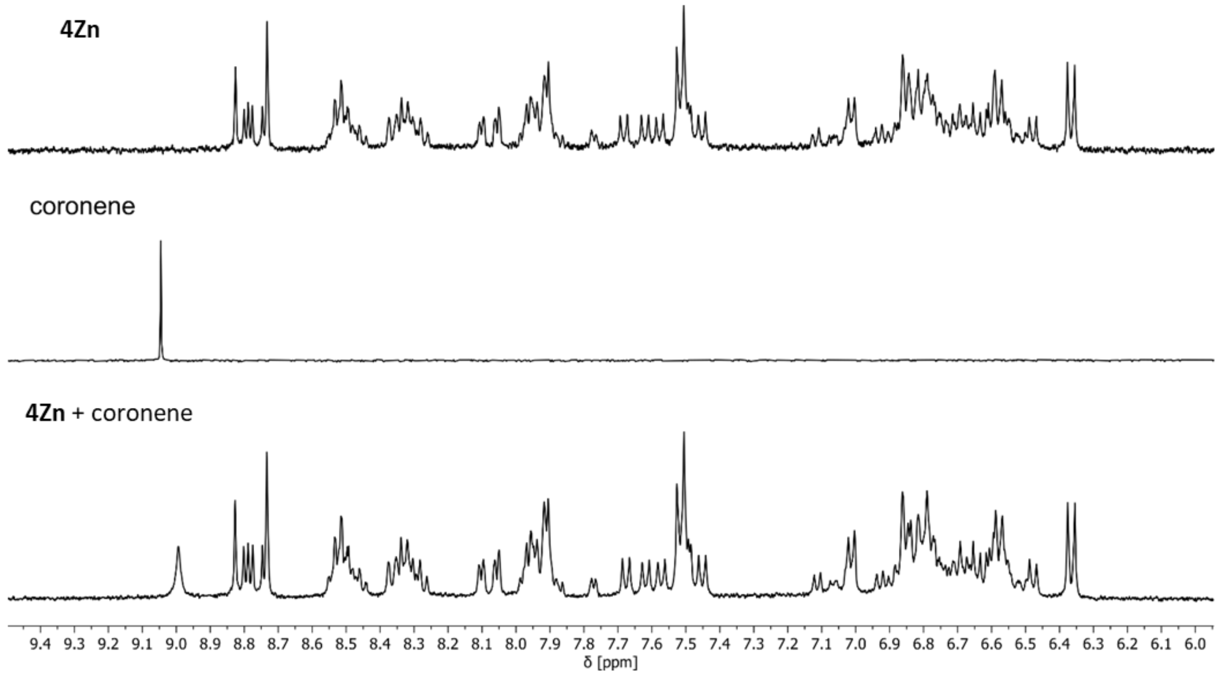

Figure S74. Comparison of ^{1}H NMR spectra of **4Zn**, coronene and a mixture of **4Zn** with coronene (ca. 8 equiv.). CD_3CN, 298 K, 400 MHz.

4.2.9. Cage 4Fe with anthracene

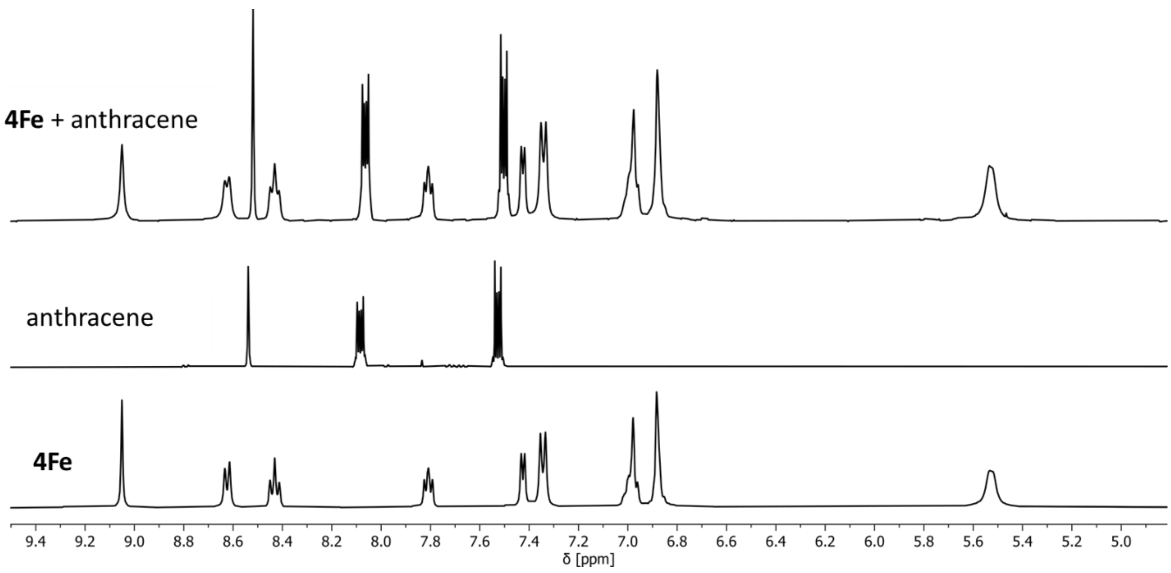

Figure S75. Comparison of ^{1}H NMR spectra of **4Fe**, anthracene and a mixture of **4Fe** with anthracene (ca. 8 equiv.). CD_3CN, 298 K, 400 MHz.

4.2.10. Cage 4Zn with anthracene

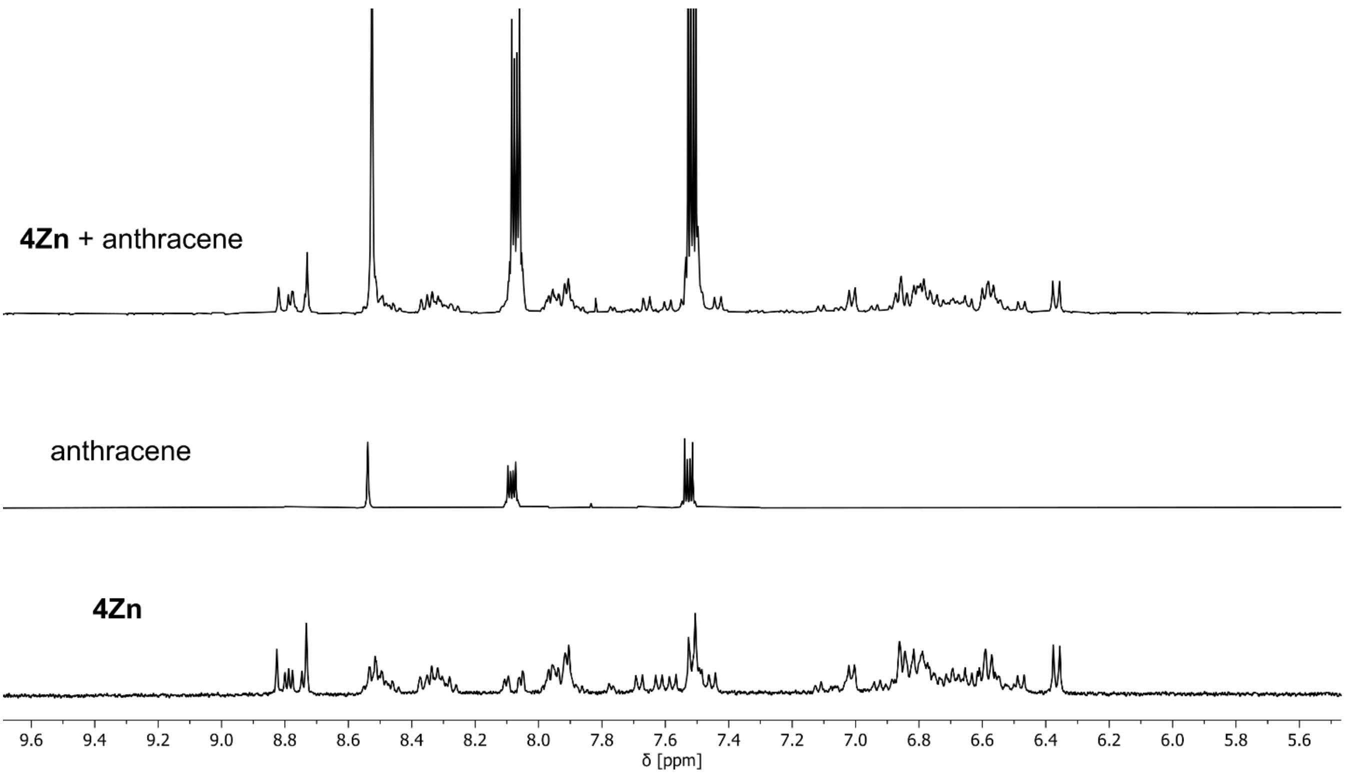

Figure S76. Comparison of ^{1}H NMR spectra of **4Zn**, anthracene and a mixture of **4Zn** with anthracene (ca. 10 equiv.). CD_3CN, 298 K, 400 MHz.

4.2.11. Cage 4Fe with anthraquinone

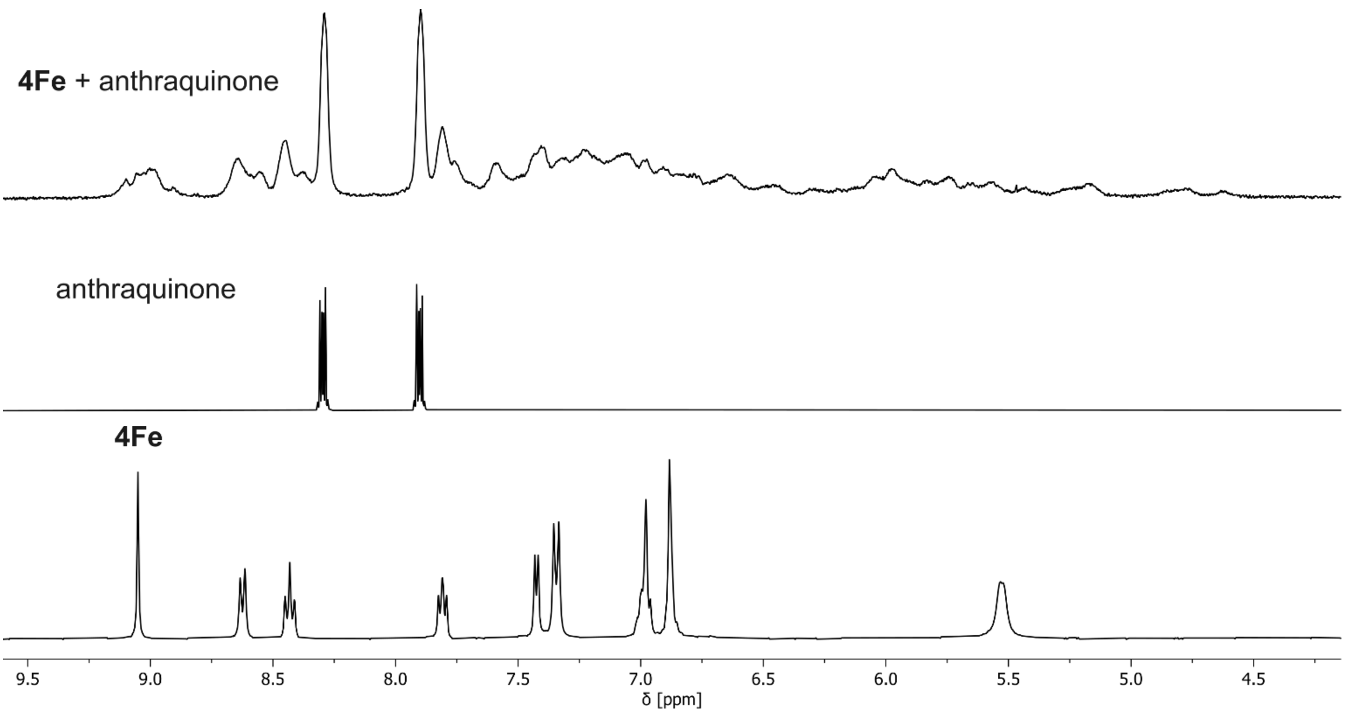

Figure S77. Comparison of ^{1}H NMR spectra of **4Fe**, anthraquinone and a mixture of **4Fe** with anthraquinone (ca. 8 equiv.) . CD_3CN, 298 K, 400 MHz.

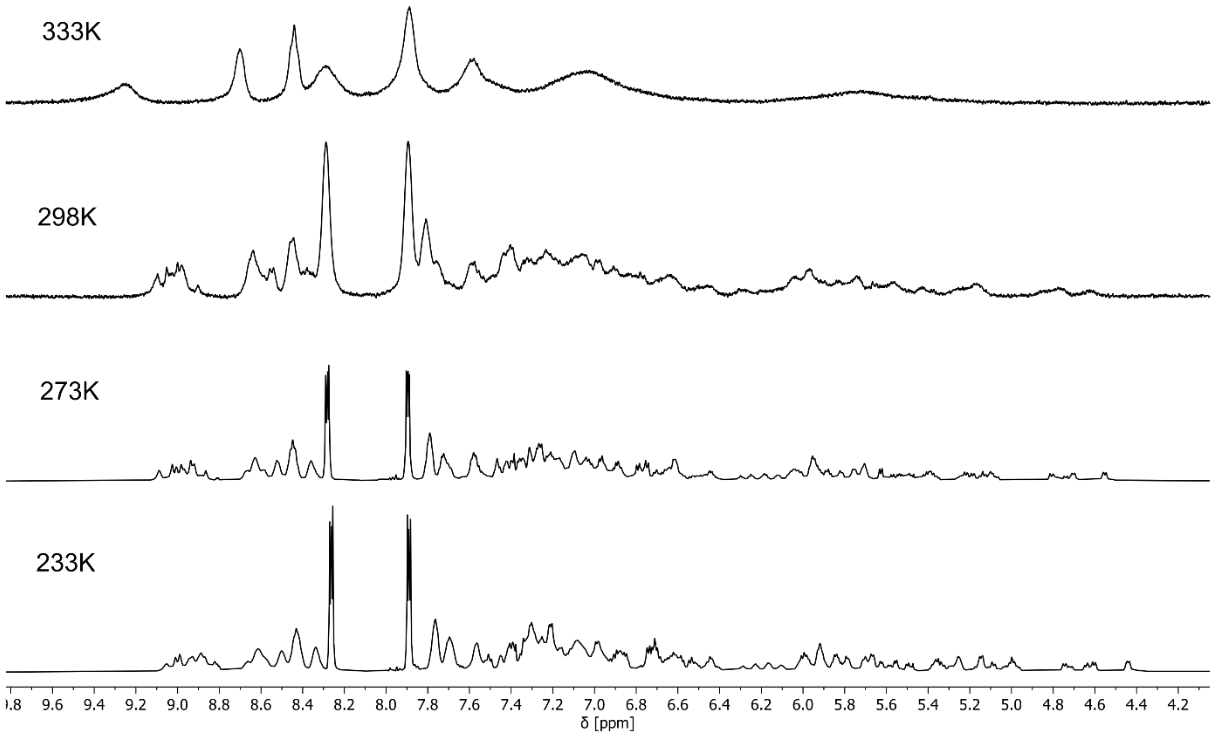

Figure S78. Variable-temperature ^{1}H NMR spectra of **4Fe** containing anthraquinone (ca. 8 equiv.). CD_3CN, 600 MHz.

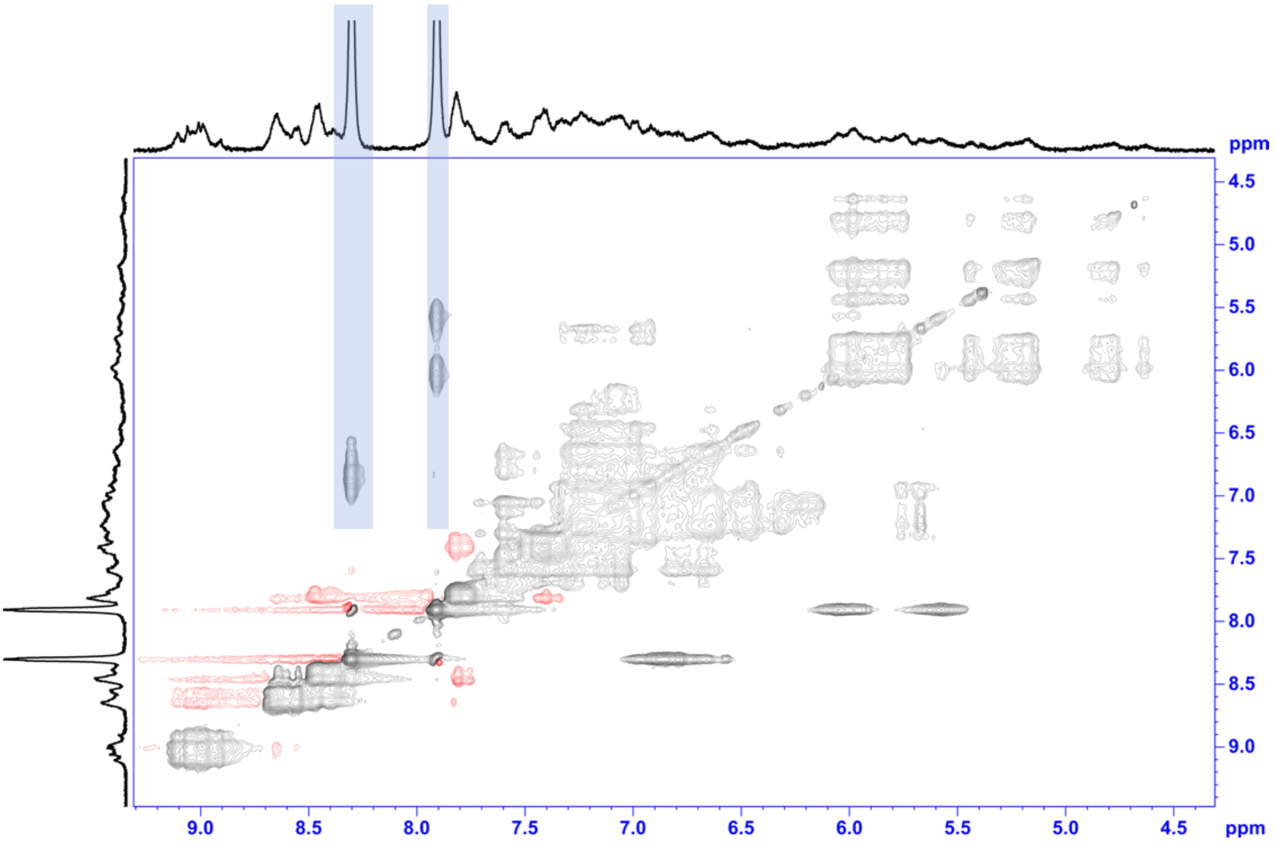

Figure S79. ^{1}H-^{1}H ROESY NMR spectrum of **4Fe** with anthraquinone (ca. 8 equiv.), CD_3CN, 298 K, 600 MHz. Exchange correlations to the free guest molecules are highlighted in blue.

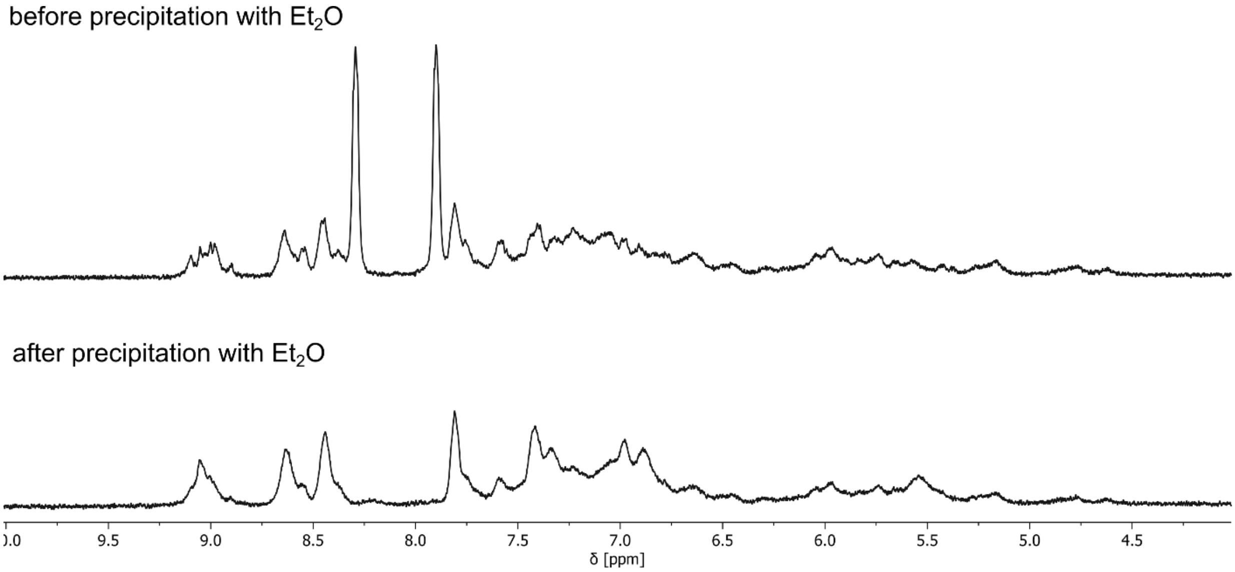

Figure S80. ^{1}H NMR spectra of **4Fe** with anthraquinone before (top) and after (bottom) precipitating the cage with diethyl ether, CD_3CN, 298 K, 400 MHz.

4.2.12. Cage 4Zn with anthraquinone

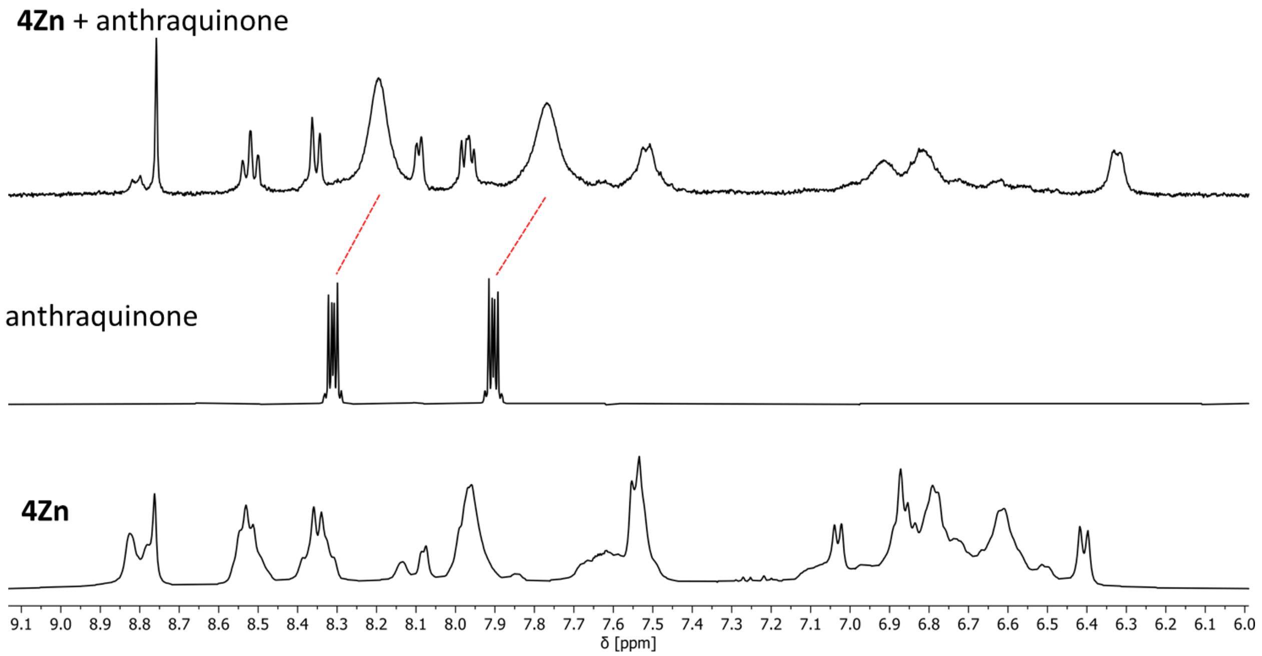

Figure S81. Comparison of ^{1}H NMR spectra of **4Zn**, anthraquinone and a mixture of **4Zn** with anthraquinone (ca. 4 equiv.). CD_3CN, 333 K, 400 MHz.

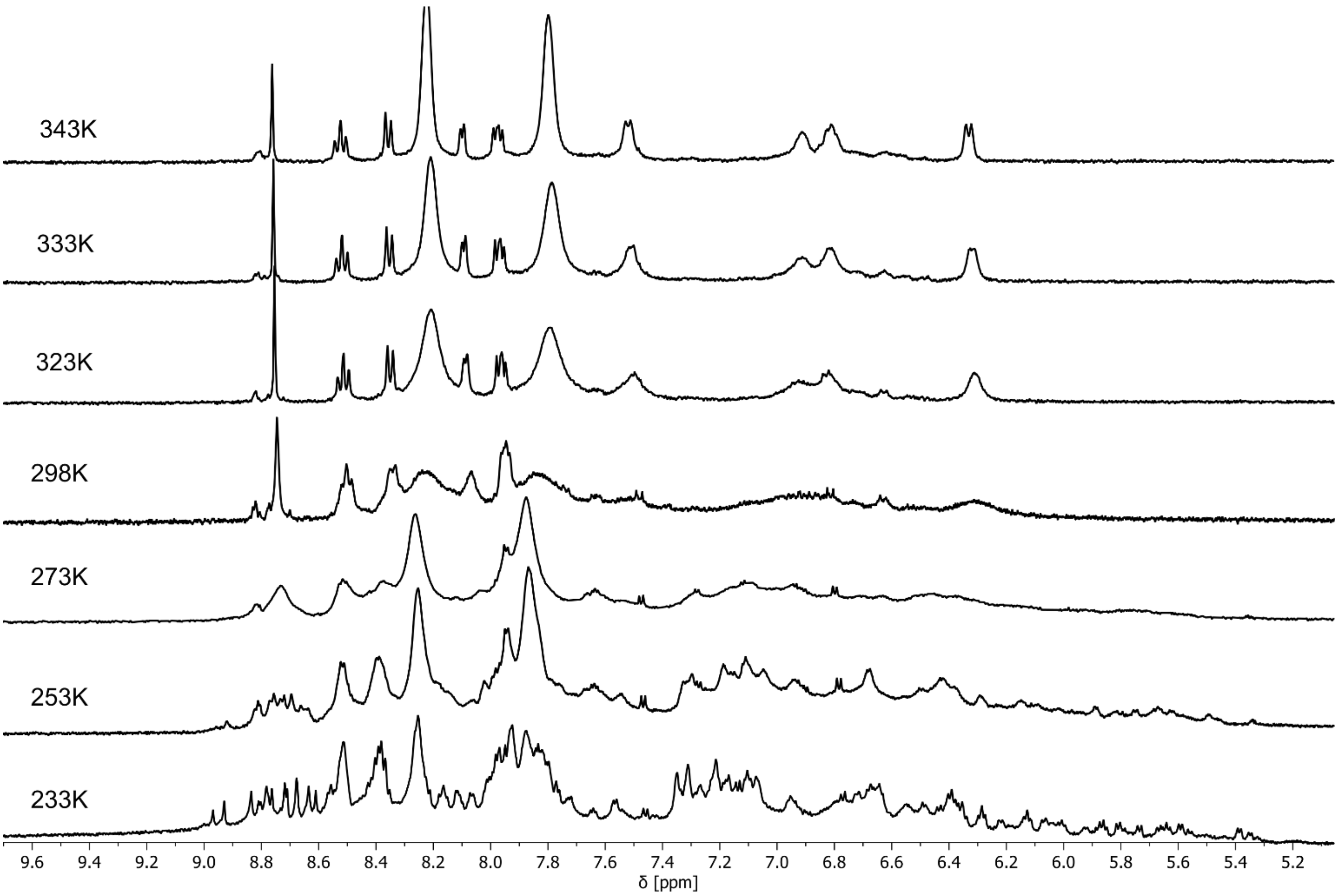

Figure S82. Variable-temperature ^{1}H NMR spectra of **4Zn** containing anthraquinone (ca. 4 equiv.). CD_3CN, 600 MHz.

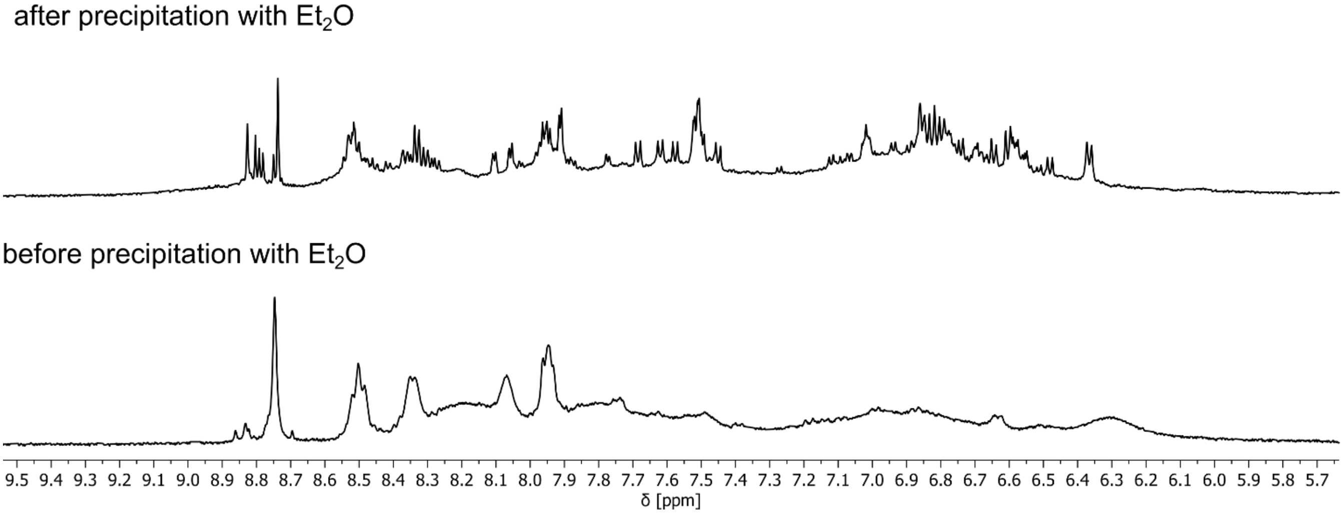

Figure S83. ^{1}H NMR spectra of **4Zn** with anthraquinone before (bottom) and after (top) precipitating the cage with diethyl ether, CD_3CN, 298 K, 400 MHz.

4.2.13. Cage 4Fe with 9,10-dihydroxyanthracene (AQ-H_2)

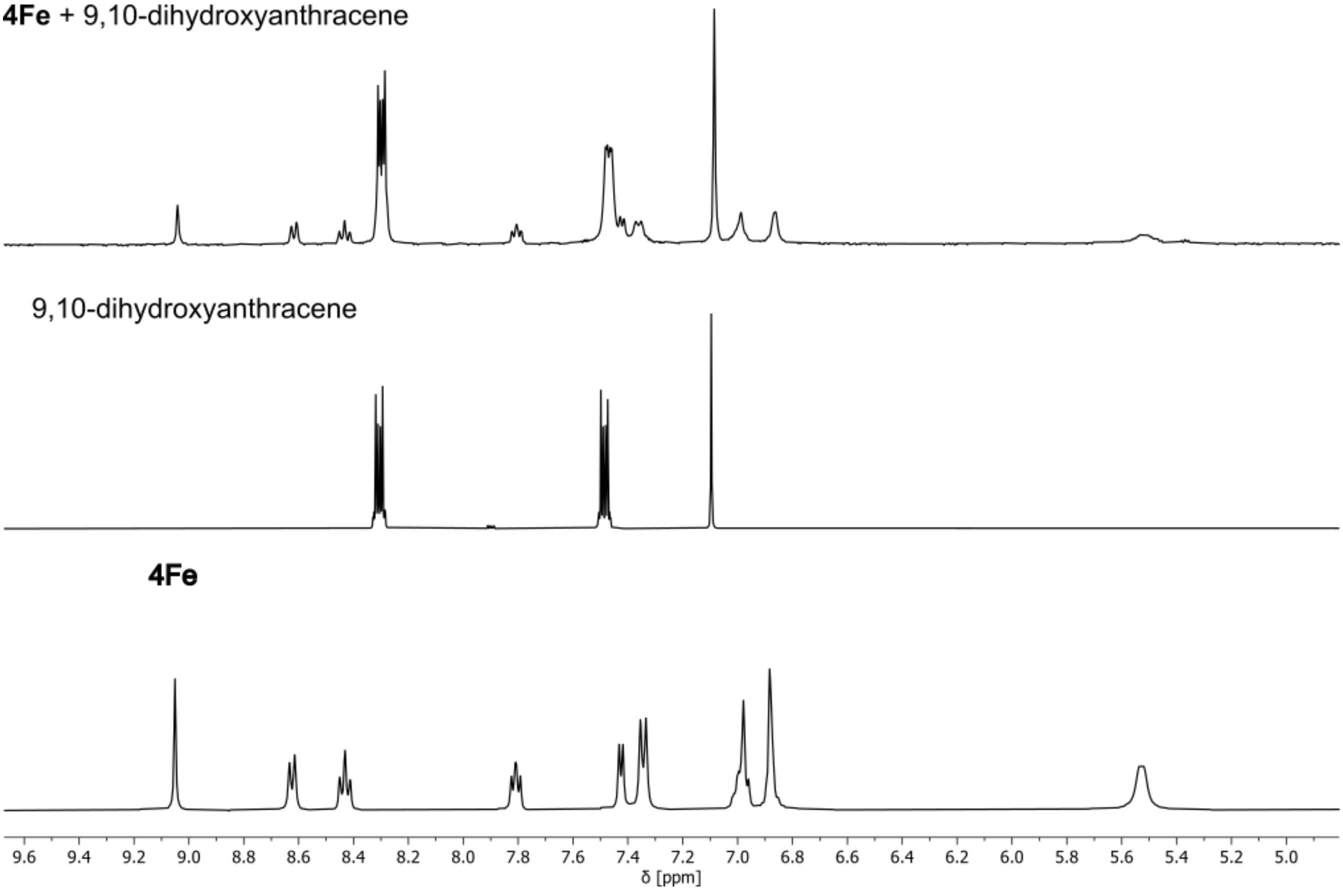

Figure S84. Comparison of ^{1}H NMR spectra of **4Fe**, 9,10-dihydroxyanthracene and a mixture of **4Fe** with 9,10-dihydroxyanthracene (ca. 8 equiv.). CD_3CN, 298 K, 400 MHz. The samples containing 9,10-dihydroxyanthracene were prepared with dry, degassed CD_3CN inside a glovebox and sealed in a J-Young NMR tube before measurement.

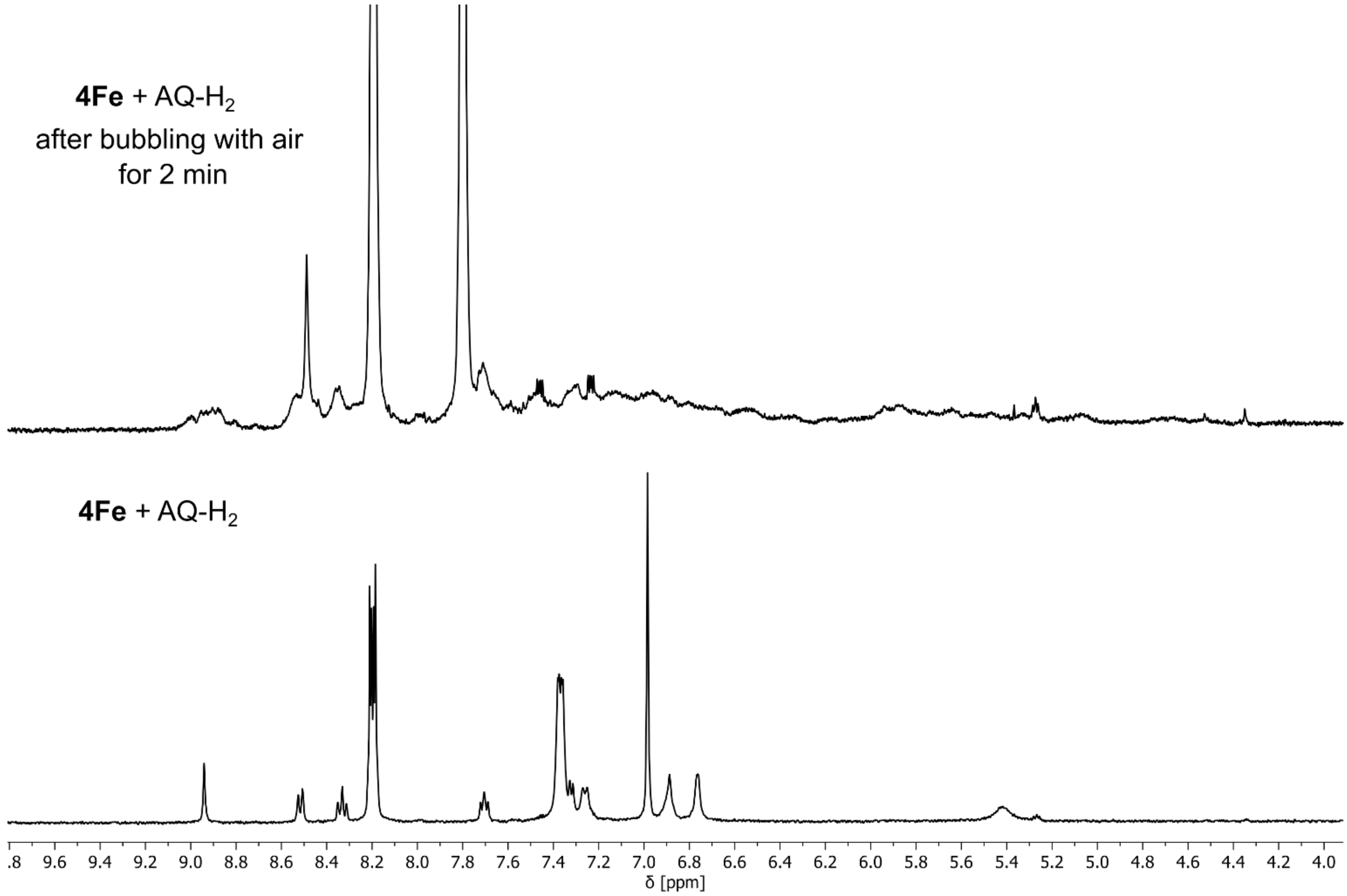

Figure S85. Comparison of ^{1}H NMR spectra of **4Fe +** 9,10-dihydroxyanthracene before (bottom) and after (top) bubbling with air for 2 min. The spectrum indicates full conversion to anthraquinone and binding of the resulting anthraquinone to the cage. CD_3CN, 298 K, 400 MHz.

4.2.14. Cage 4Zn with 9,10-dihydroxyanthracene (AQ-H_2)

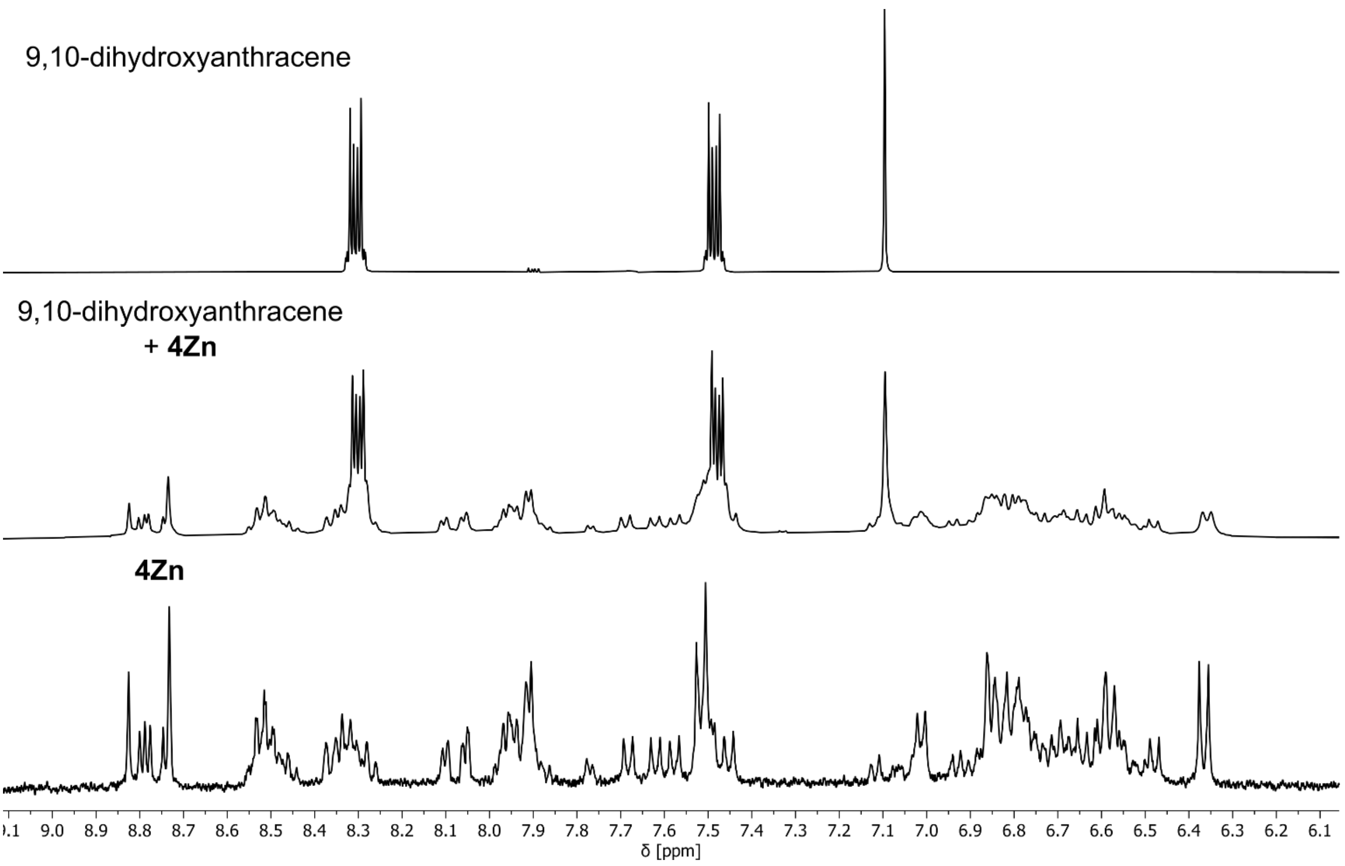

Figure S86. Comparison of ^{1}H NMR spectra of **4Zn**, 9,10-dihydroxyanthracene and a mixture of **4Zn** with 9,10-dihydroxyanthracene (ca. 8 equiv.). CD_3CN, 298 K, 400 MHz. The samples containing 9,10-dihydroxyanthracene were prepared with dry, degassed CD_3CN inside a glovebox and sealed in a J-Young NMR tube before measurement.

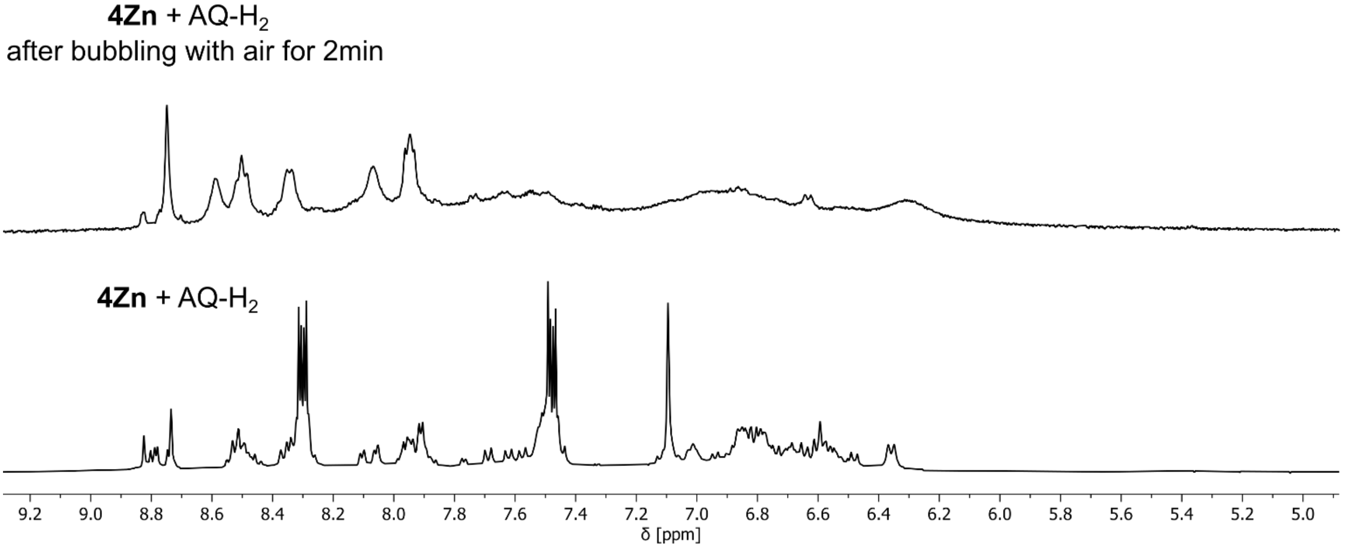

Figure S87. Comparison of ^{1}H NMR spectra of **4Zn +** 9,10-dihydroxyanthracene before (bottom) and after (top) bubbling with air for 2 min. The spectrum indicates full conversion to anthraquinone and binding of the resulting anthraquinone to the cage. CD_3CN, 298 K, 400 MHz.

4.2.15. Cage 4Fe with testosterone

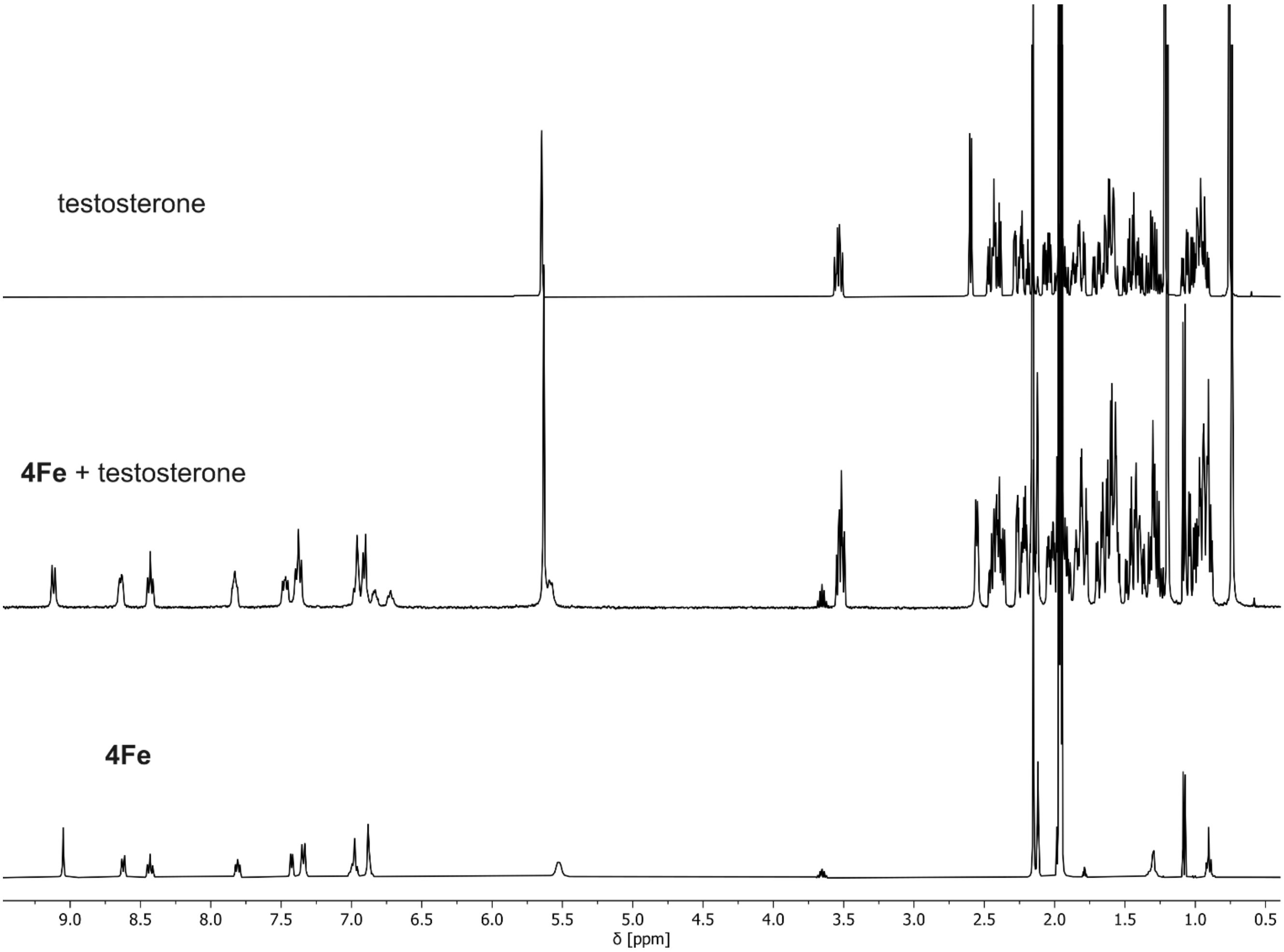

Figure S88. Comparison of ^{1}H NMR spectra of **4Fe**, testosterone and a mixture of **4Fe** with testosterone (ca. 6 equiv.). CD_3CN, 298 K, 400 MHz.

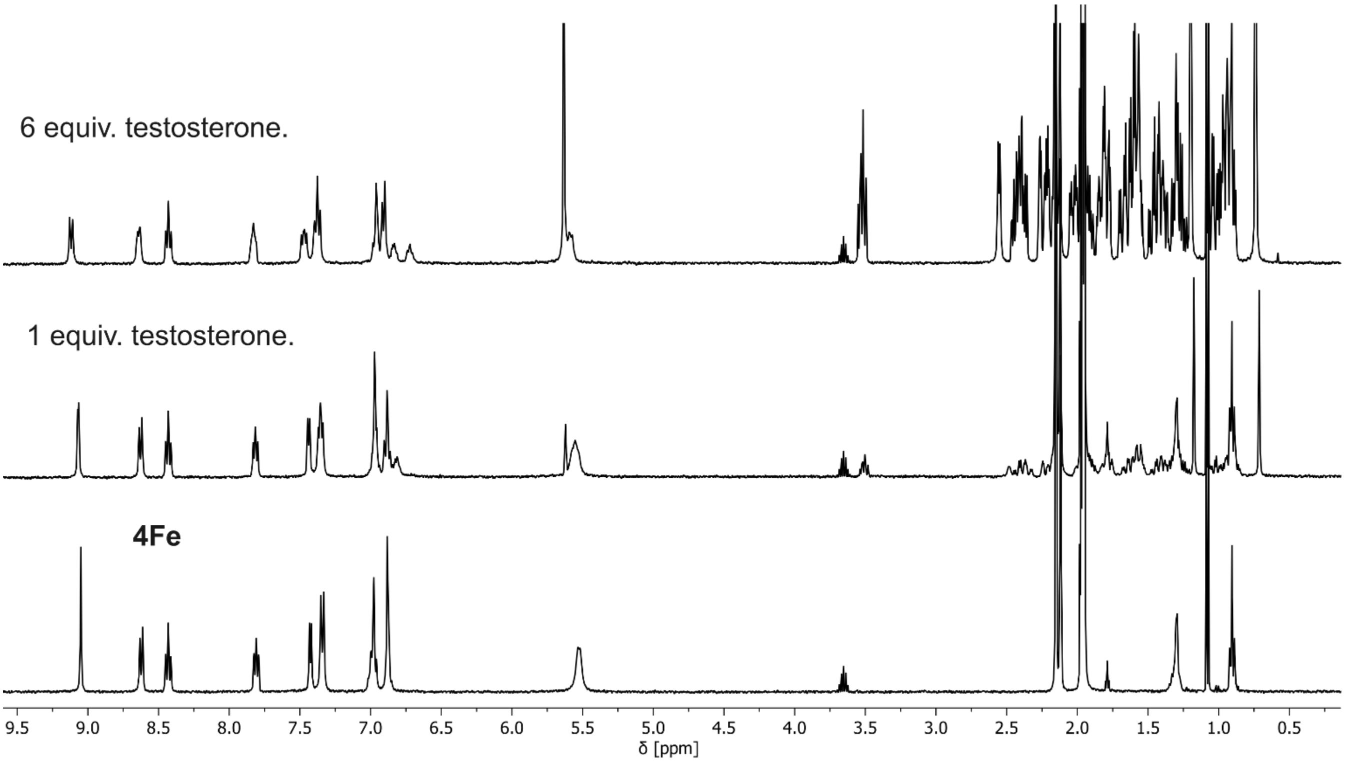

Figure S89. Comparison of ^{1}H NMR spectra of **4Fe** containing different amounts of testosterone. CD_3CN, 298 K, 400 MHz.

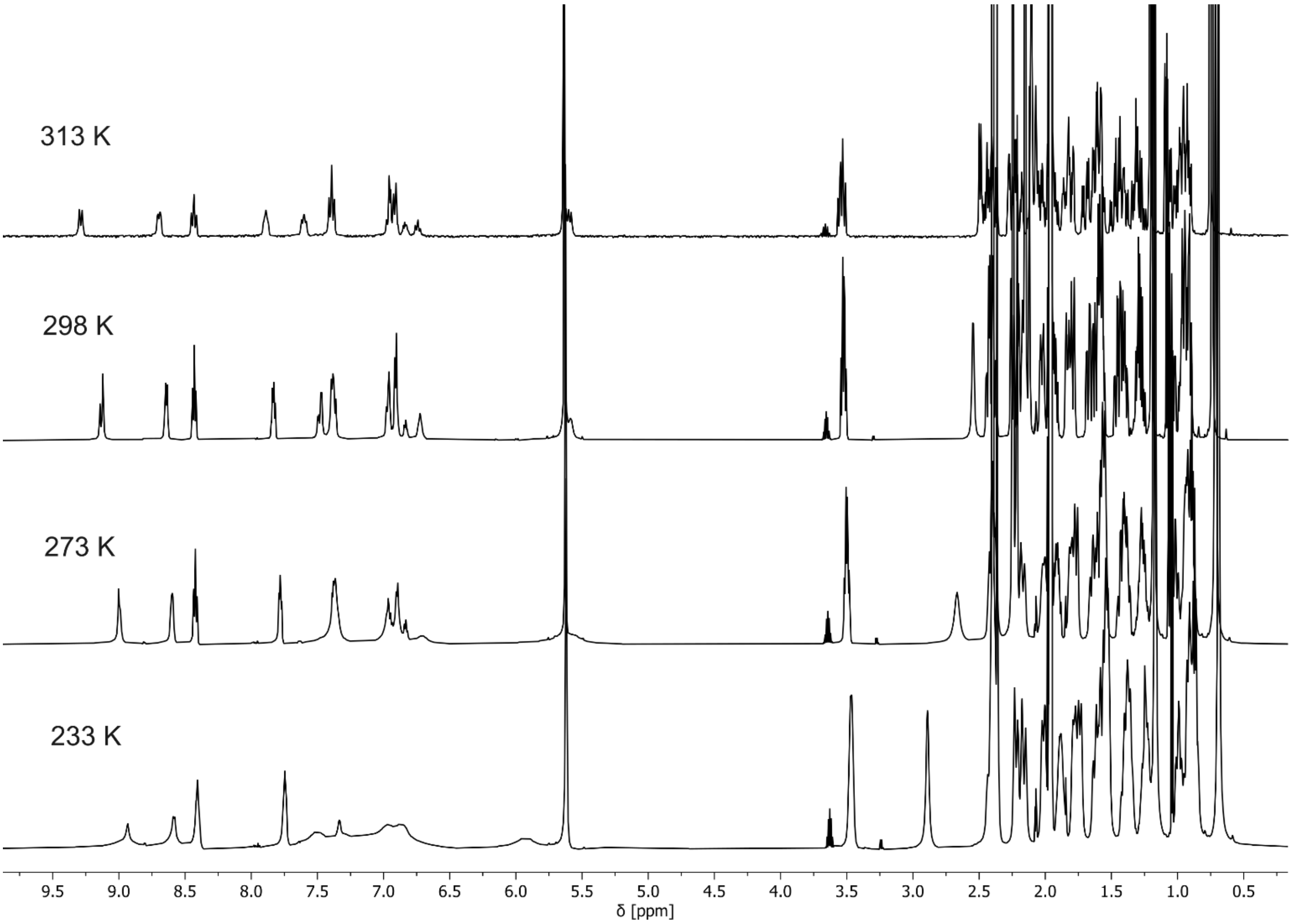

Figure S90. Variable-temperature ^{1}H NMR spectra of **4Fe** containing testosterone (ca. 6 equiv.). CD_3CN, 600 MHz.

4.2.16. Cage 4Zn with testosterone

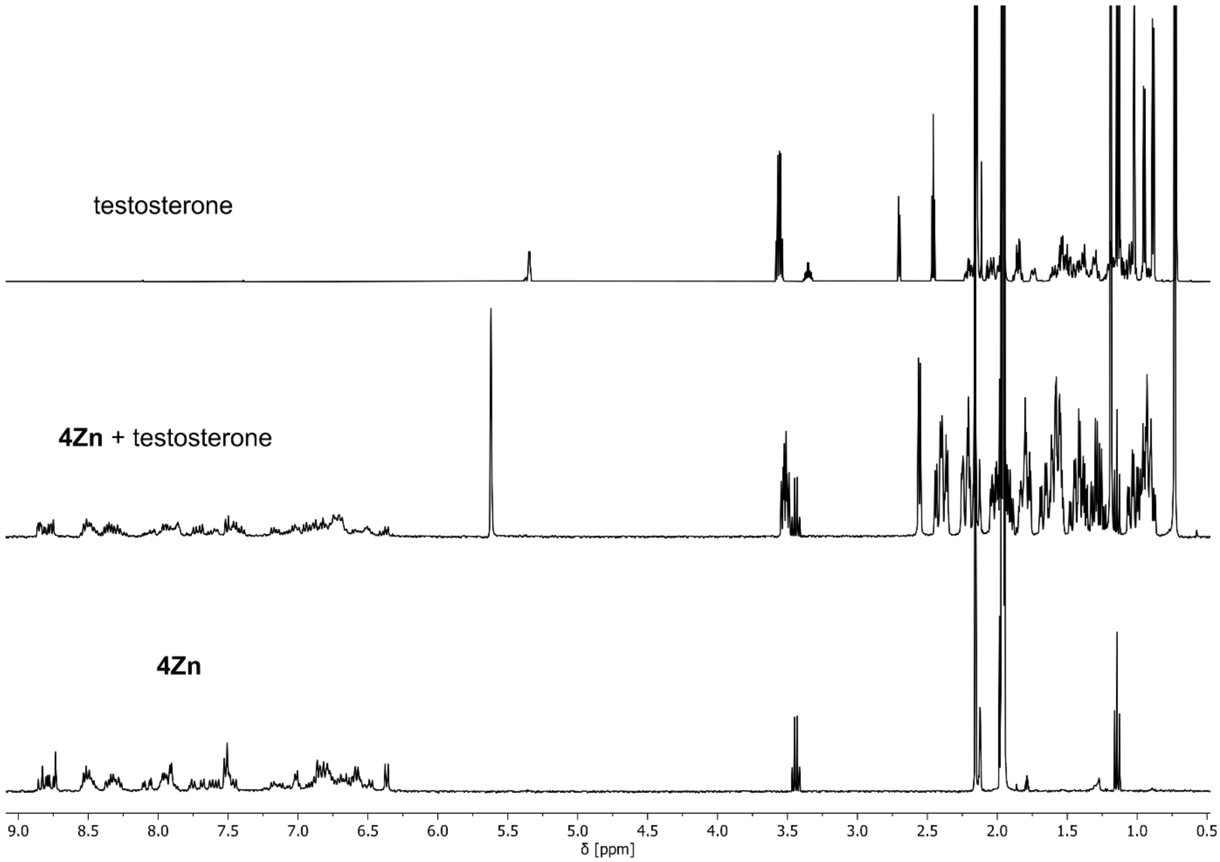

Figure S91. Comparison of ^{1}H NMR spectra of **4Zn**, testosterone and a mixture of **4Zn** with testosterone (ca. 10 equiv.). CD_3CN, 298 K, 400 MHz.

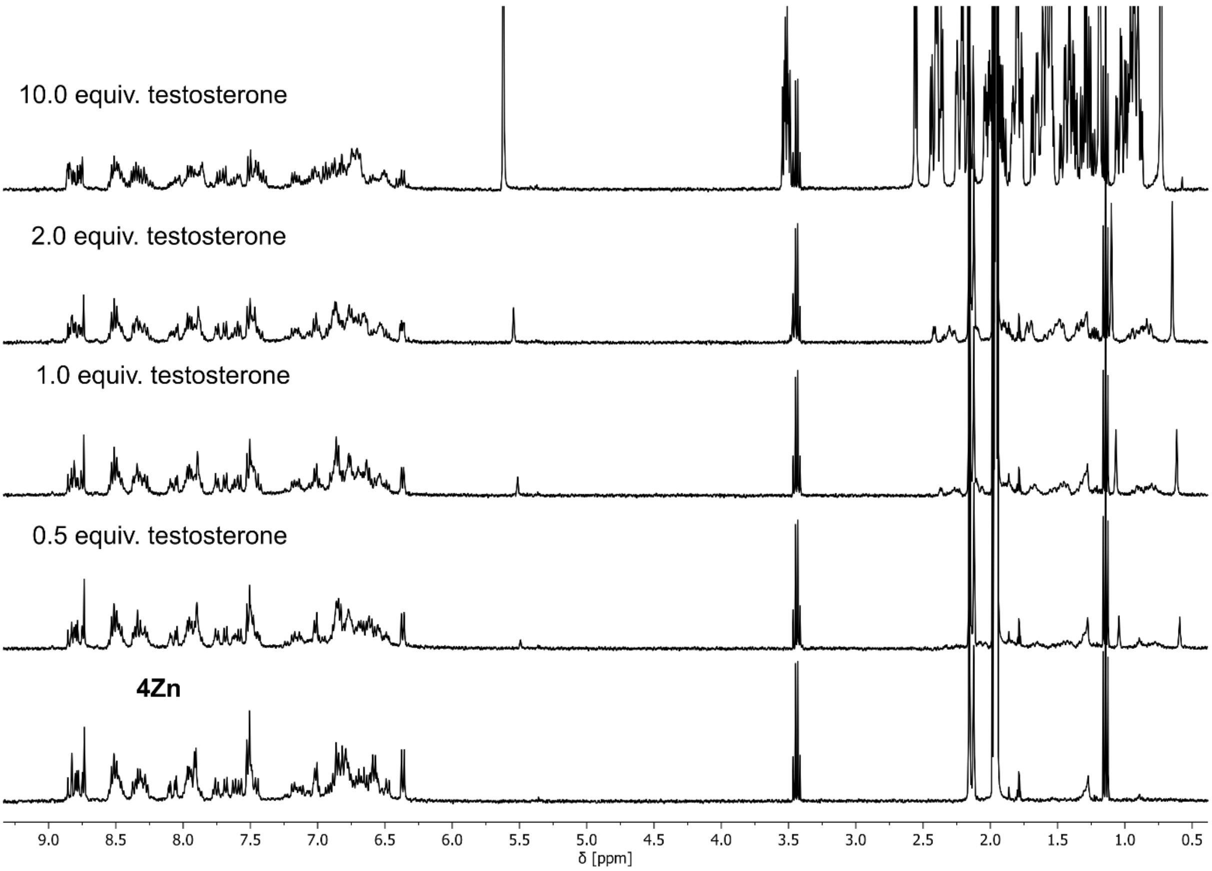

Figure S92. Comparison of ^{1}H NMR spectra of **4Zn** containing different amounts of testosterone (ca. 10 equiv.). CD_3CN, 298 K, 400 MHz.

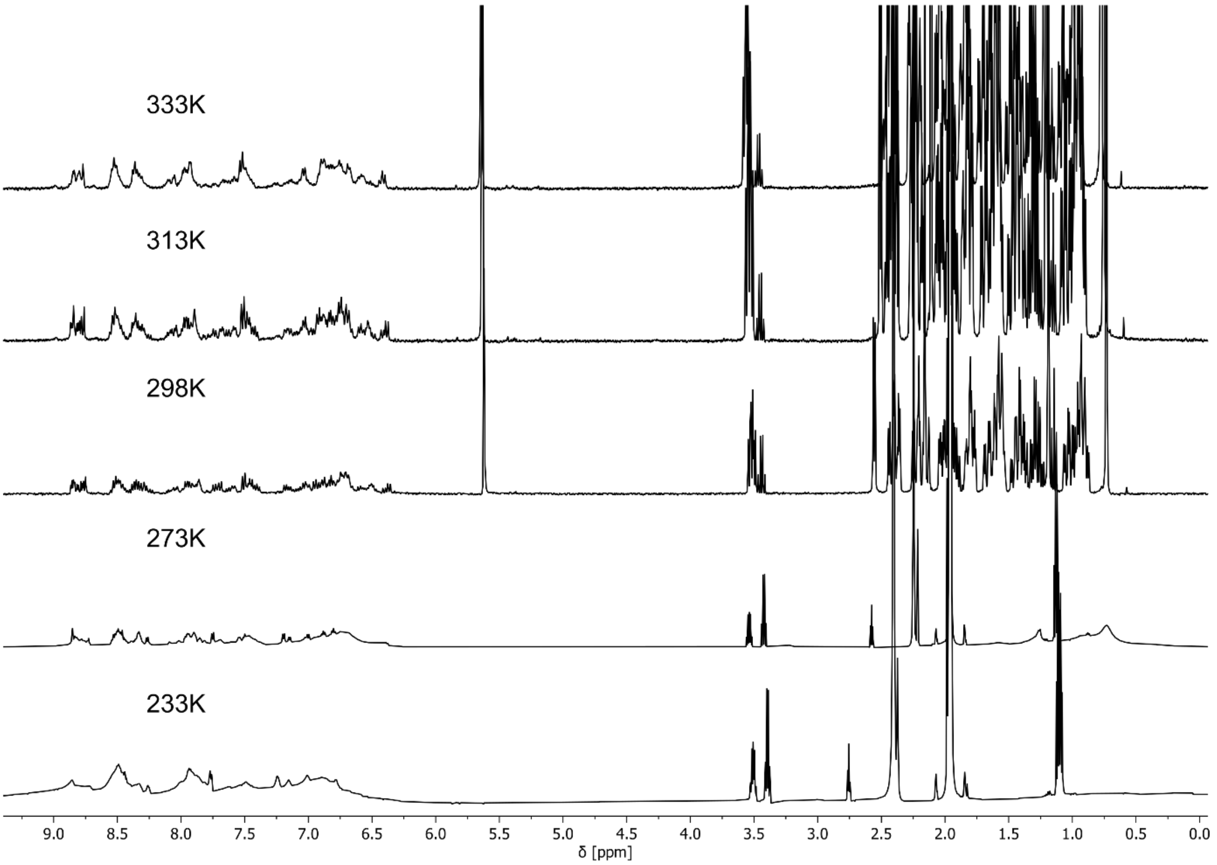

Figure S93. Variable-temperature ^{1}H NMR spectra of **4Zn** containing testosterone (ca. 10 equiv.). CD_3CN, 600 MHz.

4.2.17. Cage 4Fe with cholesterol

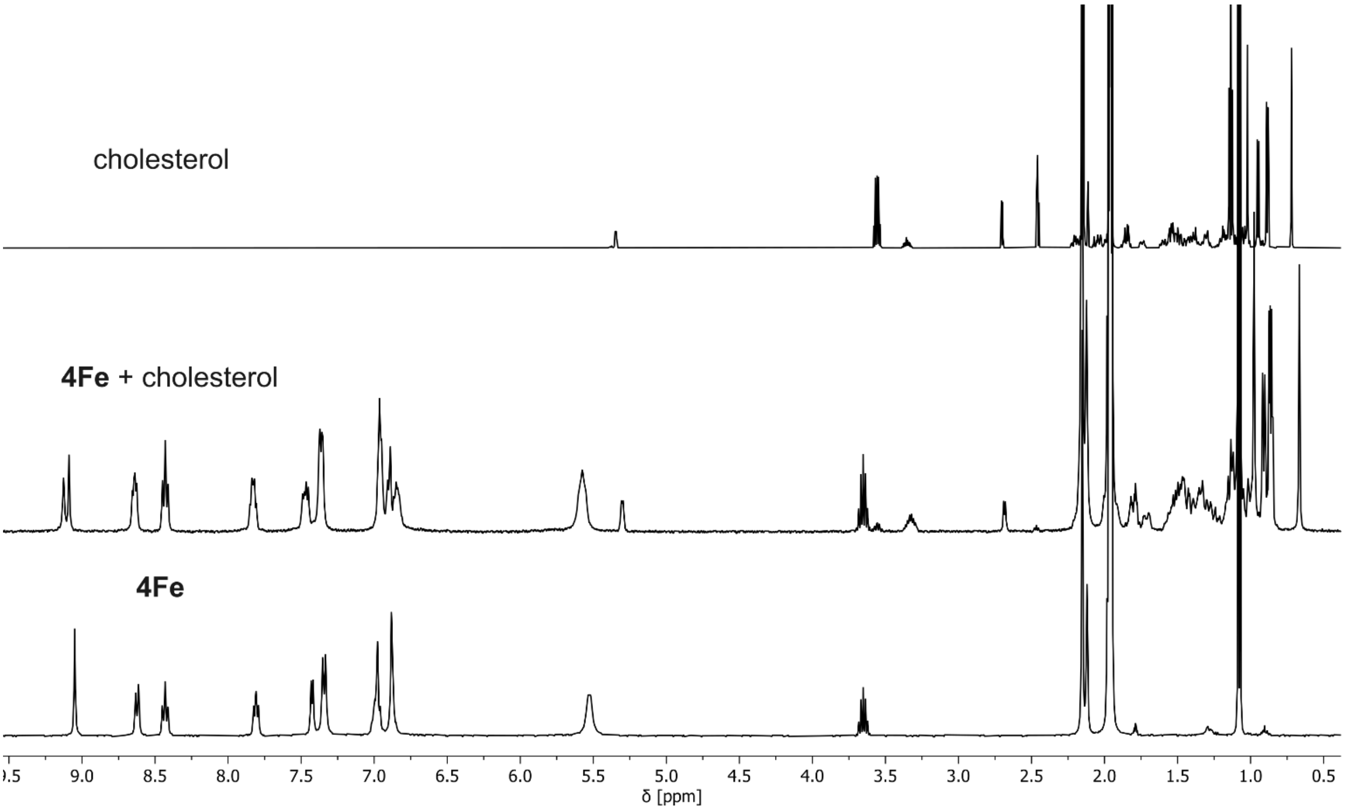

Figure S94. Comparison of ^{1}H NMR spectra of **4Fe**, cholesterol and a mixture of **4Fe** with cholesterol (ca. 8 equiv.). CD_3CN, 298 K, 400 MHz.

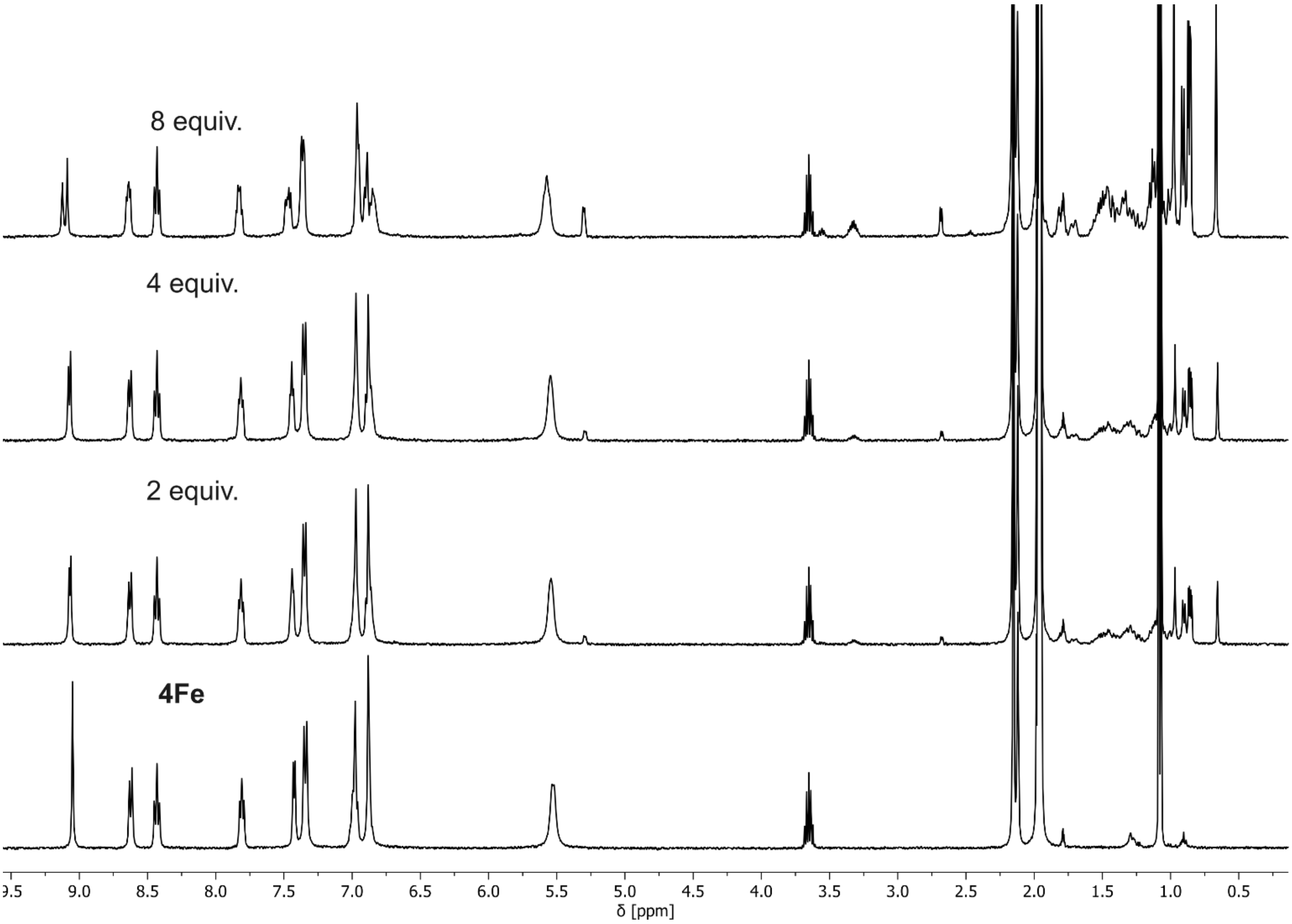

Figure S95. Comparison of ^{1}H NMR spectra of **4Fe** containing different amounts of cholesterol (ca. 8 equiv.). CD_3CN, 298 K, 400 MHz.

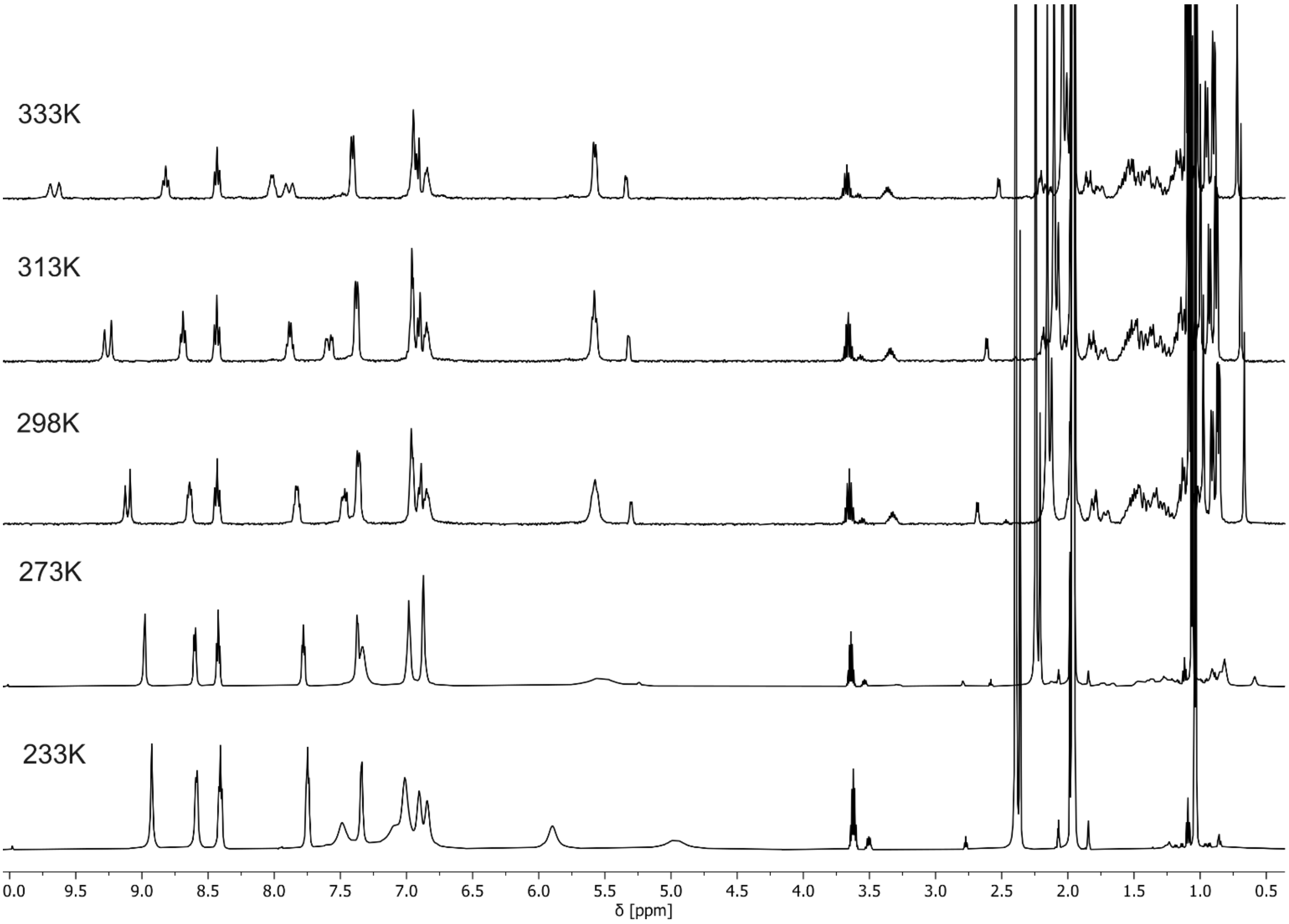

Figure S96. Variable-temperature ^{1}H NMR spectra of **4Fe** containing testosterone (ca. 8 equiv.). CD_3CN, 600 MHz.

4.2.18. Cage 4Zn with cholesterol

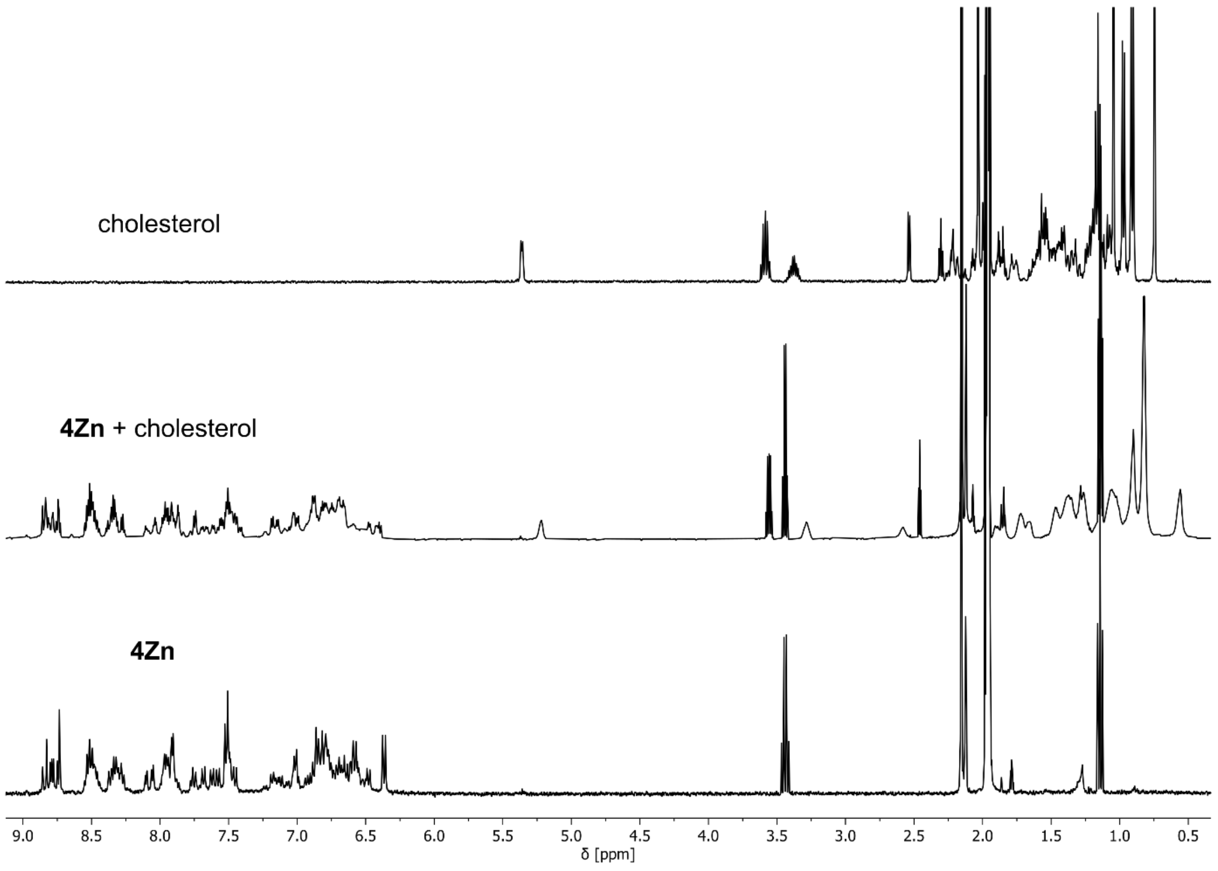

Figure S97. Comparison of ^{1}H NMR spectra of **4Zn**, cholesterol and a mixture of **4Zn** with cholesterol (ca. 5 equiv.). CD_3CN, 298 K, 400 MHz.

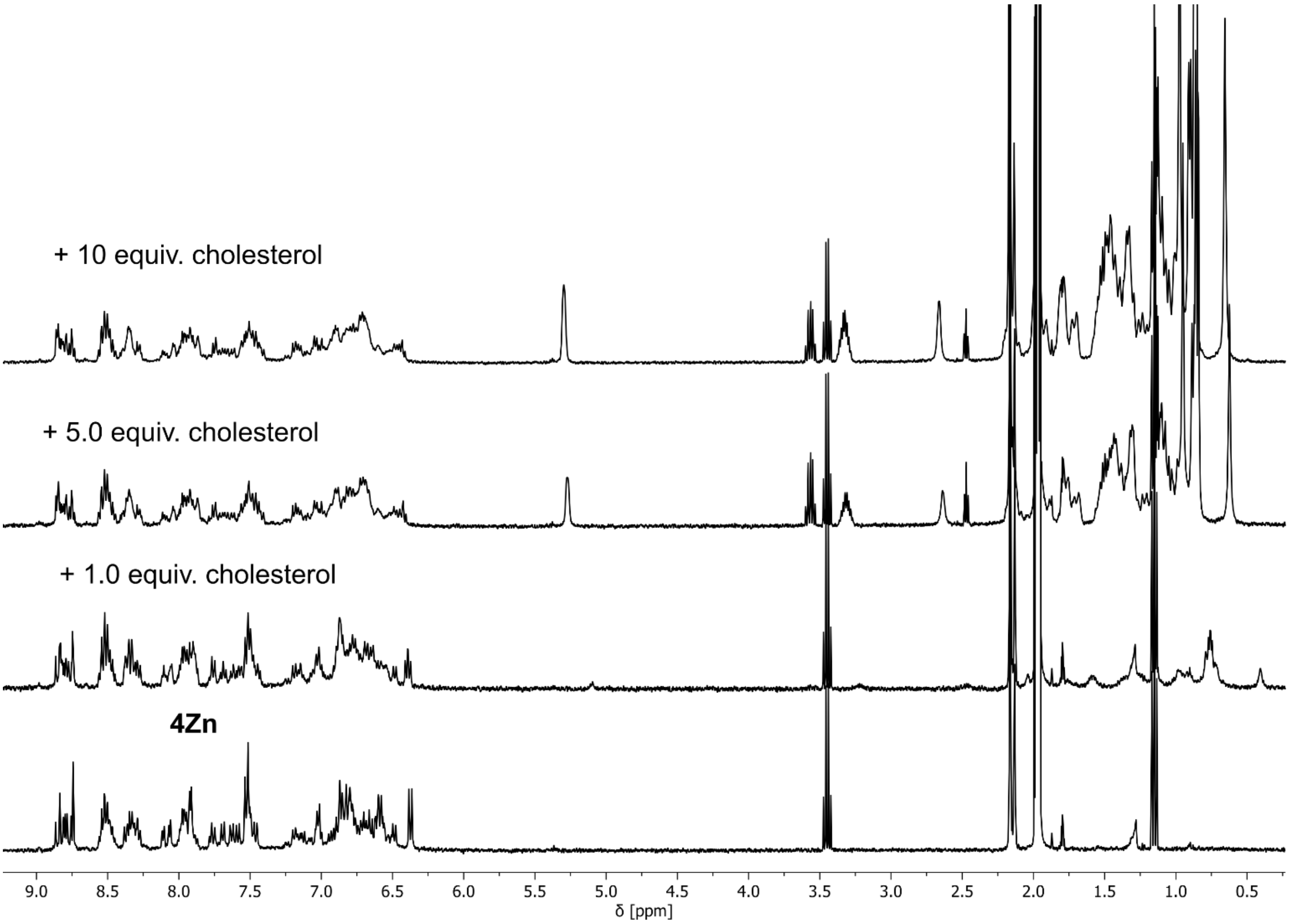

Figure S98. Comparison of ^{1}H NMR spectra of **4Zn** containing different amounts of cholesterol. CD_3CN, 298 K, 400 MHz.

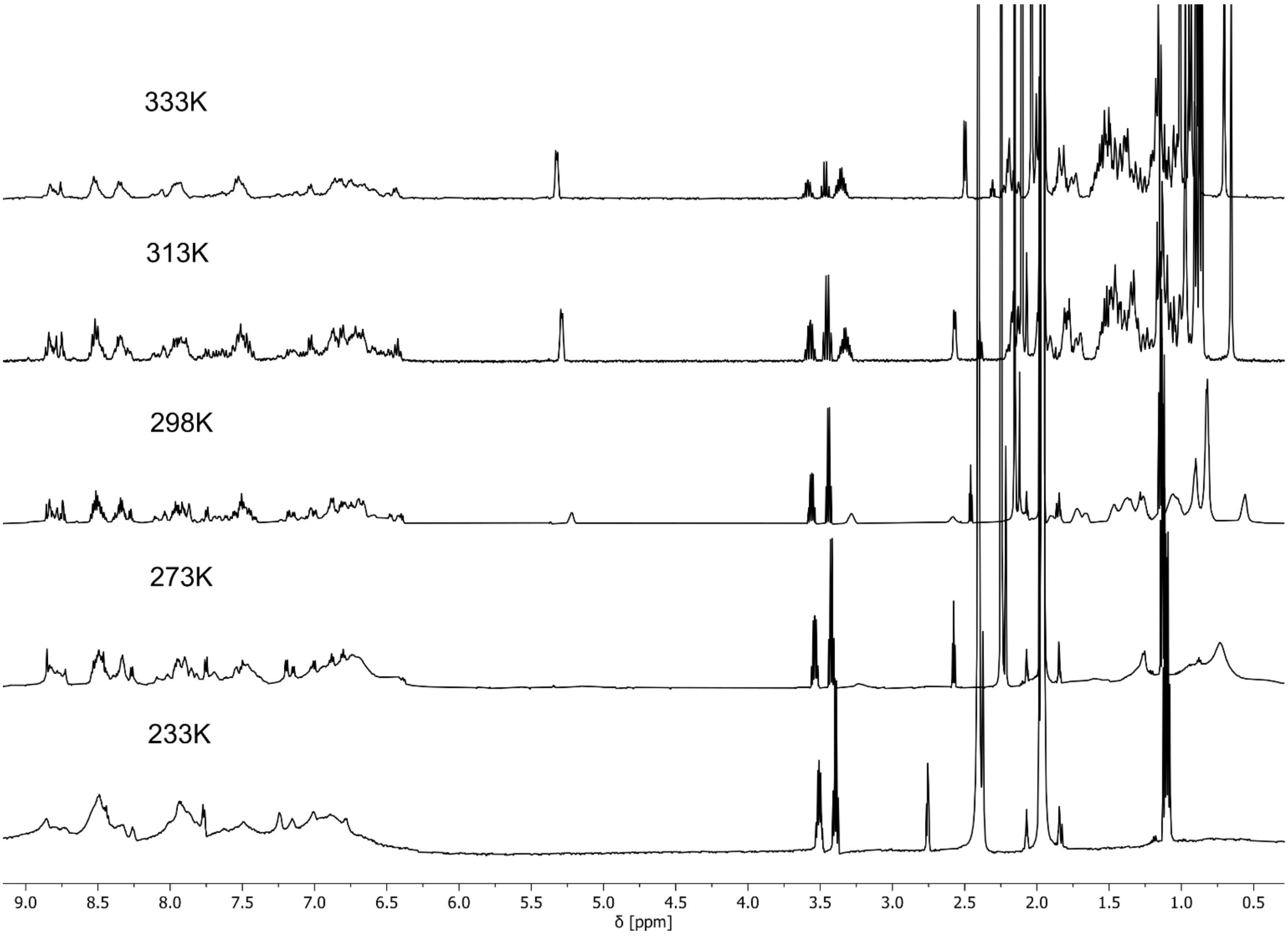

Figure S99. Variable-temperature ^{1}H NMR spectra of **4Zn** containing cholesterol (ca. 5 equiv.). CD_3CN, 600 MHz.

4.2.19. Cage 4Fe with 5,10-dimethyldibenzo[a,e]pentalene

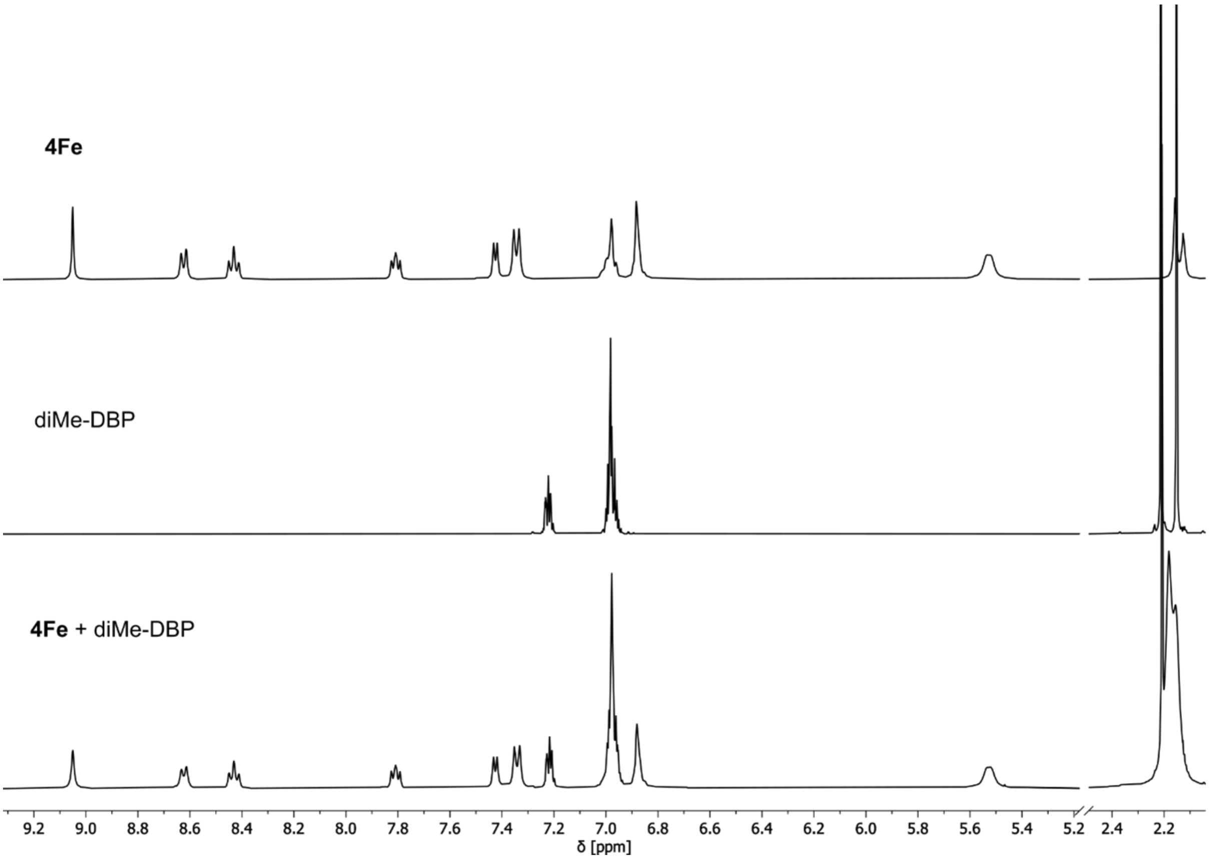

Figure S100. Comparison of ^{1}H NMR spectra of **4Fe**, 5,10-dimethyldibenzo[a,e]pentalene and a mixture of **4Fe** with 5,10-dimethyldibenzo[a,e]pentalene (10 equiv.). CD_3CN, 298 K, 400 MHz.

4.2.20. Cage 4Zn with 5,10-dimethyldibenzo[a,e]pentalene

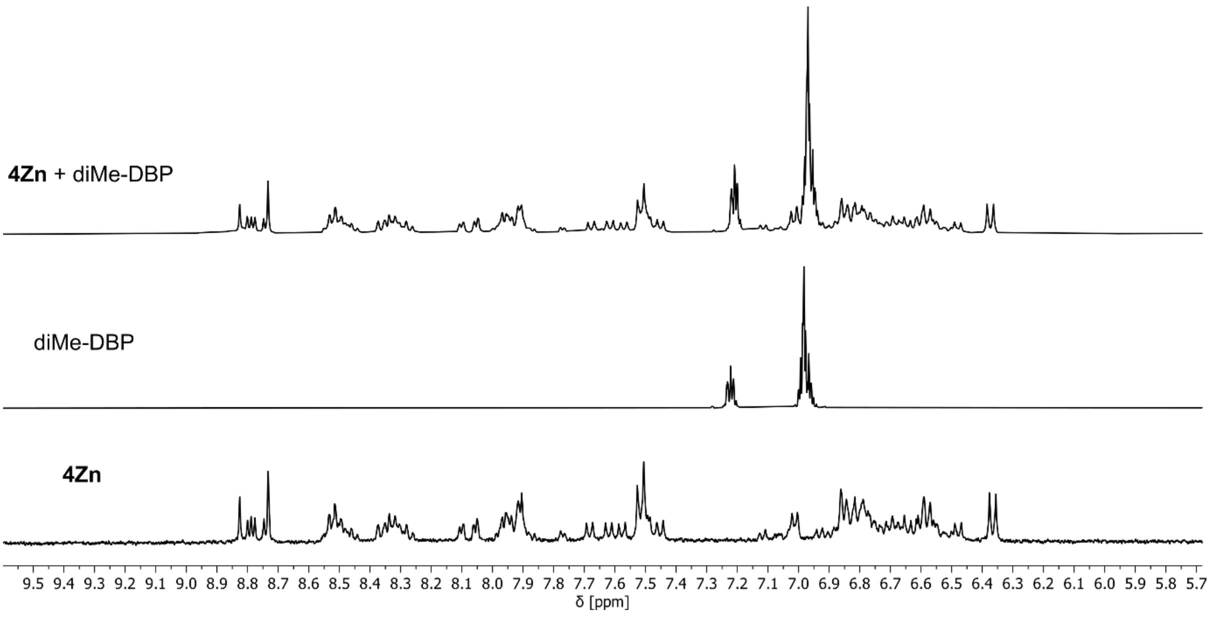

Figure S101. Comparison of ^{1}H NMR spectra of **4Zn**, 5,10-dimethyldibenzo[a,e]pentalene and a mixture of **4Zn** with 5,10-dimethyldibenzo[a,e]pentalene (10 equiv.). CD_3CN, 298 K, 400 MHz.

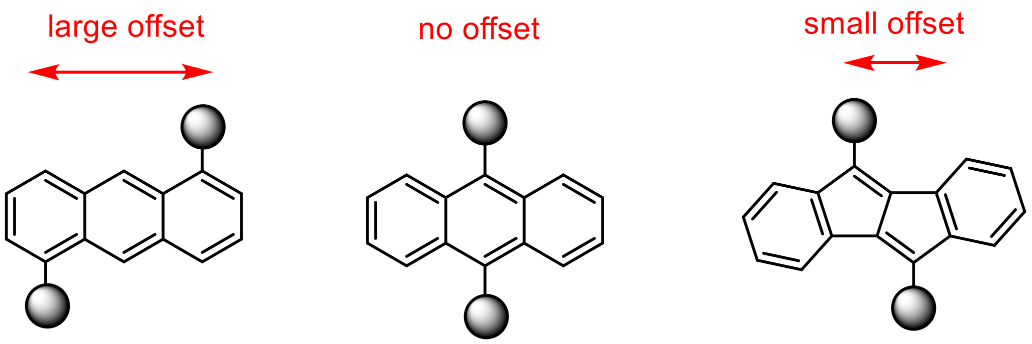

Figure S102. Illustration of the offset between phenyl linkers in the ligands based on anthracene[5] and dibenzo[*a*,*e*]pentalene described in this work.

5. X-ray crystallography

Compound **3** was crystallized by slow evaporation of solvents from a concentrated solution of **3** in a mixture of DMSO and toluene over two weeks.

4Fe and **4Fe·C_{60}** were crystallized by vapor diffusion of benzene into solutions of the compounds in acetonitrile, containing excess of caesium carborane. **4Zn·C_{60}** and **4Fe·$(AQ)_2$** were crystallized by vapor diffusion of diisopropyl ether into solutions of the compounds in acetonitrile, containing excess of caesium carborane, at 5 ^{o}C. All cage crystals employ immediate solvent loss upon removal from mother liquor and need to be manipulated extremely quickly. Crystals were removed from vials using a needle with Paratone oil at the tip and transferred on a glass slide with Paratone oil. For samples taken to synchrotron (**4Fe·C_{60}**, **4Zn·C_{60}** and **4Fe·$(AQ)_2$**), the crystals mounted on MiTeGen loops and pins were flash-frozen in liquid nitrogen and shipped to Diamond Light Source in a cryogenic dewar for remote data collection with a robotic arm.

Data collections for **3** and **4Fe** were performed using Rigaku Synergy R diffractometer equipped with HyPixArc 100° detector at T = 100 K and then processing was done using CrysAlisPro. Data collections for **4Fe·C_{60}**, **4Zn·C_{60}** and **4Fe·$(AQ)_2$** were performed at I19 beamline (instrument I19-1) of Diamond Light Source, employing silicon double crystal monochromated synchrotron radiation (0.6889 Å, Dectris PILATUS 2M detector) with ω and ψ scans at 100 K.[6] Data integration and reduction were undertaken using CrysalisPro.

The structures were solved by SHELXT (version 2018/2)[7] and refined by full-matrix least-squares procedures using SHELXTL (version 2019/2)[8] software package through the OLEX2 graphical interface.[9] All non-hydrogen atoms, including those in disordered parts, were refined anisotropically. Hydrogen atoms were included in idealized positions for structure factor calculations with U_{iso}(H) = 1.2 U_{eq}(C).

Refinement details are given below.

Crystal of **3**

The whole molecule is disordered, and it was modelled by splitting into two parts and treating with restraints. Hydrogen atoms of the -NH_2 groups were found in the difference Fourier map and restrained with DFIX for part 1 (main component). The second component occupancy is very small, and electron density was not possible to be located, so the H atoms positions of the NH_2 groups of that minor disorder component were not modelled.

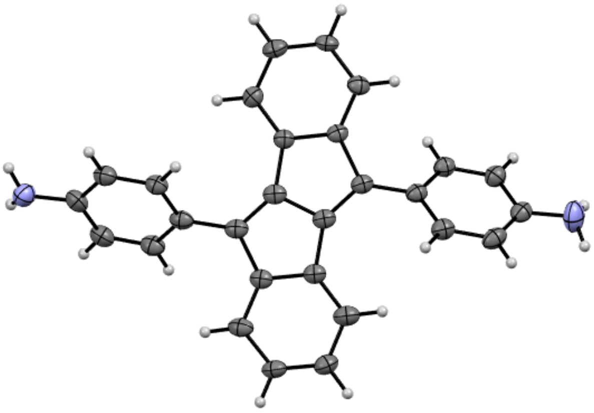

Fig. S103. Displacement ellipsoid plot for **3**. Displacement ellipsoids are set to 50% probability level. Minor disorder component was omitted for clarity.

Crystal of **4Fe**:

4Fe·3(C_6H_6)·7($CB_{11}H_{12}$) + 1 carborane + solvent

The crystals had poor quality and gave weak diffraction, and the data were trimmed during processing accordingly (1.05 Å). Some carborane anions were fitted with rigid groups as all-boron clusters. One of the carboranes could not be modelled satisfactorily and therefore was masked, together with disordered solvent molecules that could not be modelled due to the presence of diffuse electron density in the solvent-accessible voids, and solvent masking (SQUEEZE) was employed. However, the data were enough to establish connectivity, the structure is useful and fit for purpose. Structural parameters such as bond lengths and angles should not be discussed.

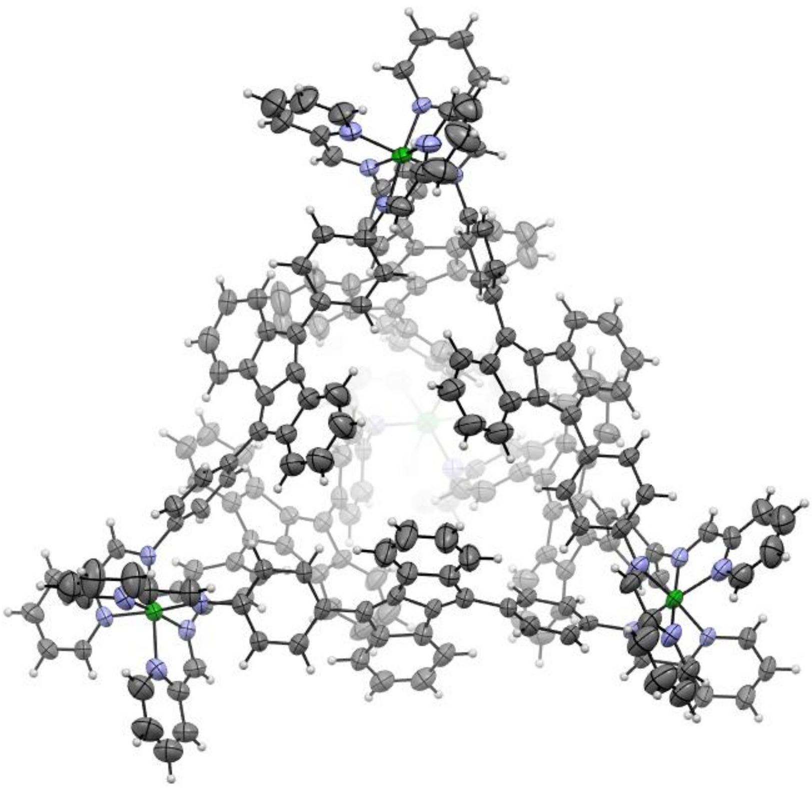

Fig. S104. Displacement ellipsoid plot for **4Fe**. Displacement ellipsoids are set to 50% probability level. Minor disorder components, counterions and solvent molecules were omitted for clarity.

4Fe·2(MeCN)·3(C_6H_6)·C_{60}·5($CB_{11}H_{12}$) + 3 anions + solvent

The crystals had poor quality and gave weak diffraction, and the data were trimmed during processing accordingly (1.05 Å). Some carborane anions were fitted with rigid groups as all-boron clusters. The fullerene is disordered and was modelled as disordered over three positions with SUMP and rigid bodies. The cage walls are disordered as well, and were modelled with support of SIMU, RIGU, SAME, DFIX restraints. Three counterions could not be reliably modelled and were masked (SQUEEZE). However, the data were enough to establish connectivity, the structure is useful and fit for purpose. Structural parameters such as bond lengths and angles should not be discussed.

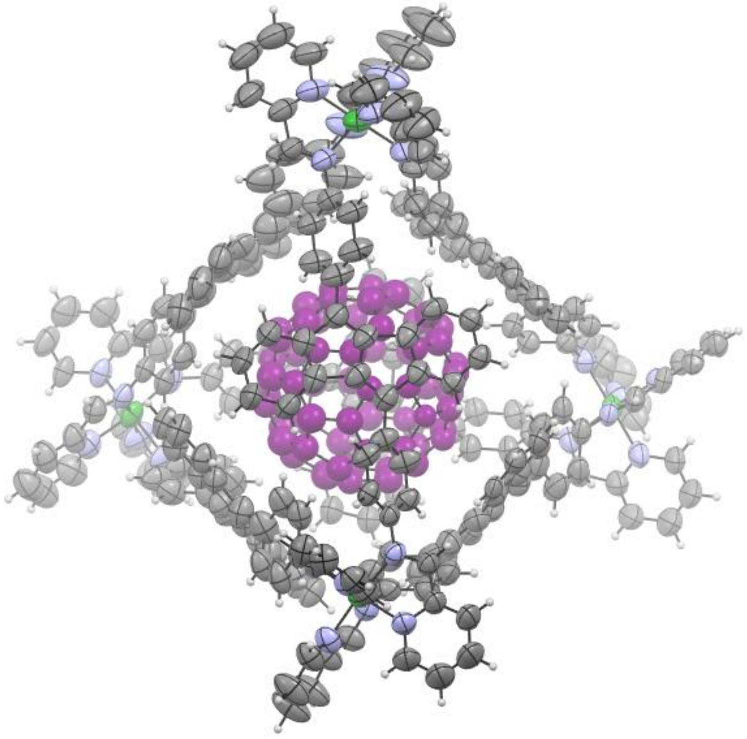

Fig. S105. Displacement ellipsoid plot for **4Fe·C_{60}**. Displacement ellipsoids are set to 50% probability level. Minor disorder components, counterions and solvent molecules were omitted for clarity.

Crystal of **4Zn·C_{60}**:

4Zn·C_{60}·4($CB_{11}H_{12}$) + 4 anions + solvent

The crystals had poor quality and gave weak diffraction, and the data were trimmed during processing accordingly (1.15 Å). The carborane anions were fitted with rigid groups as all-boron clusters. The fullerene is disordered over symmetry element and was modelled using rigid bodies. The cage walls were restrained (SAME). Four counterions could not be reliably modelled and were masked (SQUEEZE). However, the data were enough to establish connectivity, the structure is useful and fit for purpose. Structural parameters such as bond lengths and angles should not be discussed.

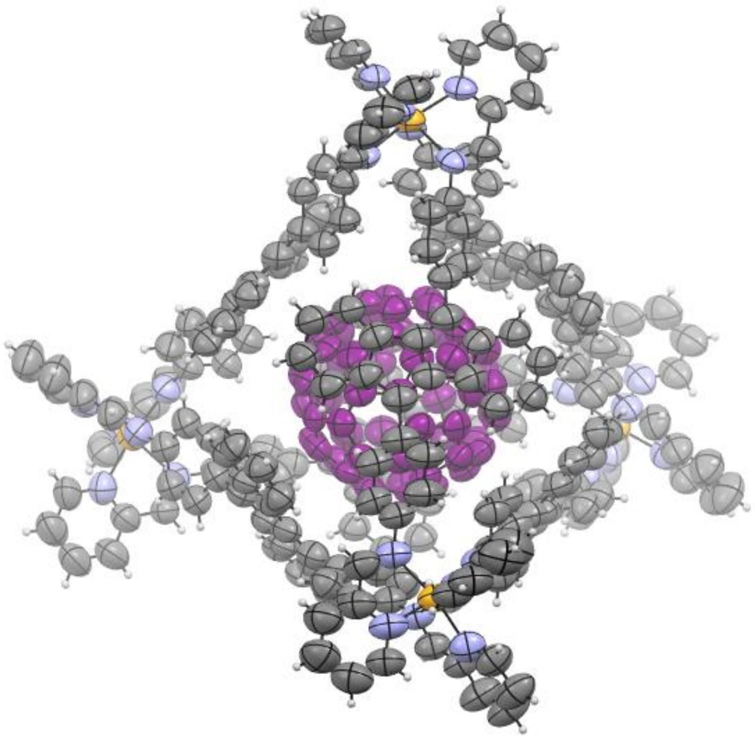

Fig. S106. Displacement ellipsoid plot for **4Zn·C_{60}**. Displacement ellipsoids are set to 50% probability level. Hydrogen atoms, counterions and interstitial solvent molecules were omitted for clarity.

Crystal of **4Fe·(AQ)$_2$**:

4Fe·2.5(AQ) ·3($CB_{11}H_{12}$) + 5 anions + solvent

The crystals had poor quality and gave weak diffraction, and the data were trimmed during processing accordingly (1.00 Å). The carborane anions were modelled as all-boron clusters. The cage walls are disordered as well, and were modelled with support of SIMU, RIGU, SAME, DFIX restraints. Five counterions could not be reliably modelled and were masked (SQUEEZE). However, the data were enough to establish connectivity, the structure is useful and fit for purpose. Structural parameters such as bond lengths and angles should not be discussed.

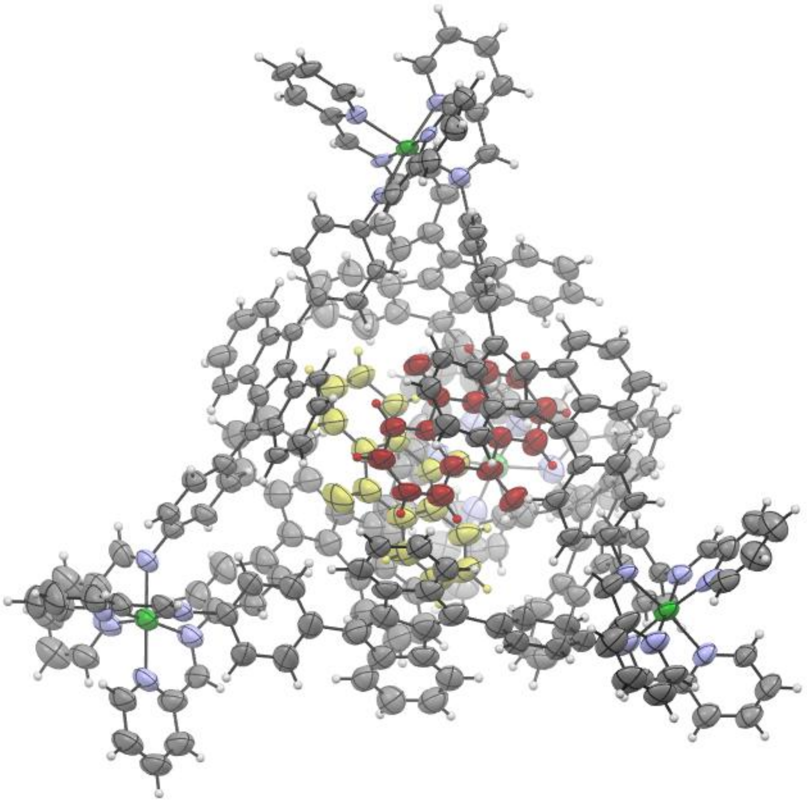

Fig. S107. Displacement ellipsoid plot for **4Fe·(AQ)$_2$**. Displacement ellipsoids are set to 50% probability level. Minor disorder components, counterions and solvent molecules were omitted for clarity.

Table 1. Data collection and refinement details for **3**, **4Fe** and **4Fe·C$_{60}$**.

Compound	**3**	**4Fe**	**4Fe·C$_{60}$**
CCDC number	2542325	2542326	2542327
Empirical formula	$C_{28}H_{19}N_2$	$C_{258}H_{258}B_{84}Fe_4N_{24}$	$C_{322}H_{240}B_{60}Fe_4N_{26}$
Formula weight	383.36	4826.31	5345.53
Temperature/K	100.00(10)	100.00(11)	100.15
Crystal system	orthorhombic	monoclinic	monoclinic
Space group	*P*bca	*C*2/c	*P*2$_1$/n
a/Å	10.50518(5)	49.9694(7)	29.4399(4)
b/Å	17.65551(9)	28.8582(3)	29.7142(4)
c/Å	20.73956(11)	55.2247(11)	40.8029(4)
α/°	90	90	90
β/°	90	107.495(2)	95.5720(10)
γ/°	90	90	90
Volume/Å^3	3846.65(3)	75952(2)	35525.0(8)
Z	8	8	4
ρ_{calc}g/cm^3	1.324	0.844	0.999
μ/mm^{-1}	0.598	1.506	0.195
F(000)	1607	19984	11032
Crystal size/mm^3	0.20 × 0.10 × 0.10	0.30 × 0.20 × 0.10	0.20 × 0.10 × 0.02
Radiation	Cu Kα (λ = 1.54184)	Cu Kα (λ = 1.54184)	synchrotron (λ = 0.6889)
2Θ range for data collection/°	8.526 to 145.874	3.708 to 94.48	2.98 to 38.3
Reflections collected	80181	275032	255338
Independent reflections	3819 [R_{int} = 0.0297, R_{sigma} = 0.0112]	34376 [R_{int} = 0.0931, R_{sigma} = 0.0398]	32102 [R_{int} = 0.0795, R_{sigma} = 0.0364]
Data/restraints/parameters	3819/1872/409	34376/6630/3103	32102/13964/3625
Goodness-of-fit on F^2	1.296	1.032	1.065
Final R indexes [I>=2σ (I)]	R_1 = 0.0652, wR_2 = 0.1424	R_1 = 0.0978, wR_2 = 0.2642	R_1 = 0.0975, wR_2 = 0.2791
Final R indexes [all data]	R_1 = 0.0655, wR_2 = 0.1425	R_1 = 0.1069, wR_2 = 0.2704	R_1 = 0.1100, wR_2 = 0.2908
Largest diff. peak/hole / e Å^{-3}	0.21/-0.17	0.59/-0.34	0.85/-0.34

Table 2. Data collection and refinement details for **4Zn·C_{60}** and **4Fe·(AQ)$_2$**.

CCDC number	2542328	2542329
Compound	**4Zn·C_{60}**	**4Fe·(AQ)$_2$**
Empirical formula	$C_{300}H_{204}B_{48}N_{24}Zn_4$	$C_{279}H_{218}B_{36}Fe_4N_{26}O_5$
Formula weight	4925.22	4627.34
Temperature/K	100	100.15
Crystal system	trigonal	monoclinic
Space group	R-3c	$P2_1$/c
a/Å	28.7662(3)	28.3703(5)
b/Å	28.7662(3)	34.6921(3)
c/Å	140.2899(12)	41.6717(7)
α/°	90	90
β/°	90	108.5496(18)
γ/°	120	90
Volume/Å^3	100536(2)	38883.6(11)
Z	12	4
ρ_{calc}g/cm^3	0.976	0.79
μ/mm^{-1}	0.308	0.173
F(000)	30384	9592
Crystal size/mm^3	0.20 × 0.15 × 0.04	0.20 × 0.10 × 0.02
Radiation	synchrotron (λ = 0.6889)	synchrotron (λ = 0.6889)
2Θ range for data collection/°	2.872 to 34.854	2.936 to 40.296
Reflections collected	183965	309510
Independent reflections	7689 [R_{int} = 0.2300, R_{sigma} = 0.0485]	40703 [R_{int} = 0.1434, R_{sigma} = 0.0586]
Data/restraints/parameters	7689/4352/1357	40703/7275/3153
Goodness-of-fit on F^2	1.079	1.054
Final R indexes [I>=2σ (I)]	R_1 = 0.1266, wR_2 = 0.3624	R_1 = 0.1540, wR_2 = 0.4291
Final R indexes [all data]	R_1 = 0.1339, wR_2 = 0.3757	R_1 = 0.1651, wR_2 = 0.4421
Largest diff. peak/hole / e Å^{-3}	0.94/-0.44	0.97/-0.74

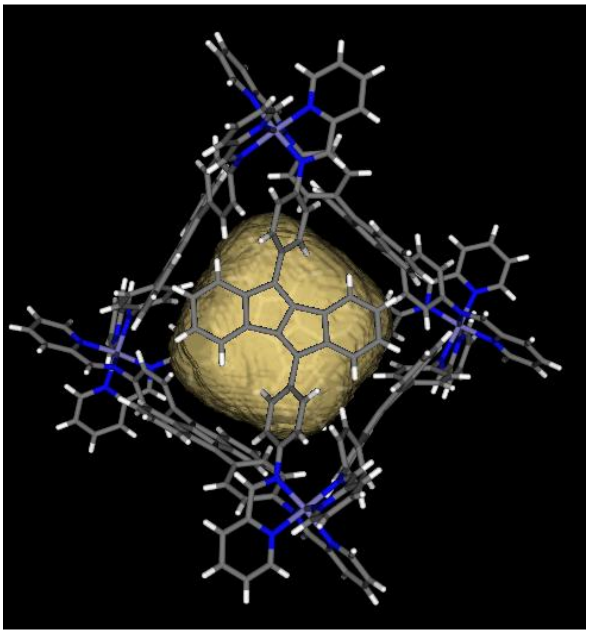

Fig. S108. Void visualized with MoloVol software[10] for the molecular structure of **4Fe** crystallized from MeCN solution containing excess of caesium carborane, and benzene; void volume: 566 Å^3, probe radius: 2.5 Å.

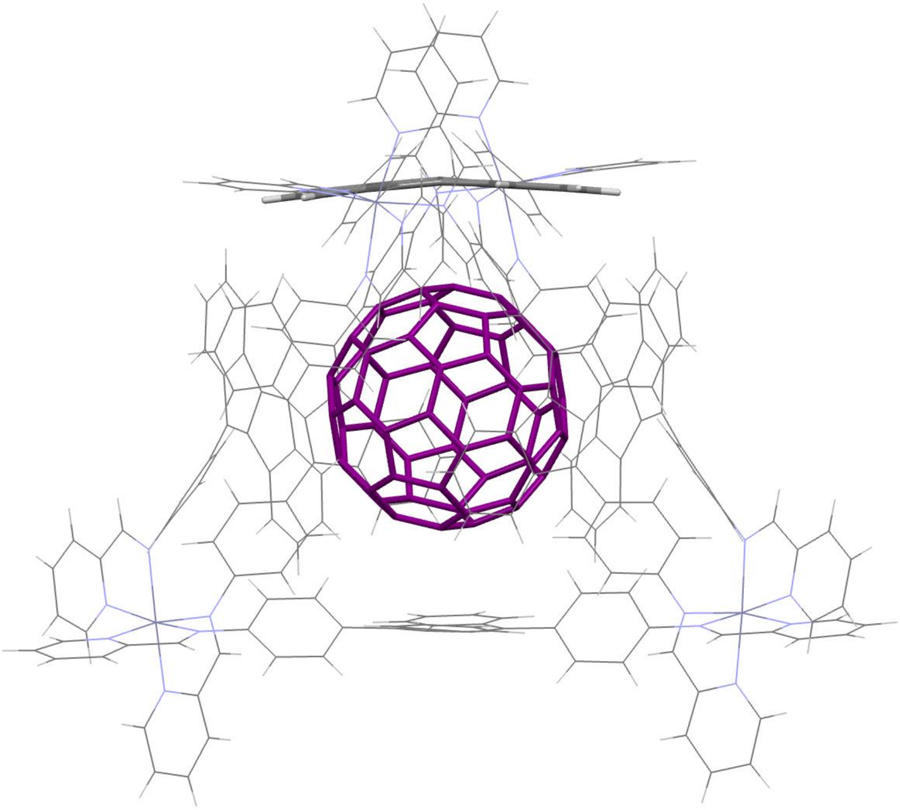

Fig. S109. View on the structure of **4Fe·C$_{60}$** showing a slight kink in the structure of DBP.

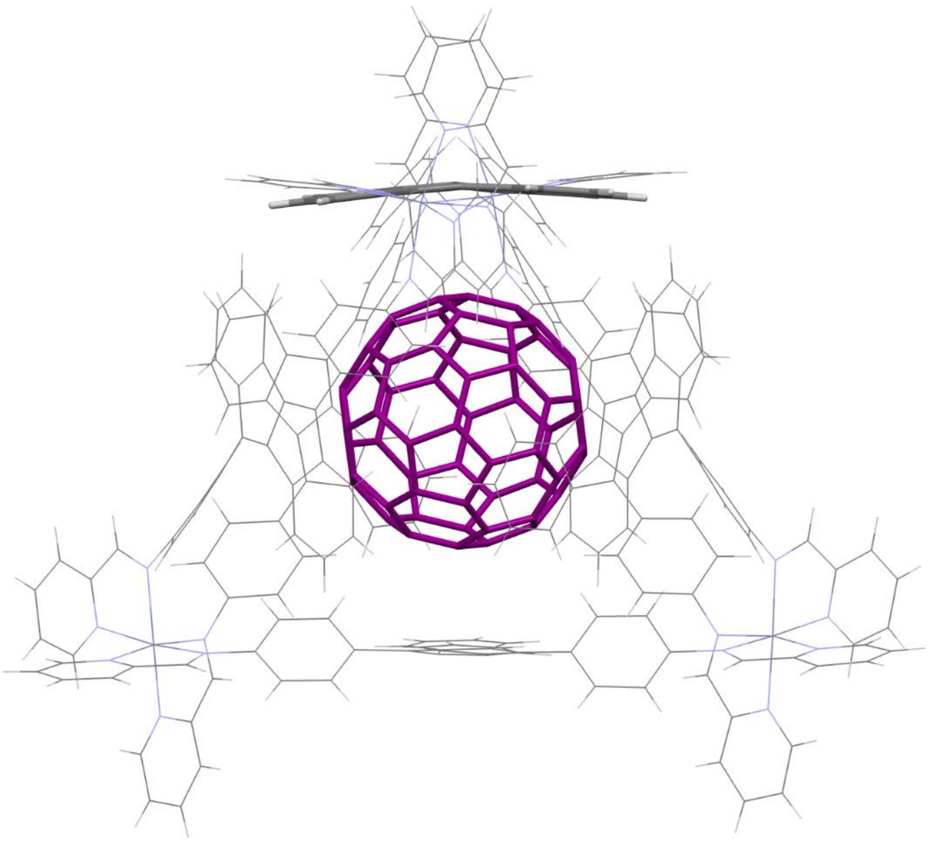

Fig. S110. View on the structure of **4Zn·C₆₀** showing a slight kink in the structure of DBP.

6. UV-Vis spectroscopy

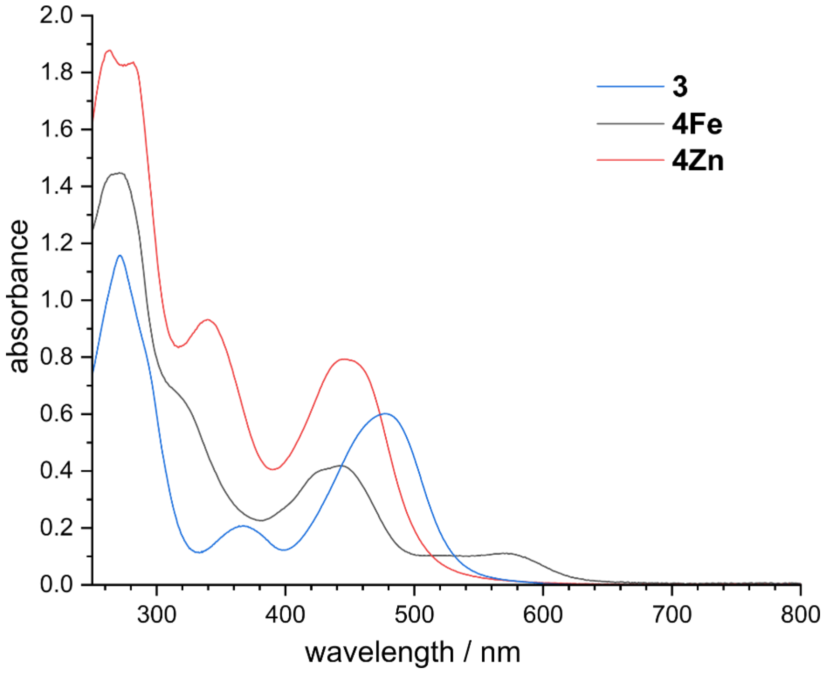

Figure S111. Comparison of UV-Vis absorption spectra of **3**, **4Fe** and **4Zn** in MeCN. Note that the concentrations are different.

8. Theoretical calculations

Using the coordinates of the X-ray structure of empty cage **4Fe** as a starting point, its geometry was optimized using Grimme's GFN2-xTB,[11] and NICS calculations were performed on the obtained geometry using the same level of theory as Nitschke's norcorrole cage: B3LYP/SDD (for Fe), 6-31G(d) (for C, H, N), using Gaussian16.[12,13] Calculations on the same level of theory were performed for parent, unsubstituted dibenzo[*a*,*e*]pentalene, and isotropic components of the magnetic shielding were extracted from the output file. The dummy atoms (Bq) were generated from the middle of the fused pentalene motif towards the centroid of the cage (defined as a centroid of the four Fe atoms), with equal spacing (Figure S112), or from the centroid of one of the pentagons in the DBP units, in a direction perpendicular to the DBP plane towards inside of the cage. A similar set of dummy atoms was generated for the parent, unsubstituted dibenzo[*a*,*e*]pentalene (Figure S113). Similar calculations were performed for the Bq atoms starting at the centroid of phenyl rings in the DBP. The results show that the deshielding in the cage volume is very small, consistent with a low antiaromaticity of pentalene in the DBP, and it rapidly diminished with increasing distance from the molecular plane. Beyond ca. 1.5 Å it is shielding of the Clar sextets in the phenyl rings that dominates (Figure 5 in the main text). AICD calculations were performed as well.[14]

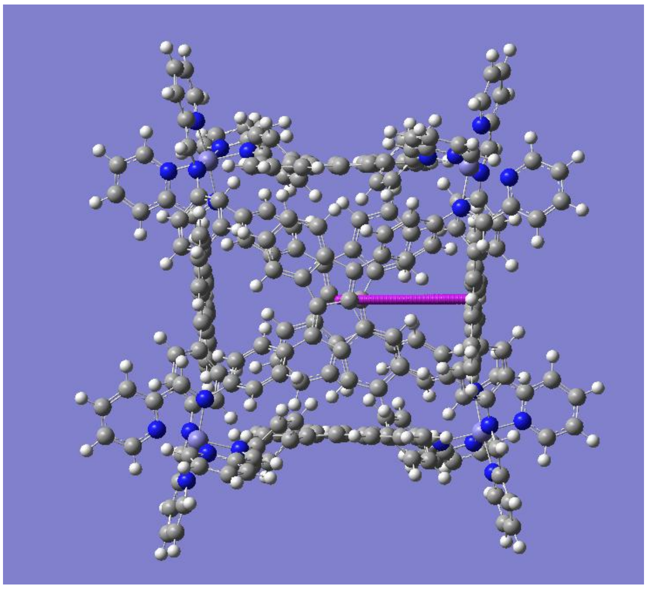

Figure S112. Structure of the cage and generated dummy atoms (violet).

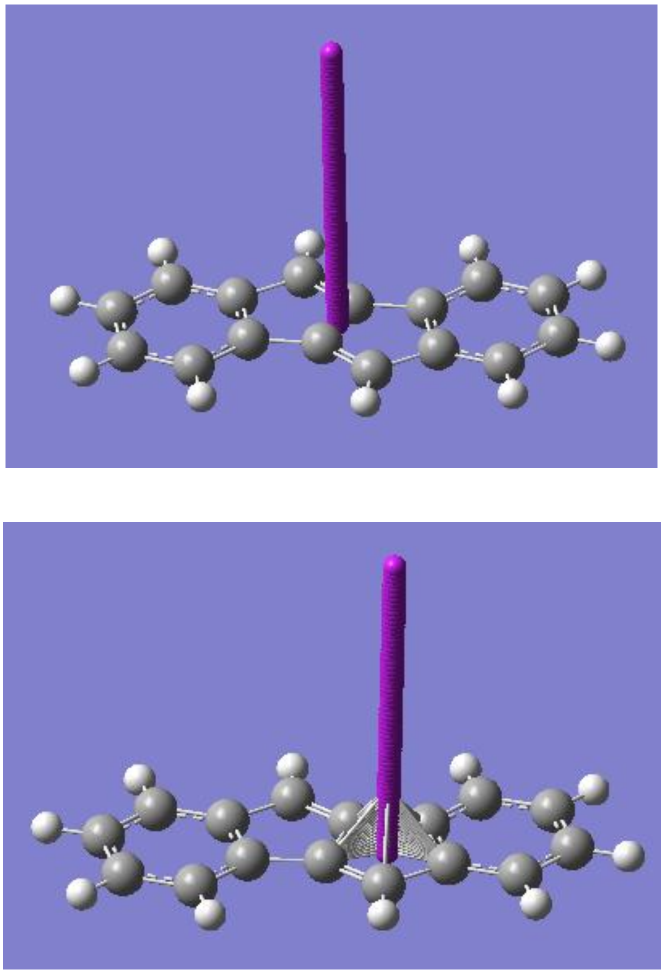

Figure S113. Structure of the parent, unsubstituted DBP and generated dummy atoms (violet).

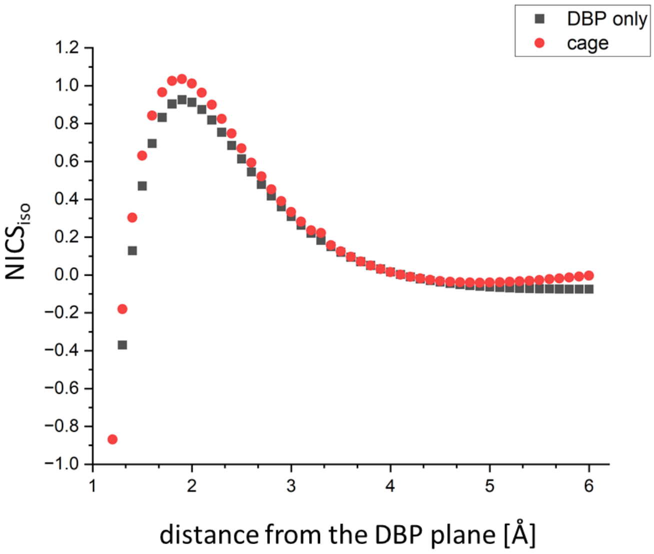

Figure S114. Comparison of the obtained NICS$_{(iso)}$ values for the cage and for the parent DBP calculated for the points above the middle-bond between pentagons.

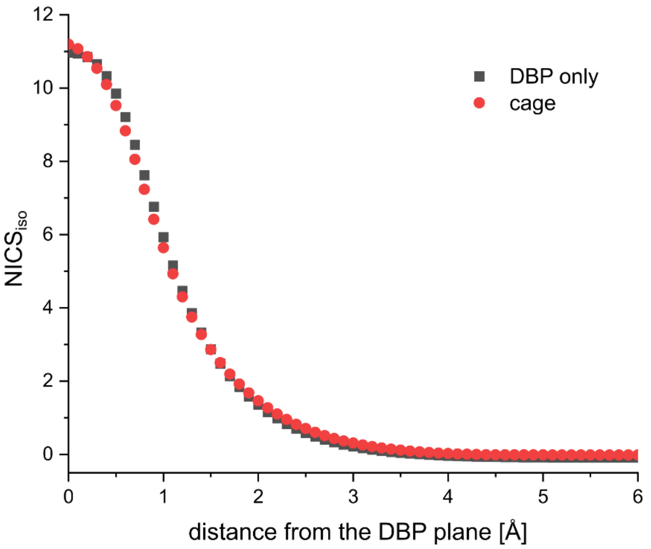

Figure S115. Comparison of the obtained NICS$_{(iso)}$ values for the cage and for the parent DBP calculated for the points above the centroid of one of the pentagons.

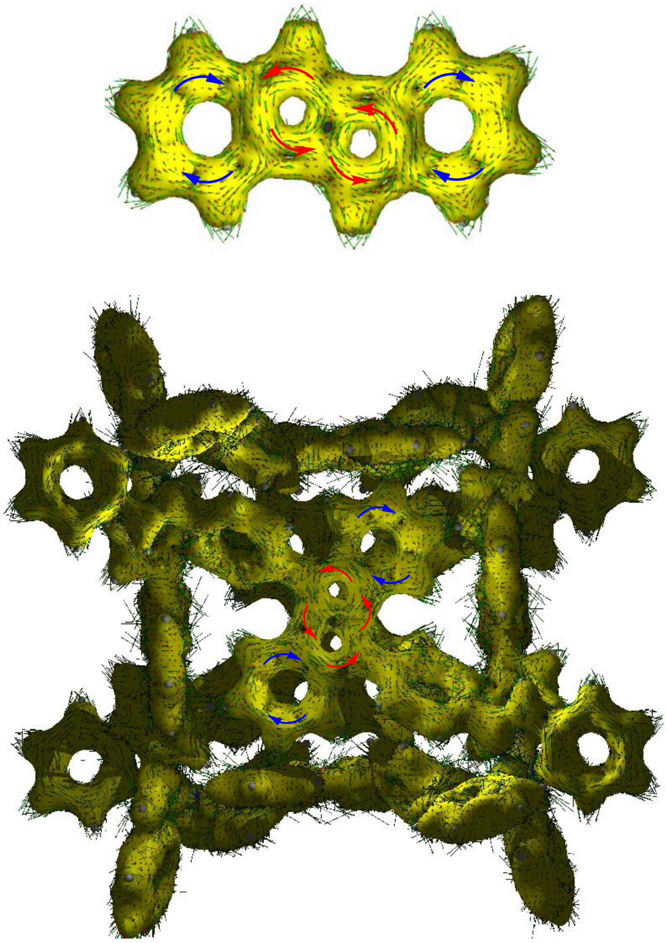

Figure S116. Calculated AICD plots for parent DBP (top) and cage **4Fe** (bottom). Isovalue: 0.04.

Computational data are available free of charge in Zenodo public repository: https://zenodo.org/records/19519946

Atomic coordinates of **4Fe** from xTB calculations:

Fe	-11.03143712	-0.14378437	44.71243257
Fe	5.69172668	4.91586732	44.69342373
Fe	-7.03908127	16.88291394	44.69676508
Fe	-4.12745460	7.20484921	30.42903585
N	4.48209851	3.54890038	44.07530283
N	-7.91344192	16.03306680	43.15785177
N	-12.61245512	-0.73737911	43.67179661
N	5.38964907	6.06705647	43.17878354
N	4.37262776	5.78374042	45.79851072
N	6.99650673	3.89340590	43.67036248
N	-5.21582888	16.52372828	44.06144348
N	-6.76387504	18.54920072	43.65457715
N	-9.85998612	-0.47865403	43.17319222
N	-11.62955219	1.61454320	44.07574250
N	-5.74563702	7.01834649	31.45937616
N	-3.47886885	8.70182420	31.45538580
N	-3.15374202	5.89612862	31.45597450
N	-6.32799840	17.67985853	46.36941904
N	-8.91642255	17.34089134	45.14436468
N	-10.49092759	-1.99822546	45.16147026
N	-9.60074816	0.57606939	45.84989583
N	6.03617414	3.89345175	46.31567124
N	-7.13032733	15.28460488	45.83521252
N	7.00203514	6.27597413	45.16894839
N	-12.07674669	0.07625065	46.38484739
C	-4.01500255	9.26997642	32.62656394
N	-5.22704247	8.40181205	29.35484225
C	-7.90647541	1.79798227	41.01382908
H	-6.91198133	2.21555225	40.96680306
C	-10.46372568	0.73650799	41.15554037
H	-11.44380389	0.28666165	41.18464678
C	3.09819464	3.42999071	44.30279363
C	-6.13836726	13.83440851	40.03618214
C	-9.35979894	4.22079847	38.58965067
N	-2.54297163	7.55694931	29.35184784
C	6.45279719	2.80686306	43.08386081
N	-4.61729805	5.65457983	29.35524699
C	-6.55716037	15.94474149	41.14168222
H	-6.45569740	17.01838472	41.16971529
C	-4.42920639	15.37884804	44.28574274
C	-9.52350344	0.40333768	42.12932591
C	-6.91890835	13.19950041	41.00292390
H	-7.05547574	12.12951788	40.95712583
C	-5.06027126	11.75788206	37.11866976
C	-8.84763468	2.15462110	40.04738878
C	-7.31721088	15.29893811	42.11560843
C	-0.99559169	11.42422385	44.28992667
C	3.74159301	4.80324544	40.12309113
H	3.91436625	4.06450525	39.35552180
C	-7.45792847	3.05427508	38.11328642
C	4.65381520	4.94429314	41.15055053
H	5.53555650	4.32370923	41.17930545
C	-11.02946720	2.86752796	44.29931776

C	1.09169837	4.15596845	38.57459166
C	-9.71087547	9.81499405	43.24412059
C	-7.03461056	11.67603894	45.04078242
H	-6.30588860	10.88390897	44.95645488
C	1.65853267	5.45782429	38.95846389
C	-5.19210918	10.01453719	32.57746752
H	-5.67623616	10.18621109	31.62899912
C	2.61959808	5.63249869	40.03762849
C	-3.88723722	15.12987466	45.54573863
H	-4.04138624	15.84080763	46.34231961
C	2.45244492	6.62292531	41.00600215
H	1.59311124	7.27456907	40.95709937
C	-2.86461529	13.09581454	44.72907543
C	-4.09278554	13.24130289	38.58011757
C	-7.50630227	13.92104430	42.02262274
H	-8.10365074	13.42027798	42.76698109
C	-2.39232076	6.07703775	32.62599934
C	5.04304936	2.67412534	43.32141982
H	4.49372109	1.84397831	42.88610776
C	-5.97592363	15.22037356	40.11944373
H	-5.41579868	15.73507138	39.35378714
C	-6.05469476	12.17881156	38.10389108
C	-3.39036349	13.36610663	43.46515140
H	-3.20579373	12.67750159	42.65438277
C	-5.96716354	6.27042717	32.63117462
C	-8.57007059	8.87479729	44.98178172
C	6.12072962	7.12247739	43.18644684
H	6.06371004	7.87454084	42.40445330
C	-5.55011768	18.60541635	43.06292477
C	-1.08867456	3.10724125	45.00596678
C	-5.50512942	13.08541324	38.96064159
C	-9.64165791	-2.53301836	44.25633367
C	0.34117605	3.20261703	44.75150319
C	-8.51729667	3.07677243	38.97071255
C	-0.11141456	11.72793925	43.18269788
C	-2.22340747	1.50923776	43.20310866
C	-8.23619449	0.92954356	42.03490445
H	-7.50318995	0.66430312	42.77924074
C	0.83375507	3.53228117	43.48845101
H	0.14256250	3.71921974	42.68051035
C	1.14737801	6.38729945	38.10262124
C	4.46393081	5.91890160	42.12855544
C	3.46156798	6.79884537	45.45124511
C	-0.88029871	6.42612524	34.96003873
C	-13.26716430	0.28562517	43.07911622
C	-13.10672413	-1.96124318	43.52205101
H	-12.57210539	-2.76198439	44.00929401
C	-2.84162240	5.57107208	33.84428203
H	-3.78472745	5.05127720	33.88249698
C	-5.61737356	1.43402375	44.79032397
C	-2.04188827	2.42071279	44.31488672
C	0.71419869	9.86824143	44.37338455
C	-6.23911485	4.15135340	33.73724414
H	-6.30741157	3.07559107	33.68357248
C	-5.07258534	10.40277124	34.96199848
C	-9.79994789	4.76981087	43.47884498
H	-9.29268762	5.27152545	42.66857296
C	0.93624220	10.77199036	43.23433854
C	-0.45190700	10.24875855	44.96759377

C	0.28864175	5.73628258	37.11500111
C	-7.54799726	13.98859095	45.48026723
C	2.19191100	3.63611174	43.26453400
H	2.56150681	3.89424748	42.28592128
C	-7.31139490	11.55770305	37.73636311
C	-9.19594040	16.12707871	43.16889688
H	-9.81627185	15.68763510	42.39262418
C	2.09783184	6.52501477	45.36681240
H	1.74859755	5.52440954	45.56104691
C	-3.09005942	13.98991862	39.16055891
H	-3.27869478	14.59742635	40.03394996
C	3.36473695	6.76985473	42.03154689
H	3.22448877	7.53621020	42.77582490
C	-7.82587451	2.18056344	45.41409251
H	-8.51843308	2.98176423	45.61437145
C	-0.09698630	6.63796202	36.16850098
C	-0.41993395	6.90219538	33.72910249
H	0.54474999	7.38299678	33.67413931
C	-8.37739299	11.40548429	44.77511647
C	-3.81916921	12.42903764	37.44894616
C	-6.41568283	4.78962620	34.96819520
C	-9.31365790	7.81607817	44.30151842
C	-5.71279723	10.56867876	33.73057203
H	-6.60947845	11.16682342	33.67591463
C	-4.15399884	14.49446374	43.24400908
H	-4.55957372	14.69161844	42.26506548
C	-1.16134312	6.72867653	32.57667967
H	-0.77310820	7.06569800	31.62846717
C	-8.79452718	4.86329974	37.45745109
C	-10.01430613	8.42962781	43.19130433
C	-7.05998758	10.81805961	36.55150226
C	-12.66318627	1.56955556	43.31209039
H	-13.09545956	2.46196784	42.86795412
C	-10.12856865	1.60125609	40.13212837
H	-10.85511899	1.82750720	39.36674883
C	2.61668700	3.07026967	45.56026396
H	3.31173770	2.84214605	46.35287815
C	1.24171175	2.91265895	39.15291492
H	1.86138900	2.77267606	40.02697723
C	-8.30515085	6.90684927	46.13473544
C	-11.08698727	3.46351166	45.55819707
H	-11.62851005	2.97680368	46.35419916
C	-6.62658202	12.94607287	45.39577070
H	-5.58612299	13.14603101	45.59352554
C	-6.02117749	4.87861670	32.58337121
H	-5.92760959	4.37282265	31.63529345
C	-5.64804398	10.97476984	36.17062801
C	-3.35439663	9.12835842	33.84534751
H	-2.43463210	8.56836114	33.88360383
C	-8.82173650	10.07499230	44.38672984
C	-9.80310987	16.87092685	44.23914610
C	-1.38300612	1.04858088	42.21178449
H	-0.34875930	1.35501703	42.17240803
C	-2.10031419	5.75017818	34.99478791
H	-2.47239641	5.37265853	35.93531096
C	4.31805297	5.31316868	46.99182452
H	3.61968834	5.69024528	47.73379184
C	-3.32966841	2.53273526	44.99723929
C	-7.59238532	4.12467343	37.12695898

C	-9.30135778	-1.63689184	43.18461478
H	-8.61206540	-1.95596156	42.40771380
C	5.24332342	4.27189595	47.33954099
C	-6.62147503	4.00639375	36.17778049
C	-9.83109627	5.36250304	44.74161728
C	1.23474727	7.78715400	37.73816731
C	0.46985086	7.93973289	36.55250136
C	-1.75680432	3.74421814	46.15100438
C	-3.11821487	14.00325044	45.76174758
H	-2.67843680	13.83675504	46.73322170
C	-10.21086766	10.67582817	42.28926034
H	-9.97320055	11.72972233	42.30579930
C	-6.06068303	0.11302699	44.90293950
H	-5.36245270	-0.69511988	44.74728751
C	-11.11647216	5.83281583	38.60559120
H	-12.02626723	6.21774852	39.03959947
C	-6.29155904	2.27682944	37.74548726
C	7.05642406	7.27559839	44.26450607
C	-6.52191159	2.46184802	45.05928271
H	-6.19919170	3.48887563	44.97762443
C	-1.89099602	0.16287613	41.26431316
H	-1.24315242	-0.21485154	40.48891940
C	-8.26945721	0.86155079	45.49490908
C	0.25265488	4.32508434	37.44249972
C	-10.39714196	3.54507225	43.25813820
H	-10.36360056	3.09384704	42.27993225
C	-7.57758373	19.58817949	43.50310677
H	-8.53887458	19.52487441	43.98926151
C	1.64262268	8.81560166	44.75877411
C	-0.21944726	1.98887513	37.46871228
H	-0.71005994	1.12962297	37.03905682
C	-3.57418499	1.07736760	43.25720294
C	-4.06964427	0.21469226	42.30163257
H	-5.10053104	-0.10840370	42.31918124
C	-10.83699470	7.93322246	42.20234882
H	-11.09076379	6.88477278	42.16418369
C	-9.19469675	6.64718255	44.99244126
C	-6.17527027	6.91414324	33.84949230
H	-6.15206467	7.99076863	33.88661287
C	-4.73895057	17.44157416	43.29739067
H	-3.74957785	17.37082847	42.85389466
C	0.32975913	9.18072237	35.96679173
H	-0.26510935	9.31512148	35.07486227
C	1.20346258	7.51687810	45.01763601
H	0.15274247	7.28395904	44.93257777
C	-11.34364293	0.58296384	47.40082139
C	-2.29111258	10.99950478	46.11912850
C	-7.36614291	-0.17201816	45.25182093
H	-7.68236672	-1.19612928	45.37363203
C	-5.77865945	2.86295321	36.55908801
C	-4.24315943	1.71483168	44.40178186
C	-0.39653020	3.24251475	36.88783407
H	-1.02049351	3.35616291	36.01436618
C	-2.06921814	11.90302489	44.97977934
C	1.93398293	10.77749934	42.28182535
H	2.72729105	10.04402947	42.29752633
C	-8.89511551	13.72196204	45.24043733
H	-9.62437288	14.50707202	45.36481143
C	8.29967025	4.09741496	43.51209498

H	8.71723260	4.96735877	43.99379080
C	3.90597507	8.09996207	45.22300225
H	4.95014872	8.33779243	45.35132603
C	-10.50958236	4.71429646	39.17038687
H	-10.94038658	4.24749304	40.04445036
C	-5.96233863	18.93360887	46.61224648
H	-6.03401278	19.62674273	45.78831436
C	-2.38164004	9.19166439	31.00303921
H	-1.88318867	10.03377680	31.47512376
C	-9.40601822	5.96888549	36.90532040
H	-8.99601571	6.45279371	36.03186296
C	-1.55765815	13.18135443	37.48042871
H	-0.56711764	13.18297801	37.05331068
C	1.25736891	2.96192110	45.77937728
H	0.89716091	2.65498332	46.74932374
C	-1.29920687	9.98444960	46.11315484
C	-0.12710288	12.69251242	42.19751669
H	-0.90775372	13.43703350	42.16043729
C	-8.57177105	11.57102848	38.29534886
H	-8.77966929	12.14654860	39.18454617
C	-3.13170909	3.39219429	46.14722126
C	-9.98080434	0.86921556	47.04294532
H	-9.30438186	1.29567186	47.77857577
C	0.57911959	1.82760584	38.58541268
H	0.70271444	0.84648450	39.01715759
C	-3.21197636	-0.24220593	41.30465976
H	-3.58285058	-0.93174846	40.56226464
C	1.87251495	8.87245475	38.30077685
H	2.47356609	8.76446421	39.19084087
C	-3.87979325	9.67996387	34.99654084
H	-3.36844816	9.54240393	35.93736804
C	-1.81772894	13.95445913	38.59638703
H	-1.02904642	14.54942075	39.03022459
C	-14.42656991	0.09735760	42.32603510
H	-14.91707406	0.94439343	41.87342812
C	-5.67125655	1.18010561	38.30551240
H	-6.06416447	0.71345471	39.19600625
C	-6.25628226	16.79241664	47.38615206
C	-1.82963719	8.60411703	29.81512280
C	-10.57812912	6.44423428	37.48887853
H	-11.07559005	7.30065213	37.06144558
C	-10.49416223	4.69210161	45.77366642
H	-10.57158971	5.15801657	46.74413807
C	7.20394862	1.90405501	42.33039048
H	6.72514760	1.04731027	41.88371338
C	-3.28041557	4.70007305	31.00645357
H	-2.80036436	3.84791273	31.47957195
C	-2.55580270	12.40422986	36.89724147
H	-2.34203284	11.80613874	36.02443127
C	-6.38818576	6.18411081	35.00148698
H	-6.52482735	6.69632871	35.94196769
C	-7.92195644	8.27336512	46.12997433
C	-9.30054086	12.44884601	44.89116664
H	-10.34968776	12.24731501	44.73775388
C	-8.06491735	10.07845248	35.96341158
H	-7.88283079	9.49533196	35.07216940
C	-10.87228576	-2.75958890	46.18102500
H	-11.55588779	-2.31497804	46.88772896
C	6.93229938	2.93972432	46.54435114

H	7.56185245	2.65592414	45.71591102
C	-5.99196459	20.78023854	42.16256434
H	-5.69944568	21.64729895	41.59169481
C	-6.68837849	15.46838950	47.02874482
H	-6.65757764	14.66994370	47.76503152
C	-11.35099848	8.81651610	41.25586576
H	-12.00508153	8.44533705	40.48253132
C	-3.27783091	11.00533191	47.08303137
H	-4.05007542	11.76097073	47.08954767
C	-6.71887779	7.72544379	31.01058003
H	-7.69583261	7.73716070	31.48584667
C	-4.06734951	4.51384766	29.82033702
C	3.00779159	9.09105268	44.87888064
H	3.35942865	10.10086775	44.73271161
C	-13.34552115	-0.23284735	46.62823563
H	-13.91030734	-0.64234959	45.80497958
C	-14.25524413	-2.23241464	42.79193287
H	-14.61196489	-3.24611081	42.70814098
C	0.89736958	12.69935131	41.25374666
H	0.90562676	13.45481683	40.48377671
C	8.56187253	2.12690426	42.17730737
H	9.17412559	1.44644720	41.60699420
C	-9.16012782	-3.83843183	44.36032614
H	-8.48547159	-4.22370074	43.61243436
C	-14.93136434	-1.18386826	42.18095972
H	-15.82867270	-1.36392175	41.61029453
C	-9.33247387	10.09739685	36.53883141
H	-10.13156342	9.53723394	36.07862791
C	-4.63610995	2.36231690	35.97026611
H	-4.22364616	2.81039044	35.07775532
C	9.11730889	3.24843078	42.78030920
H	10.16976776	3.46273569	42.68979647
C	-5.13383530	19.70345455	42.30935677
H	-4.15455842	19.70539942	41.85776215
C	7.94789615	8.34321262	44.37635949
H	7.95677620	9.11726929	43.62566140
C	-11.03863914	10.16249616	41.29451134
H	-11.45083108	10.82873848	40.55261951
C	7.83590313	6.32748767	46.20186782
H	7.78207033	5.51629923	46.91056877
C	1.90623155	11.75510750	41.29095896
H	2.69124493	11.78177137	40.55119393
C	-7.23863915	20.71815380	42.77232352
H	-7.93891413	21.53314264	42.68710989
C	-9.38645214	18.05264772	46.16273226
H	-8.66047327	18.42363997	46.86966155
C	-1.29332848	9.00954539	47.08819434
H	-0.53244197	8.24409868	47.10489741
C	-7.82012868	6.04930036	47.10021963
H	-8.08833851	5.00266658	47.10555471
C	-6.48899373	8.49724193	29.82214936
C	7.08794202	2.32469105	47.77816661
H	7.83573238	1.55899008	47.90583086
C	0.97616776	10.26941840	36.54570995
H	0.88896282	11.24223221	36.08732748
C	8.81407683	8.38176553	45.45581318
H	9.52038941	9.18862601	45.57080275
C	-4.92096426	9.07695263	28.25234415
H	-3.90948367	8.98289366	27.89004782

C	-11.17466199	17.10576710	44.34173052
H	-11.84474760	16.71287794	43.59369427
C	-9.58258527	10.83617621	37.68001588
H	-10.57672238	10.84548266	38.09845299
C	-7.08452644	8.76595809	47.10838105
H	-6.80246402	9.80770746	47.12624667
C	-5.51531667	19.36785615	47.85214957
H	-5.23563664	20.39945740	47.99153690
C	-3.97572711	3.86853532	47.12801076
H	-5.01893214	3.59197702	47.14658807
C	-1.25519663	4.59020822	47.11819859
H	-0.21462711	4.88128867	47.12263003
C	-3.45222722	4.70627428	48.11012672
H	-4.09714932	5.07764874	48.89101202
C	-13.94485220	-0.06084422	47.86797274
H	-14.97822188	-0.33367337	48.00782524
C	1.73918151	10.11631607	37.68806718
H	2.24090203	10.97303643	38.10973146
C	-6.97337038	6.55778248	48.08137011
H	-6.60148929	5.90097275	48.85241435
C	-2.11244660	6.95154521	28.25044054
H	-2.69944836	6.12102140	27.89146882
C	8.75271042	7.35224378	46.38657981
H	9.40751859	7.33892768	47.24263533
C	6.27480824	2.71290884	48.83555204
H	6.37657360	2.25574723	49.80703689
C	-6.61769212	7.89349004	48.08878607
H	-5.97116040	8.26596818	48.86778658
C	-3.26259486	10.01462599	48.06107788
H	-4.01850351	10.01888711	48.83099389
C	-10.44179786	-4.06727780	46.35508850
H	-10.78836823	-4.63910864	47.20051143
C	-2.28415406	9.03827645	48.06685516
H	-2.28657734	8.28900783	48.84284348
C	-5.43955917	18.45888566	48.89995668
H	-5.09958284	18.76844995	49.87557934
C	-9.56836763	-4.62078820	45.42738163
H	-9.21976268	-5.63575718	45.53483304
C	-4.01710484	1.25654879	36.54667660
H	-3.13267610	0.84460454	36.08607176
C	-2.11759600	5.06602513	48.10215496
H	-1.73356168	5.71367484	48.87501625
C	-13.19511960	0.46021519	48.91499660
H	-13.63302895	0.60148886	49.89045900
C	-11.87158093	0.78941935	48.67578301
H	-11.24778440	1.19398419	49.45689886
C	-4.52983368	0.67206642	37.68953912
H	-4.03948515	-0.19238627	38.10878438
C	-5.35868774	5.58237333	28.25519249
H	-5.78423002	6.50546375	27.89461344
C	-5.81518076	17.14765009	48.66133741
H	-5.77715527	16.40577925	49.44308102
C	-11.64950667	17.85138553	45.40752470
H	-12.70302811	18.05641412	45.51381405
C	5.33385450	3.70336181	48.61062937
H	4.68009584	4.04141868	49.39873385
C	-10.73456079	18.33276320	46.33545454
H	-11.05766105	18.91942407	47.18001349
C	-7.46752514	9.26837934	29.19368846

H	-8.46419623	9.31038870	29.60317439
C	-0.67398072	9.06529454	29.18355350
H	-0.13937720	9.90906610	29.58981541
C	-4.24787114	3.28103507	29.19204923
H	-3.78450772	2.39679446	29.59974060
C	-5.83423669	9.86487996	27.56680573
H	-5.52954590	10.38697066	26.67440659
C	-0.97488510	7.34763386	27.56200888
H	-0.67594534	6.82045153	26.67065705
C	-7.13545407	9.96359471	28.04333655
H	-7.86811712	10.56431725	27.52809098
C	-5.58663185	4.39768841	27.57003874
H	-6.19413451	4.40073516	26.67968914
C	-0.23912843	8.42724700	28.03431746
H	0.64636183	8.76074073	27.51679742
C	-5.01982611	3.22132274	28.04426711
H	-5.17545167	2.28659192	27.52929155

9. References

(1) Sibi, M. P.; Petrovic, G. Enantioselective Radical Reactions: The Use of Metal Triflimides as Lewis Acids. *Tetrahedron Asymmetry* **2003**, *14*, 2879–2882.

(2) Sprachmann, J.; Wachsmuth, T.; Bhosale, M.; Burmeister, D.; Smales, G. J.; Schmidt, M.; Kochovski, Z.; Grabicki, N.; Wessling, R.; List-Kratochvil, E. J. W.; Esser, B.; Dumele, O. Antiaromatic Covalent Organic Frameworks Based on Dibenzopentalenes. *J. Am. Chem. Soc.* **2023**, *145*, 2840–2851.

(3) Vizintin, A.; Bitenc, J.; Kopač Lautar, A.; Pirnat, K.; Grdadolnik, J.; Stare, J.; Randon-Vitanova, A.; Dominko, R. Probing Electrochemical Reactions in Organic Cathode Materials via in Operando Infrared Spectroscopy. *Nat. Commun.* **2018**, *9*, 661.

(4) Ronson, T. K.; League, A. B.; Gagliardi, L.; Cramer, C. J.; Nitschke, J. R. Pyrene-Edged $Fe^{II}_4L_6$ Cages Adaptively Reconfigure During Guest Binding. *J. Am. Chem. Soc.* **2014**, *136*, 15615–15624.

(5) Ronson, T. K.; Meng, W.; Nitschke, J. R. Design Principles for the Optimization of Guest Binding in Aromatic-Paneled $Fe^{II}_4L_6$ Cages. *J. Am. Chem. Soc.* **2017**, *139*, 9698–9707.

(6) Allan, D. R.; Nowell, H.; Barnett, S. A.; Warren, M. R.; Wilcox, A.; Christensen, J.; Saunders, L. K.; Peach, A.; Hooper, M. T.; Zaja, L.; Patel, S.; Cahill, L.; Marshall, R.; Trimnell, S.; Foster, A. J.; Bates, T.; Lay, S.; Williams, M. A.; Hathaway, P. V.; Winter, G.; Gerstel, M.; Wooley, R. W. A Novel Dual Air-Bearing Fixed-χ Diffractometer for Small-Molecule Single-Crystal X-Ray Diffraction on Beamline I19 at Diamond Light Source. *Crystals* **2017**, *7*, 336.

(7) Sheldrick, G. M. SHELXT – Integrated Space-Group and Crystal-Structure Determination. *Acta Crystallogr. Sect. Found. Adv.* **2015**, *71*, 3–8.

(8) Sheldrick, G. M. Crystal Structure Refinement with SHELXL. *Acta Crystallogr. Sect. C Struct. Chem.* **2015**, *71*, 3–8.

(9) Dolomanov, O. V.; Bourhis, L. J.; Gildea, R. J.; Howard, J. a. K.; Puschmann, H. OLEX2: A Complete Structure Solution, Refinement and Analysis Program. *J. Appl. Crystallogr.* **2009**, *42*, 339–341.

(10) Maglic, J. B.; Lavendomme, R. MoloVol: An Easy-to-Use Program for Analyzing Cavities, Volumes and Surface Areas of Chemical Structures. *J. Appl. Crystallogr.* **2022**, *55*, 1033–1044.

(11) Bannwarth, C.; Ehlert, S.; Grimme, S. GFN2-xTB—An Accurate and Broadly Parametrized Self-Consistent Tight-Binding Quantum Chemical Method with Multipole Electrostatics and Density-Dependent Dispersion Contributions. *J. Chem. Theory Comput.* **2019**, *15*, 1652–1671.

(12) Yamashina, M.; Tanaka, Y.; Lavendomme, R.; Ronson, T. K.; Pittelkow, M.; Nitschke, J. R. An Antiaromatic-Walled Nanospace. *Nature* **2019**, *574*, 511–515.

(13) Gaussian 16, Revision C.01, M. J. Frisch, G. W. Trucks, H. B. Schlegel, G. E. Scuseria, M. A. Robb, J. R. Cheeseman, G. Scalmani, V. Barone, G. A. Petersson, H. Nakatsuji, X. Li, M. Caricato, A. V. Marenich, J. Bloino, B. G. Janesko, R. Gomperts, B. Mennucci, H. P. Hratchian, J. V. Ortiz, A. F. Izmaylov, J. L. Sonnenberg, D. Williams-Young, F. Ding, F. Lipparini, F. Egidi, J. Goings, B. Peng, A. Petrone, T. Henderson, D. Ranasinghe, V. G. Zakrzewski, J. Gao, N. Rega, G. Zheng, W. Liang, M. Hada, M. Ehara, K. Toyota, R. Fukuda, J. Hasegawa, M. Ishida, T. Nakajima, Y. Honda, O. Kitao, H. Nakai, T. Vreven, K. Throssell, J. A. Montgomery, Jr., J. E. Peralta, F. Ogliaro, M. J. Bearpark, J. J. Heyd, E. N. Brothers, K. N. Kudin, V. N. Staroverov, T. A. Keith, R. Kobayashi, J. Normand, K. Raghavachari, A. P. Rendell, J. C. Burant, S. S. Iyengar, J. Tomasi, M. Cossi, J. M. Millam, M. Klene, C. Adamo, R. Cammi, J. W. Ochterski, R. L. Martin, K. Morokuma, O. Farkas, J. B. Foresman, and D. J. Fox, Gaussian, Inc., Wallingford CT, 2016.

(14) Guenich, D.; Hess, K.; Köhler, F.; Herges, R. Anisotropy of the Induced Current Density (ACID), a General Method To Quantify and Visualize Electronic Delocalization. *Chem. Rev.* **2005**, 105, 3758–3772.
